# Supplementary material for: Cost-effectiveness and budget impact analysis of facility-based screening and treatment of hepatitis C in Punjab state of India
Source: BMJ Open. 2021 Feb 15;11(2):e042280. doi: 10.1136/bmjopen-2020-042280 (PMC7887370; doi:10.1136/bmjopen-2020-042280)
Supplement: Author's manuscript [file bmjopen-2020-042280.draft_revisions.pdf]

BMJ Open is committed to open peer review. As part of this commitment we make the peer review history of every article we publish publicly available.

When an article is published we post the peer reviewers' comments and the authors' responses online. We also post the versions of the paper that were used during peer review. These are the versions that the peer review comments apply to.

The versions of the paper that follow are the versions that were submitted during the peer review process. They are not the versions of record or the final published versions. They should not be cited or distributed as the published version of this manuscript.

BMJ Open is an open access journal and the full, final, typeset and author-corrected version of record of the manuscript is available on our site with no access controls, subscription charges or pay-per-view fees (http://bmjopen.bmj.com).

If you have any questions on BMJ Open's open peer review process please email info.bmjopen@bmj.com

BMJ Open

Cost-effectiveness and budget impact analysis of screening and treating Hepatitis C infected population vs no screening and treatment in Punjab state of India.

Journal:	*BMJ Open*
Manuscript ID	bmjopen-2020-042280
Article Type:	Original research
Date Submitted by the Author:	01-Jul-2020
Complete List of Authors:	Chugh, Yashika; Post Graduate Institute of Medical Education and Research, Community Medicine and School of Public Health<br>Kumar, Madhumita ; Post Graduate Institute of Medical Education and Research, Department of Hepatology<br>Grover, Gagandeep<br>Dhiman, Radha K.; Sanjay Gandhi Post Graduate Institute of Medical Sciences<br>Teerawattananon, Yot; HITAP<br>Prinja, Shankar; Post Graduate Institute of Medical Education and Research, Community Medicine
Keywords:	HEALTH ECONOMICS, Public health < INFECTIOUS DISEASES, Health policy < HEALTH SERVICES ADMINISTRATION & MANAGEMENT

SCHOLARONE™
Manuscripts

I, the Submitting Author has the right to grant and does grant on behalf of all authors of the Work (as defined in the below author licence), an exclusive licence and/or a non-exclusive licence for contributions from authors who are: i) UK Crown employees; ii) where BMJ has agreed a CC-BY licence shall apply, and/or iii) in accordance with the terms applicable for US Federal Government officers or employees acting as part of their official duties; on a worldwide, perpetual, irrevocable, royalty-free basis to BMJ Publishing Group Ltd ("BMJ") its licensees and where the relevant Journal is co-owned by BMJ to the co-owners of the Journal, to publish the Work in this journal and any other BMJ products and to exploit all rights, as set out in our licence.

The Submitting Author accepts and understands that any supply made under these terms is made by BMJ to the Submitting Author unless you are acting as an employee on behalf of your employer or a postgraduate student of an affiliated institution which is paying any applicable article publishing charge ("APC") for Open Access articles. Where the Submitting Author wishes to make the Work available on an Open Access basis (and intends to pay the relevant APC), the terms of reuse of such Open Access shall be governed by a Creative Commons licence – details of these licences and which Creative Commons licence will apply to this Work are set out in our licence referred to above.

Other than as permitted in any relevant BMJ Author's Self Archiving Policies, I confirm this Work has not been accepted for publication elsewhere, is not being considered for publication elsewhere and does not duplicate material already published. I confirm all authors consent to publication of this Work and authorise the granting of this licence.

For peer review only

Title: Cost-effectiveness and budget impact analysis of screening and treating Hepatitis C infected population vs no screening and treatment in Punjab state of India.

Authors: Yashika Chugh[1], Madhumita Premkumar[2], Gagandeep Singh Grover[3], Radha Krishan Dhiman[4,5], Yot Teerawattananon[6], Shankar Prinja[1]

Institution:

1. Department of Community Medicine and School of Public Health, Post Graduate Institute of Medical Education and Research, Chandigarh, India
2. Department of Hepatology, Post Graduate Institute of Medical Education and Research, Chandigarh, India
3. State Program Officer, Hepatitis C Virus Infection, Directorate of Health and Family Welfare, Punjab, India
4. Director, Sanjay Gandhi Postgraduate Institute of Medical Sciences, Lucknow, Uttar Pradesh, India.
5. Chairman, Technical Resource Group - National Viral Hepatitis Control Program (NVHCP), Government of India, India
6. Health Intervention and Technology Assessment Program (HITAP), Ministry of Public Health, Nonthaburi, Thailand & Saw Swee Hock School of Public Health, National University of Singapore.

Short title:
Cost-Effectiveness of universal screening for hepatitis C.

Key words:
cost effectiveness analysis, hepatitis C virus, universal screening, ELISA, RDT, budget impact.

Manuscript details:
415 words abstract, 4237 words main text, 3 tables, 3 figures, 61 references.

Funding:
The Health Intervention and Technology Assessment Program (HITAP) provided technical supervision for this study under the auspices of the International Decision Support Initiative (iDSI). HITAP's International Unit is supported by iDSI to provide technical assistance on health intervention and technology assessment to governments in low- and middle-income countries. iDSI is funded by the Bill & Melinda Gates Foundation [OPP1134345], the United Kingdom's Department for International Development, and the Rockefeller Foundation. The findings, interpretations and conclusions expressed in this article do not necessarily reflect the views of the funding agencies.

Conflicts of interest:
No conflicts of interest declared

Ethical Approval:
The present study is a model-based analysis which did not involve any human subjects as well as primary data collection. Therefore, ethical approval was not required. Administrative approval was sought from Department of Health & Family Welfare, Punjab, India.

For peer review only - http://bmjopen.bmj.com/site/about/guidelines.xhtml

Corresponding author:
Dr. Shankar Prinja
Additional Professor of Health Economics
Department of Community Medicine and School of Public Health, Post Graduate Institute of Medical Education and Research
Sector-12, Chandigarh – 160012
Ph: +91 9872871978
Email: shankarprinja@gmail.com

For peer review only

For peer review only - http://bmjopen.bmj.com/site/about/guidelines.xhtml

Cost-effectiveness and budget impact analysis of screening and treating Hepatitis C infected population vs no screening and treatment in Punjab state of India.

Abstract

Objective: To assess incremental cost per quality adjusted life year (QALY) for one-time universal screening followed by treatment of Hepatitis C Virus (HCV) infected population as compared to no-screening policy in Punjab, India.

Methodology: Decision tree integrated with Markov model was developed to simulate disease progression. Lifetime costs and outcomes were modelled for three scenarios – no universal screening and treatment (routine care), scenario I – screening with Enzyme Linked Immunosorbent Assay (ELISA) followed by confirmatory HCV-Ribonucleic Acid (RNA) quantification and scenario II – screening with Rapid diagnostic test (RDT) kit followed by confirmatory HCV-RNA quantification. A societal perspective and a 3% annual discount rate were considered to assess incremental cost per QALY gained. In addition, budgetary impact was also assessed.

Results: Both scenarios I and II dominate over no-screening policy. Screening with ELISA and RDT, respectively, results in a gain of 0.028 (0.008-0.06) and 0.027 (0.008-0.061) QALY per person with costs decreased by □-1,810 (-3,376 to – 867) and □-1,812 (-3,468 to -850) when compared with no-screening. Screening the age cohort of 40-45 years at a coverage of 30%, with ELISA and RDT, would cost 209 and 190 million rupees, respectively.

Conclusion: One-time universal screening followed by HCV treatment is a dominant strategy as compared to no-screening. However, budget impact of screening activity seems unsustainable. Thus, in view of findings from both cost-effectiveness and budget impact, we recommend beginning with screening the age cohort with RDT around mean age of disease presentation instead of whole population.

For peer review only

For peer review only - http://bmjopen.bmj.com/site/about/guidelines.xhtml

Article Summary

Strengths and limitations of this study

Strengths

- We used a biologically plausible model which is fitted with local data on disease epidemiology and effectiveness of DAAs which is drawn from the program data of 48,808 HCV patients on treatment in Punjab, India.
- Secondly, cost of treatment is drawn from the National Health System Cost Database and nationally representative household surveys for patient costs.
- Thirdly, the data on quality of life is also based on our analysis of primary data collected locally.

Limitations

- We assumed the coverage estimated for screening to be 30% in the base case as there is no active screening program in operation which could provide us with a realistic estimate. However, we varied the coverage estimate in our PSA from 10 to 50%. Also, we conducted a univariate analysis to report ICER/QALY at different coverage levels for 10 to 100%.
- Secondly, high risk group (HRG) screening assessment would have been a better strategy to generate evidence as India is a resource limited setting and prevalence among HRGs is high. However, our results demonstrate that even universal screening among healthy population is cost saving thus it is highly suggestive that screening among HRG would be even more cost saving.
- Thirdly, we did not consider the possibility of reinfection once treated in our model as we were estimating the impact of one-time screening and treatment. Therefore, we assumed that including reinfection would not change the direction of results.
- Fourthly, we did not include productivity losses in our cost analysis.

For peer review only - http://bmjopen.bmj.com/site/about/guidelines.xhtml

For peer review only

Introduction:

Nearly 71 million people are living with Hepatitis C virus (HCV) infection round the globe.[1,2] HCV infection led to around 399,000 deaths mainly caused by cirrhosis and hepatocellular carcinoma (HCC).[2] With the advent of Directly Acting Antivirals (DAA), it is possible to cure 90% of the people infected with HCV, however, the access to diagnostics and treatment facilities is quite low.[2,3] Globally, only 1 in 5 people living with HCV infection had been diagnosed.[4]

The current diagnosis rate being less than 5% and treatment coverage less than 1%[5], the World Health Assembly (WHA) released the global targets to eliminate viral hepatitis by 2030.[6] The aim is to diagnose 90% of the Hepatitis B virus (HBV) and HCV infections and treat 80% of the eligible infections by 2030.[6,7] With around 40% of the HCV infected people residing in low and middle income countries (LMICs), India happens to be one of the major contributors to the global burden where only less than 1% of people are aware of their infection status.[4,8]

Though the national prevalence in India ranges from 0.9-1.9%,[9-11] the burden is as high as 3.6% in the northern state of Punjab.[12-14] The high prevalence in Punjab can be attributed to the higher prevalence of unsafe medical practices and rather than due to intravenous drug use (IDU) , therefore posing a high risk of reinfection as well.[12] Thus, along with implementation of treatment strategies there is a dire need to focus on screening, diagnosing those with HCV infection and population education strategies to break the chain of transmission.

In 2016, Punjab government launched the "Mukh Mantri Punjab Hepatitis C Relief Fund (MMPHCRF)" for free of charge universal treatment of HCV patients with all oral DAAs.[15] Despite free treatment, due to lack of awareness of disease status, only 10% of the total estimated cases in Punjab have been put on treatment.

For peer review only

For peer review only - http://bmjopen.bmj.com/site/about/guidelines.xhtml

With the success of the Punjab Model and in concurrence with the global effort to eliminate HCV infection, India launched the National Viral Hepatitis Control Programme (NVHCP) in July 2018 which aims at improving access to diagnosis and treatment for viral hepatitis.

The state government plans to move forward with screening in a phased manner. Screening could be done among high risk groups like IDUs, people living with Human Immunodeficiency virus (PLHIV), patients on haemodialysis or haemophiliacs. Another strategy could be screening around the mean age of presentation of the infection which is around 40 years from the data base of the 48,088 patients treated under MMPHCRF.[16]

To inform this policy decision on the implementation of a screening cum treatment program in the state of Punjab, we undertook this analysis to assess the incremental cost per quality adjusted life year (QALY) for one-time universal screening followed by treatment of HCV infected population as compared to no screening in Punjab. This study also aims to inform on which screening technology represents value for money and would yield a better financial sustainability for population-based screening in Punjab. Thus, a budget impact analysis was performed in addition to estimate the financial resources required in terms of initiating a screening program in the state.

Methods:

Overview of Analysis

Screening services were assumed to be delivered as a facility-based intervention wherein active screening for HCV cases would be performed at 22 district hospitals in the state of Punjab which will act as integrated testing as well as treating sites for HCV. Since, screening is to be initiated in the state, this assumption was made in consultation with experts from "*National Taskforce to Combat Viral Hepatitis*". We modelled the lifetime costs and outcomes for screening and treating HCV infected patients in Punjab considering 3 different scenarios – no universal screening and

For peer review only - http://bmjopen.bmj.com/site/about/guidelines.xhtml

For peer review only

treatment of those detected in 'routine care' setting, scenario I – screening with Enzyme Linked Immunosorbent Assay (ELISA) followed by confirmatory HCV-Ribonucleic Acid (RNA) quantification, and scenario II – screening with Rapid diagnostic test (RDT) kit followed by confirmatory HCV-RNA quantification. A lifelong time horizon was considered so as to account for all costs and consequences of the intervention for chronic HCV. The outcomes are valued in terms of the number of HCV deaths, life years and QALYs. All the future costs and consequences are discounted at a rate of 3%. This choice of discounting rate is based on standard international guidelines along with being consistent with other Indian economic evaluations.[17-20] The methodological principles are in concurrence with the Indian reference case for conducting economic evaluations published by the health technology agency in India.[20]

No screening and treatment/ Routine-care scenario

The routine care scenario represents the current situation where-in there is no population-based screening. In this situation, we assume that patients present to the facility of their own volition when symptoms develop and access the required management support. Since the treatment coverage in India is low[1,21], we also assume that only 5% of the HCV infected cohort receives the testing and treatment on their own and the rest progress to advance stages of HCV as per the natural progression of the disease. The drug regimen followed to treat those infected is as per the national guidelines for diagnosis and management of HCV in India (Figure 1). The rest 95% patients who become aware of their status at later stages with presentation of cirrhosis, incur the cost of supportive management including the management of associated complications.

Figure 1.

Screening with ELISA - Scenario I

In this scenario, ELISA was used as the screening test followed by HCV RNA as the confirmatory step. The coverage of screening has been assumed to be 30% based on the uptake of screening in

For peer review only - http://bmjopen.bmj.com/site/about/guidelines.xhtml

other programs[22-24] Those who are then determined eligible, are administered treatment as per the algorithm. A loss to follow up of 10% has been assumed at each step from testing to treatment. Currently, ELISA is being used at the facility level for testing antibodies to HCV. This scenario is compared with the do-nothing scenario wherein there is no active screening program in practice.

Screening with RDT – Scenario II

This scenario differs from the above in the test used for screening. RDT kits are considered instead of ELISA for testing the antibodies to HCV followed By HCV RNA as the confirmatory step. The rationale for comparing both these testing strategies is that use of ELISA based screening tests is associated with long turn-around time, high cost and requirements for specialized apparatus and trained technicians whereas, screening with RDTs obviates the need for multiple follow-up appointments as well as shortens the waiting time. Also, RDT follow a simpler technique as compared to ELISA based screening.[25]

The coverage and loss to follow up assumptions remain the same for this scenario as well. This scenario is also compared with the no screening scenario.

Model structure

A decision tree (Figure 2 A) integrated with a mathematical Markov model (Figure 2 B), depicting natural history of HCV was prepared in Microsoft Excel to simulate the progression of disease. The costs and health outcomes of each intervention scenario (I and II) are compared with the routine care scenario.

Figure 2 A.

A Markov model, with similar transition states, used in a previous analysis to assess the cost effectiveness of using velpatasvir (VEL) for treatment of HCV, was constructed to simulate disease progression among the HCV population.[26,27]

For peer review only - http://bmjopen.bmj.com/site/about/guidelines.xhtml

Figure 2 B.

A cross-sectional study in Punjab which included 5,543 individuals, reported a prevalence of 3.6% with a viremia rate of 70% thus estimating a burden of around 700,000 viraemic hepatitis C patients.[12-14] Another cross-sectional study reports age-based prevalence estimated for Punjab.[14] Based on the analysis of the MMPHCRF patient data, the mean age for HCV infection was estimated to be around 40 years.[16] Thus, in our analysis, we considered one-time universal screening of the Punjab population around the mean age of presentation of HCV with prevalence at 4.7 %. Subsequent to treatment, the patients were divided into those who achieve sustained virologic response (SVR) and others who do not. Out of those who achieve SVR, if in stages F0-F3, they do not progress further and thus the progression is halted. For the patients with cirrhosis who achieve SVR, they continue to progress to subsequent stages but at a slower rate as compared to the natural progression rates. Those patients who do not respond to the treatment and thus do not achieve SVR, continue to progress to subsequent stages as per the natural progression of the disease. It was assumed that death due to HCV occurs from decompensated cirrhosis and HCC states while all-cause mortality happens in all stages. A cycle length of 1 year was considered appropriate along with a life-time study horizon to comprehensively capture all the costs and benefits of the intervention.

Costing

A societal perspective was considered to analyse total costs for screening and treatment. Treatment associated costs include cost of out-patient (OPD) consultation for therapeutic purpose, in-patient care for hospitalisation which is required in advanced stages as well as cost of management of complications. The cost of specific procedures required in assessment of fibrosis and at later stages such as endoscopy, transient elastography, ultrasound, ascitic tap were also included. Average costs for primary, secondary and tertiary OPD consultation and hospitalisation

For peer review only - http://bmjopen.bmj.com/site/about/guidelines.xhtml

as reported in recently published Indian studies, as well as National Health System Cost Database were used (Table 1).[28-30] The tertiary sector costs for IPD included both the cost of medicine ward[31] as well as the intensive care unit (ICU) costs. The cost of ELISA and RDT kits were as per the procurement rates of the Punjab government obtained via consultations with program officers. Other costs associated with the test such as cost of equipment, human resource, infrastructures, disposables etc. was available from data collected for costing the laboratory procedures at the district hospital. The confirmatory test HCV RNA was outsourced by the government and thus the provider payment rates of Punjab government contracted laboratories were used.[15] The cost of DAAs (ranging from □ 4000-17000, US $ 57-242 per patient) were also as per the procurement prices in Punjab.[15] All costs are also reported for the current year in USD at a currency exchange rate of 1 USD = 70.5 INR.[32]

We also included the out of pocket (OOP) expenditure of the patient for OPD and hospitalisation in public and private facilities. It was derived based on analysis of unit level data from National Sample Survey 71st round, conducted in during January-June 2014, which is a large household survey covering a sample of 65,932 households.[33]

Valuation of consequences

The impact of intervention was dependent on the sensitivity and specificity of the screening tests as well as SVR rates achieved with the treatment provided subsequently. The estimates for sensitivity and specificity of ELISA and RDT fed into the model have been reported by published systematic reviews and meta-analysis.[34,35] The impact of treatment has been reported in terms of SVR which was modelled to assess progression to more severe health states and finally in terms of survival. The health-related quality of life was measured using Euro-QOL five dimensions questionnaire (EQ-5D-5L) which was administered to a total of 230 patients who were being treated for chronic HCV infection.[26] We assumed that the quality of life among non-cirrhotic

For peer review only

For peer review only - http://bmjopen.bmj.com/site/about/guidelines.xhtml

patients who achieve SVR to be equivalent to the general population[36] whereas for the cirrhotic and HCC patients, we assumed that the quality of life score was similar as in the respective advanced stages.

The estimates for SVR rates were obtained from MMPHCRF data and are consistent with the estimates used in the previous analysis.[16,26] All the parameters pertaining to progression of HCV, with and without achieving SVR, were obtained through published literature.[37,38] Annual mortality estimates from DC and HCC were 21.6% and 41.1% respectively and were reported from published literature.[39-41] The age-wise all-cause mortality was obtained from the Sample Registration System report for Punjab state.[42]

Also, we made an assumption regarding the coverage of screening to be 30% in the base case.[23-25] Since, there is no population-based screening program currently running in the state, therefore we tried to put in a modest estimate which is not very high as well as will help to reflect the impact of screening activity.

All the parameters related to costs and effectiveness have been reported in Table 1.

Sensitivity Analysis

We undertook a PSA to analyse the effect of joint parameter uncertainty on the ICER.[43,44] For several parameters, 95% confidence intervals were available such as age-wise prevalence estimates, progression parameters, sensitivity and specificity of screening tests. For other parameters including the stage-wise distribution at the time of diagnosis and SVR rates were varied by 5% for both upper and lower limits, RBV tolerance was varied by 10% for both upper and lower limits. All the drugs costs were varied by 20% on the lower side only as the current prices of DAA are the negotiated prices resulting from operating bulk procurement mechanisms in the state. Therefore, the prices might decrease further but the increase in the prices is unlikely. The

For peer review only - http://bmjopen.bmj.com/site/about/guidelines.xhtml

cost of screening tests was varied 20% on the lower side and 50% on the upper side. For quality of life estimates, standard errors were computed form the primary analysis.

For undertaking the PSA, gamma distribution was used for all the cost parameters, a beta distribution was used in case of parameters where 95% confidence intervals were available, and a uniform distribution was applied where the upper and lower limits were available. Monte Carlo method was used, and the results were simulated 1000 times. We computed the median estimates along with 2.5th and 97.5th percentile to estimate 95% confidence interval.

Based on the PSA, we estimated the probability of being cost-effective at varying willingness to pay thresholds (WTP) for scenario I and II compared with no screening. We used a 1- time per-capita Gross Domestic Product (GDP) value for India as the threshold to base our conclusion about cost-effectiveness. This was in view of the national guidelines for Health Technology Assessment (HTA)[20], current practice in Indian economic evaluations[19,45] and more national discussions.[46]

A scenario analysis was conducted for assessing the cost effectiveness of scenario I and II as compared to the routine care, i.e, no-screening and treatment, wherein we modelled the impact of screening and treatment for all people aged 18 years and above. Thus, this scenario analysis primarily evaluated the cost effectiveness of screening which could be attributed to difference in age of initiating the screening.

We also conducted a univariate analysis to assess the impact of varying screening coverage parameter. Thus, we estimated ICERs at coverage rates of 10%, 20%, 50%, 70% and 100%.

Budget Impact Analysis

We undertook a budget impact analysis to assess the requirement of resources for the uptake of screening and treatment program. Two scenarios were considered, screening everyone aged 18 years and above and screening the age cohort of 40-45 years which is the mean age of presentation

For peer review only - http://bmjopen.bmj.com/site/about/guidelines.xhtml

of HCV infection. The parameters considered were: Punjab population estimates from census[47], prevalence estimates from published literature[12-14], cost of screening tests which included ELISA and RDT based on expert consultations with program officers, cost of confirmatory test HCV RNA[15], cost of drugs given for treatment as per the provider payment rates of Punjab government[15], cost incurred on human resources in terms of their salary as well as cost of integrated trainings conducted to train personnel for carrying out screening activities and the cost spent on information-education-counselling (IEC) and behaviour change communication (BCC) activities. The cost of integrated trainings and IEC/BCC activities were derived from expert consultations with program officers and the review of state budget documents.

For the human resources, we estimated the man-hours currently being dedicated to HCV screening and therefore we budgeted in terms of additional man-hours required to screen a given proportion of population if screening facilities were to be delivered via existing set up. Cost of training and IEC/BCC have been treated as fixed costs as there is no basis for determining repeated IEC/BCC activities in absence of an active screening program. The cost of screening test, confirmatory test and drugs are the variable costs associated with screening and treatment.

A payer's perspective was considered for the analysis with no discounting and a time horizon of five years. Results were reported at different coverage rates of screening 10 to 100%. The methodology of the budget impact analysis was in consistency with International Society for Pharmacoeconomics and Outcomes Research (ISPOR) reporting framework.[48]

Patient and public involvement

No patient and public involved.

Results:

Costs

For peer review only - http://bmjopen.bmj.com/site/about/guidelines.xhtml

In the no screening policy, a lifetime cost of □ 2,775 (US $ 38.8) is incurred per person. In scenario I, which is screening the population with ELISA, a lifetime societal cost of □ 998 (US $ 14.1) is incurred per person. If we consider RDT instead of ELISA for screening, i.e, scenario II, □ 947(US $ 13.2) per person. When scenario I is compared with no screening option, it saves □ 1,810 (US $ 25.3) per person. The cost savings are similar when scenario II is compared with no screening, i.e, □ 1,812 (US $ 25.3) per person.

Valuation of consequences

The life years lived by Punjab population including HCV infected cohort as well in no screening, scenario I and II were 15.75, 15.92 and 15.76 per person respectively. Similarly, the QALYs lived per person in no screening, scenario I and II were 15.57, 15.78 and 15.6 respectively. As compared to the no screening scenario, there was a gain of 0.0046 (0.0013-0.011) life years and 0.028 (0.008-0.06) QALY per person in scenario I. The gains are similar when scenario II is compared with no screening, i.e, 0.0045 (0.0012-0.012) life years and 0.027 (0.008-0.061) QALY per person.

Costs and outcomes for each scenario have been given in Table 2.

Cost-effectiveness

When compared with no screening, both scenarios, i.e screening with ELISA as well RDT, dominate over no screening, i.e, they have better health outcomes along with being cost saving. However, when the incremental gains of scenario I and II (relative to no screening) are compared, there is no statistically significant difference in outcomes as well as costs.

Screening is cost saving at all coverage levels from 10 to 100%. Scenario I and II (relative to no-screening), have a 100% probability of being cost saving. Also, screening with both ELISA and RDT for people aged 18 years and above is cost saving. This implies that irrespective of the age of initiation of screening and subsequent treatment, screening is a dominant strategy as compared to

For peer review only

For peer review only - http://bmjopen.bmj.com/site/about/guidelines.xhtml

no screening. Figure 3 displays cost-effectiveness planes for both the scenarios (ELISA and RDT) as compared to no screening.

Figure 3.

Budget Impact Analysis

The budget impact includes 5 heads: cost of human resources including training activities for conducting screening, IEC/BCC for motivation of population, screening test costs, confirmatory test cost and drug treatment cost. In addition, we also included the additional health care costs incurred due to increased survival of HCV infected patients as a result of the intervention.

The year wise disaggregated results from the 1st to 5th year, distribution of budget estimates for the base year at 30% coverage according to type of resources and budget estimates for different coverage levels are summarised in Table 3.

Discussion:

In resource limited settings like India, where cost of providing healthcare is continuously rising, it is of utmost importance to utilise the resources in a sustainable manner. The present analysis, thus, assesses the sustainability of a screening and treatment program for HCV. Our findings demonstrate that screening, either with ELISA or RDT, is dominant over no screening which implies having higher QALYs gained and lower costs. Also, the difference between incremental gains for both scenario I and II (compared to no screening) is insignificant which can be attributed to overlapping sensitivity and specificity of ELISA and RDT.

Treating HCV infection in early stages results in halting the progression to advanced stages which are associated with high management costs. Our previous analysis reports that, giving treatment based on the current guidelines decreases the cases of DC and HCC developed over lifetime by half as compared to no treatment.[26] Secondly, the effectiveness rates of the DAA based treatment are as

For peer review only - http://bmjopen.bmj.com/site/about/guidelines.xhtml

high as 90%.[49-51] Thirdly, the prices of DAA and diagnostic tests have been significantly reduced through price negotiation with bulk procurement of the drugs and reagents. All these factors support the cost saving nature of the screening and treatment intervention for HCV in Punjab.

Though the intervention of screening appears to be dominant, it is associated with a huge budget impact. Screening the population of 18 years and above, at base coverage of 30%, ELISA costs 1779 million, i.e, 3.8% of the Punjab state health budget[52] whereas with RDT 1586 million rupees, i.e, 3.4% of the state health budget. However, if screening the age cohort of 40-45 years, with ELISA and RDT, account for 0.45% and 0.41% of the state health budget respectively. Therefore, the budget impact might be a guiding factor to decide which subset of the population and at what coverage should the screening be initiated. As the figures suggest, though the intervention is cost saving, but it is not feasible to initiate screening at full coverage and for the whole population. In addition, given the current human resources, who perform other tests as well in addition to HCV, screening the 18 years and older at a coverage of 30%, would require an addition 186,336 manhours (1260 persons). Similarly, screening the age cohort of 40-45 years would require an additional 14,665 manhours (99 persons). However, if one dedicated person to HCV screening is deployed at each of the districts, the human resources will be sufficient to screen the age cohort of 40-45 years whereas, additional 1015 persons would be required to screen everyone 18 years and older.

Our findings are consistent with other studies in the same domain.[53-55] A model-based analysis evaluated the cost-effectiveness as well as budgetary impact of HCV treatment in India which included reinfections and their annual re-testing. The findings of the study demonstrate that treating HCV was highly cost-effective along with annual re-testing for reinfection being cost-effective, thus supporting a policy change towards frequent testing.[53] Another model-based cost effectiveness analysis for screening and treatment of HCV in Egypt also demonstrates one-time screening and treatment to be a dominant strategy as compared to no screening.[54] A study based in the United states (US) compared one-time universal screening as well as birth cohort screening with risk factor

For peer review only - http://bmjopen.bmj.com/site/about/guidelines.xhtml

screening. Both types of interventions were deemed cost effective as compared to risk factor-based screening whereas screening among the general population remained cost-effective under all assumptions.[55]

Studies conducted pertaining to assess the cost-effectiveness of screening in LMIC are very few. Thus, our analysis would contribute to generate evidence pertaining to this area. We used a biologically plausible model which is fitted with local data on disease epidemiology and effectiveness of DAAs which is drawn from the program data of 48,808 HCV patients on treatment in Punjab. Secondly, cost of treatment is drawn from the National Health System Cost Database and nationally representative household surveys for patient costs. Similarly, the cost of screening is also derived from the real-world application in local context. Thirdly, the data on quality of life is also based on our analysis of primary data collected locally. Thus, all the parameters used in the model are estimates from the real-world setting for which the analysis is being conducted.

We do acknowledge certain limitations of the present study. We assumed the coverage estimated for screening to be 30% in the base case as there is no active screening program in operation which could provide us with a realistic estimate. However, we varied the coverage estimate in our PSA from 10 to 50%. Also, we conducted a univariate analysis to report ICER/QALY at different coverage levels for 10 to 100%.

Secondly, high risk group (HRG) screening assessment would have been a better strategy to generate evidence as India is a resource limited setting and prevalence among HRGs is high. However, our results demonstrate that even universal screening among healthy population is cost saving thus it is highly suggestive that screening among HRG would be even more cost saving.[56-59] Thirdly, we did not consider the possibility of reinfection once treated in our model as we were estimating the impact of one-time screening and treatment. Therefore, we assumed that including reinfection would not change the direction of results.

For peer review only - http://bmjopen.bmj.com/site/about/guidelines.xhtml

Fourthly, we did not include productivity losses in our cost analysis. However, as it has also been argued that it is somewhat reflected in the valuation of consequences when trade-off techniques are used to elicit quality of life.[60] Moreover, there are methodological as well as parameter uncertainties which are likely to be introduced when productivity losses are accounted for. Finally, the national HTA guidelines for India have not recommended inclusion of productivity losses.

Conclusion:

Our findings suggest that one-time universal screening followed by treatment of HCV infection is a dominant strategy as compared to no screening. Though, screening is deemed cost saving at all coverage rates but the budget impact of screening program is very high. Thus, based on the ground of both cost-effectiveness and budget impact we recommend screening the age cohort around the mean age of presentation of disease instead of the whole population with RDT instead of ELISA. We cannot deny that screening is important as less than 1% people in India are aware of their HCV status. Due to this, the needy population is unable to access the benefit of available low cost and highly efficacious drug treatment. Reforms should be introduced to improve both screening and treatment activities to cope up with the rising burden of HCV.

For peer review only

For peer review only - http://bmjopen.bmj.com/site/about/guidelines.xhtml

Contributor ship statement:
None

Competing interests:
Authors declare no competing interests.

Funding:
The Health Intervention and Technology Assessment Program (HITAP) provided technical supervision for this study under the auspices of the International Decision Support Initiative (iDSI). HITAP's International Unit is supported by iDSI to provide technical assistance on health intervention and technology assessment to governments in low- and middle-income countries. iDSI is funded by the Bill & Melinda Gates Foundation [OPP1134345], the United Kingdom's Department for International Development, and the Rockefeller Foundation. The findings, interpretations and conclusions expressed in this article do not necessarily reflect the views of the funding agencies.

Data sharing statement:
All the required data pertaining to the analysis has been provided either in the main text or tables and figures.

For peer review only

For peer review only - http://bmjopen.bmj.com/site/about/guidelines.xhtml

496 **References:**

497 1. World Health Organization. Global hepatitis report. Geneva: World Health
498 Organization; 2017. 68 p.
499 2. Hepatitis C [Internet]. Who.int. 2020 [cited 11 February 2020]. Available from:
500 https://www.who.int/en/news-room/fact-sheets/detail/hepatitis-c
501 3. Hepatitis C: simplified curative treatments can drive global scale-up [Internet]. World
502 Health Organization. 2018 [cited 11 February 2020]. Available from:
503 https://www.who.int/hepatitis/news-events/hep-c-scale-up/en/
504 4. World Health Organization. Progress report on access to Hepatitis C treatment: focus
505 on overcoming barriers in low- and middle-income countries. Geneva: World Health
506 Organization; 2018. 51p.
507 5. World Health Organization. Global health sector strategy on viral hepatitis, 2016–
508 2021: Towards ending viral hepatitis. Geneva: World Health Organization; 2016. 53p.
509 6. World Health Organization. Consolidated strategic information guidelines for viral
510 hepatitis: Planning and tracking progress towards elimination. Geneva: World Health
511 Organization; 2019. 28p.
512 7. World Health Organization. Combating hepatitis B and C to reach elimination by 2030.
513 Geneva: World Health Organization; 2016. 17p.
514 8. Government of India. National Action Plan Combating: Viral Hepatitis in India. New
515 Delhi: Ministry of Health and Family Welfare; 2019. 50p.
516 9. Mukhopadhya A. Hepatitis C in India. J Biosci. 2008;33(4):465-73.
517 10. Sievert W, Altraif I, Razavi HA, Abdo A, Ahmed EA, Alomair A, et al. A systematic
518 review of hepatitis C virus epidemiology in Asia, Australia and Egypt. Liver Int.
519 2011;31(SUPPL. 2):61-80.

For peer review only

For peer review only - http://bmjopen.bmj.com/site/about/guidelines.xhtml

11. Puri P, Anand AC, Saraswat VA, Acharya SK, Dhiman RK, Aggarwal R, et al. Consensus statement of HCV task force of the Indian National Association for Study of the Liver (INASL). Part I: Status report of HCV infection in India. J Clin Exp Hepatol.2014;4(2):106-16.

12. Dhiman R, Satsangi S, Grover G, Puri P. Tackling the Hepatitis C Disease Burden in Punjab, India. J Clin Exp Hepatol. 2016;6(3):224-232.

13. Sood A, Suryaprasad A, Trickey A, Kanchi S, Midha1 V, M. Foster MA, et al. The burden of hepatitis C virus infection in Punjab, India: A population-based serosurvey. PLoS ONE 2018:13(7): e0200461.

14. Saraswat V, Norris S, de Knegt R, Sanchez Avila J, Sonderup M, Zuckerman E et al. Historical epidemiology of hepatitis C virus (HCV) in select countries - volume 2. Journal of Viral Hepatitis. 2015;22:6-25.Government of Punjab. Mukh Mantri Punjab Hepatitis-C Relief Fund (MMPHCRF)-One Year Journey. Punjab: Department of Health and Family Welfare, Punjab; 2017. 8 p.

15. Tan-Torres Edejer T, Baltussen R, Adam T, Hutubessy R, Acharya A, Evans D, et al. Making choices in health: WHO guide to cost-effectiveness analysis. Geneva: World Health Organization; 2003.

16. Dhiman R, Grover G, Premkumar M, Taneja S, Duseja A, Arora S et al. Decentralized care with generic direct-acting antivirals in the management of chronic hepatitis C in a public health care setting. Journal of Hepatology. 2019;71(6):1076-1085.

17. Prinja S, Bahuguna P, Faujdar D, Jyani G, Srinivasan R, Ghoshal S et al. Cost-effectiveness of human papillomavirus vaccination for adolescent girls in Punjab state: Implications for India's universal immunization program. Cancer. 2017;123(17):3253-3260.

For peer review only - http://bmjopen.bmj.com/site/about/guidelines.xhtml

544 18. Prinja S, Chauhan A, Angell B, Gupta I, Jan S. A Systematic Review of the State of Economic Evaluation for Health Care in India. Applied Health Economics and Health Policy. 2015;13(6):595-613.

547 19. Department of Health Research. Health Technology Assessment in India. New Delhi : Ministry of Health & Family Welfare, Government of India; 2018.

549 20. World Health Organisation. Viral hepatitis C treatment in India. Available at: http://www.searo.who.int/india/mediacentre/events/2016/viral_hepatitis_c_treatment_in_india.pdf?ua=1 [Accessed on 9 December 2018].

552 21. Gakidou E, Nordhagen S, Obermeyer Z. Coverage of cervical cancer screening in 57 countries: low average levels and large inequalities. PLoS medicine. 2008 Jun;5(6).

554 22. Sharma M, Ying R, Tarr G, Barnabas R. Systematic review and meta-analysis of community and facility-based HIV testing to address linkage to care gaps in sub-Saharan Africa. Nature. 2015 Dec;528(7580):S77-85.

557 23. Coffin PO, Scott JD, Golden MR, Sullivan SD. Cost-effectiveness and population outcomes of general population screening for hepatitis C. Clinical infectious diseases. 2012 May 1;54(9):1259-71.

560 24. Bassett IV, Walensky RP. Integrating HIV screening into routine health care in resource-limited settings. Clinical Infectious Diseases. 2010 May 15;50(Supplement_3):S77-84.

563 25. Tang W, Chen W, Amini A, Boeras D, Falconer J, Kelly H, Peeling R, Varsaneux O, Tucker JD, Easterbrook P. Diagnostic accuracy of tests to detect Hepatitis C antibody: a meta-analysis and review of the literature. BMC infectious diseases. 2017 Nov 1;17(1):695.

567 26. Chugh Y, Dhiman R, Premkumar M, Prinja S, Singh Grover G, Bahuguna P. Real-world cost-effectiveness of pan-genotypic Sofosbuvir-Velpatasvir combination versus

For peer review only - http://bmjopen.bmj.com/site/about/guidelines.xhtml

genotype dependent directly acting anti-viral drugs for treatment of hepatitis C patients in the universal coverage scheme of Punjab state in India. PLOS ONE. 2019;14(8):e0221769.

27. Bedossa P, Poynard T. An algorithm for the grading of activity in chronic hepatitis C. Hepatology.1996;24(2):289-293.

28. Prinja S, Bahuguna P, Duseja A, Kaur M, Chawla YK. Cost of Intensive Care Treatment for Liver Disorders at Tertiary Care Level in India. PharmacoEconomics - Open. 2017:1-12.

29. Prinja S, Sharma D, Aggarwal A, Bahuguna P, Sharma A, Rana S. Out-of-pocket expenditure for hospitalization in Haryana State of India: Extent, determinants & financial risk protection. Indian Journal of Medical Research. 2017;146(6):759.

30. Prinja S, Gupta A, Verma R, Bahuguna P, Kumar D, Kaur M et al. Cost of Delivering Health Care Services in Public Sector Primary and Community Health Centres in North India. PLOS ONE. 2016;11(8):0160986.

31. Kaur G, Prinja S, Malhotra P, Lad D, Prakash G, Khadwal A et al. Cost of Treatment of Multiple Myeloma in a Public Sector Tertiary Care Hospital of North India. 2018.

32. XE: Convert USD/INR. United States Dollar to India Rupee [Internet]. Xe.com. 2020 [cited 16 February 2020]. Available from: https://www.xe.com/currencyconverter/convert/?Amount=1&From=USD&To=INR.

33. National Sample Survey Office.Key Indicators of Social Consumption in India: Health (NSSO 71st Round, January–June 2014). New Delhi: NSSO;2015. 71 p.

34. Khuroo M, Khuroo N, Khuroo M. Diagnostic Accuracy of Point-of-Care Tests for Hepatitis C Virus Infection: A Systematic Review and Meta-Analysis. PLOS ONE. 2015;10(3):e0121450.

For peer review only - http://bmjopen.bmj.com/site/about/guidelines.xhtml

35. Shivkumar S, Peeling R, Jafari Y, Joseph L, Pant Pai N. Accuracy of Rapid and Point-of-Care Screening Tests for Hepatitis C. Annals of Internal Medicine. 2012;157(8):558.

36. Chong CA, Gulamhussein A, Heathcote EJ, Lilly L, Sherman M, Naglie G, et al. Healthstate utilities and quality of life in hepatitis C patients. Am J Gastroenterol. 2003;98:6308.

37. Thein H, Yi Q, Dore G, Krahn M. Estimation of stage-specific fibrosis progression rates in chronic hepatitis C virus infection: A meta-analysis and meta-regression. Hepatology. 2008;48(2):418-431.

38. Van der Meer A, Veldt B, Feld J, Wedemeyer H, Dufour J, Lammert F et al. Association Between Sustained Virological Response and All-Cause Mortality Among Patients With Chronic Hepatitis C and Advanced Hepatic Fibrosis. JAMA. 2012;308(24):2584.

39. Planas R, Ballesté B, Antonio Álvarez M, Rivera M, Montoliu S, Anton Galeras J et al. Natural history of decompensated hepatitis C virus-related cirrhosis. A study of 200 patients. Journal of Hepatology. 2004;40(5):823-830.

40. D'Amico G, Garcia-Tsao G, Pagliaro L. Natural history and prognostic indicators of survival in cirrhosis: A systematic review of 118 studies. Journal of Hepatology. 2006;44(1):217-231.

41. Altekruse S, McGlynn K, Reichman M. Hepatocellular Carcinoma Incidence, Mortality, and Survival Trends in the United States From 1975 to 2005. Journal of Clinical Oncology. 2009;27(9):1485-1491.

42. Registrar General & Census Commissioner of India. SRS life tables 2011-2015. http://www.censusindia.gov.in/Vital_Statistics/SRS_Life_Table/Srs_life_Table_2011-15.html [Accessed 20 August 2018].

For peer review only

For peer review only - http://bmjopen.bmj.com/site/about/guidelines.xhtml

43. Briggs A, Weinstein M, Fenwick E, Karnon J, Sculpher M, Paltiel A. Model Parameter Estimation and Uncertainty: A Report of the ISPOR-SMDM Modeling Good Research Practices Task Force-6. Value in Health. 2012;15(6):835-842.

44. Claxton K. Exploring Uncertainty in Cost-Effectiveness Analysis. PharmacoEconomics. 2008;26(9):781-798.

45. Revill P, Ochalek J, Lomas J, Nakamura R, Woods B, Rollinger A et al. Cost-effectiveness thresholds: guiding health care spending for population health improvement. Centre for Health Economics, University of York; 2015. 24 p.

46. Prinja S, Sundararaman T, Muraleedharan VR. Cost-effectiveness Threshold and Health Opportunity Cost. Economic & Political Weekly. 2020 Jan 11;55(2):19.

47. Punjab Population Sex Ratio in Punjab Literacy rate data 2011-2020 [Internet]. Census2011.co.in. 2020 [cited 19 February 2020]. Available from: https://www.census2011.co.in/census/state/punjab.html

48. Sullivan SD, Mauskopf JA, Annemans L, et al. Principles of good practice for budget impact analysis: report of the ISPOR Task Force on Good Research Practices—Budget Impact Analysis. Value Health. 2007;10(5):336-347.

49. Lee R, Kottilil S, Wilson E. Sofosbuvir/velpatasvir: a pangenotypic drug to simplify HCV therapy. Hepatol Int. 2017;11(2):161-70.

50. Feld JJ, Jacobson IM, Hézode C, Asselah T, Ruane PJ, Gruener N, et al. Sofosbuvir and Velpatasvir for HCV Genotype 1, 2, 4, 5, and 6 Infection. N Engl J Med. 2015;373(27):2599-607.

51. Nelson DR, Cooper JN, Lalezari JP, et al. All-oral 12-week treatment with daclatasvir plus sofosbuvir in patients with hepatitis C virus genotype 3 infection: ALLY-3 phase 3 study. Hepatology. 2015;61:1127-1135.

For peer review only - http://bmjopen.bmj.com/site/about/guidelines.xhtml

52. Ministry of Health and family welfare, Government of Punjab. Budget 2020-21. Punjab: Government of Punjab; 2020 p. 73.

53. Chaillon A, Mehta SR, Hoenigl M, Solomon SS, Vickerman P, Hickman M, Skaathun B, Martin NK. Cost-effectiveness and budgetary impact of HCV treatment with direct-acting antivirals in India including the risk of reinfection. PloS one. 2019;14(6).

54. Kim DD, Hutton DW, Raouf AA, Salama M, Hablas A, Seifeldin IA, Soliman AS. Cost-effectiveness model for hepatitis C screening and treatment: implications for Egypt and other countries with high prevalence. Global public health. 2015 Mar 16;10(3):296-317.

55. Coffin PO, Scott JD, Golden MR, Sullivan SD. Cost-effectiveness and population outcomes of general population screening for hepatitis C. Clinical infectious diseases. 2012 May 1;54(9):1259-71.

56. Castelnuovo E, Thompson-Coon J, Pitt M, Cramp M, Siebert U, Price A, Stein K. The cost-effectiveness of testing for hepatitis C in former injecting drug users. InNIHR Health Technology Assessment programme: Executive Summaries 2006. NIHR Journals Library.

57. Thompson Coon J, Castelnuovo E, Pitt M, Cramp M, Siebert U, Stein K. Case finding for hepatitis C in primary care: a cost utility analysis. Family practice. 2006 Aug 1;23(4):393-406.

58. Tramarin A, Gennaro N, Compostella FA, Gallo C, Wendelaar Bonga LJ, Postma MJ. HCV Screening to Enable Early Treatment of Hepatitis C: A Mathematical Model to Analyse Costs and Outcomes in Two Populations. Current pharmaceutical design. 2008 Jun 1;14(17):1655-60.

For peer review only - http://bmjopen.bmj.com/site/about/guidelines.xhtml

59. Helsper CW, Borkent-Raven BA, De Wit NJ, Van Essen GA, Bonten MJ, Hoepelman AI, Janssen MP, De Wit GA. Cost-effectiveness of targeted screening for hepatitis C in The Netherlands. Epidemiology & Infection. 2012 Jan;140(1):58-69.

60. Pike J, Grosse S. Friction Cost Estimates of Productivity Costs in Cost-of-Illness Studies in Comparison with Human Capital Estimates: A Review. Applied Health Economics and Health Policy. 2018;16(6):765-778.

61. Government of India. National guidelines for Diagnosis & Management of Viral Hepatitis. New Delhi: Ministry of Health and Family Welfare; 2018. 78p.

For peer review only

For peer review only - http://bmjopen.bmj.com/site/about/guidelines.xhtml

Tables

Table 1: Demographic, epidemiologic, effectiveness and cost-related parameters.

Parameters	Base value	Lower limit	Upper limit	Distribution	Source
Epidemiological parameters					
Viraemic prevalence of HCV in Punjab	0.026	0.0234	0.0286	Uniform	12-14
19-29 years	0.012	0.005	0.019	Uniform	12-14
30-39 years	0.031	0.018	0.043	Uniform	12-14
40-49 years	0.047	0.031	0.062	Uniform	12-14
50-59 years	0.045	0.027	0.062	Uniform	12-14
>/=60 years	0.027	0.014	0.039	Uniform	12-14
Stage-wise distribution of HCV at diagnosis					
F0	0.325	0.30875	0.34125	Uniform	16,26
F1	0.325	0.30875	0.34125	Uniform	16,26
F2	0.1	0.095	0.105	Uniform	16,26
F3	0.1	0.095	0.105	Uniform	16,26
F4	0.12	0.114	0.126	Uniform	16,26
DC	0.03	0.0285	0.0315	Uniform	16,26
Proportion of population that is RBV tolerant	0.9	0.81	0.99	Uniform	16,26
Effectiveness parameters					
Quality of life weights					

For peer review only

For peer review only - http://bmjopen.bmj.com/site/about/guidelines.xhtml

F0 - F3	0.63	0.57	0.70	Beta	26
F4	0.56	0.51	0.61	Beta	26
DC	0.44	0.38	0.49	Beta	26
HCC	0.44	0.38	0.49	Beta	26
F0-F3 (post SVR)	1	0.83	1	Beta	26,36
Discount rate	0.03	0.02	0.08	Uniform	17-20
Transition probabilities					
F0 to F1	0.177	0.104	0.13	Beta	38,39
F1 to F2	0.085	0.075	0.096	Beta	38,39
F2 to F3	0.12	0.109	0.133	Beta	38,39
F3 to F4	0.116	0.104	0.129	Beta	38,39
F4 to DC	0.035	0.027	0.043	Beta	38,39
F4 to DC (post SVR)	0.002	0.0001	0.005	Beta	38,39
F4 to HCC	0.024	0.018	0.031	Beta	38,39
F4 to HCC (post SVR)	0.005	0.001	0.009	Beta	38,39
DC to HCC	0.068	0.03	0.083	Beta	38,39
DC to HCC (post SVR)	0.03	0.0225	0.0375	Beta	38,39
Probability of dying due to HCV					
F0 – F4	0	0	0	Uniform	26
DC	0.216	0.162	0.27	Uniform	40-42
HCC	0.411	0.31	0.51	Uniform	40-42
SVR rates (%)					

For peer review only - http://bmjopen.bmj.com/site/about/guidelines.xhtml

SOF+DCV (12 weeks)	74	66.5	73.5	Uniform	16,26
SOF+VEL (12 weeks)	84	79.8	88.2	Uniform	16,26
SOF+VEL (24 weeks)	81	76.95	85.05	Uniform	16,26
SOF+VEL+RBV (12 weeks)	84	79.8	88.2	Uniform	16,26
Sensitivity ELISA	0.952	0.918	0.972	Beta	34,35
Specificity ELISA	0.986	0.976	0.993	Beta	34,35
Sensitivity RDT	0.984	0.889	0.998	Beta	34,35
Specificity RDT	0.986	0.949	0.996	Beta	34,35
Coverage parameters					
Treatment coverage in do-nothing scenario (%)	30	20	50	Uniform	Author's assumption
Coverage of screening (%)	5	0	10	Uniform	Author's assumption
Loss to follow up at each step (%)	10	5	20	Uniform	Author's assumption
Cost parameters					
Drug costs-12 weeks (□)					
SOF+DCV	4509	3607.2	4509	Gamma	15
SOF+VEL	13104	10483.2	13104	Gamma	15
SOF+VEL+RBV	16818	13454.4	16818	Gamma	15
Cost of diagnostic tests (□)					
ELISA	72.95	58.36	109.425	Gamma	15

For peer review only

RDT	39	31.2	58.5		15
HCV-RNA	880	704	1320	Gamma	15
Routine tests (CBC, LFT, Creatinine)	500	400	750	Gamma	15
Cirrhosis evaluation (Fibro-scan)	350	280	525	Gamma	15
Genotyping	895	716	1342.5	Gamma	15
Cost per OPD consultation (□)					
Primary	1686.3	1180.41	2192.19	Gamma	28-31
Secondary	1734	1213.8	2254.2	Gamma	28-31
Tertiary	2024	1416.8	2631.2	Gamma	28-31
Cost per patient hospitalisation (□)					
Primary	6347.1	4442.97	8251.23	Gamma	28-31
Secondary	7597	5317.9	9876.1	Gamma	28-31
Tertiary	18693	13085.1	24300.9	Gamma	28-31
Per unit cost of integrated training (□)					
District	50000	40000	75000	Gamma	Expert opinion
State	308000	246400	462000	Gamma	Expert opinion

For peer review only

For peer review only - http://bmjopen.bmj.com/site/about/guidelines.xhtml

Per district cost of IEC/BCC (□)	4000	3200	6000	Gamma	Expert opinion

HCV-Hepatitis C virus, DC-Decompensated cirrhosis, HCC-Hepatocellular carcinoma, SOF-Sofosbuvir, LDV-Ledipasvir, RBV-Ribavirin, DCV-Daclatasvir, VEL-Velpatasvir, GT-genotype, OPD- Out-patient Department, IPD- In-patient Department, ELISA: Enzyme Linked Immunosorbent Assay, RDT: Rapid Diagnostic Tests, IEC/BCC: Information-education-counselling and Behaviour change communication, □: Indian rupee.

For peer review only - http://bmjopen.bmj.com/site/about/guidelines.xhtml

Table 2: Cost and Effects of Screening and treating Chronic hepatitis C virus infected Patients

**No-Screening**	
Life years per patient	15.75 (11.79-23.85)
QALY per patient	15.57 (11.63-23.60)
Cost per patient (□)	2,775 (1,549-4,898)
**Scenario I**	
Life years per patient	15.92 (11.79-23.86)
QALY per patient	15.78 (11.65-23.67)
Cost per patient (□)	998 (456-1,932)
**Scenario II**	
Life years per patient	15.75 (11.79-23.86)
QALY per patient	15.60 (11.65-23.63)
Cost per patient (□)	947 (468-1,837)
**Incremental gains**	
Scenario I- No screening	
Life years gained	0.0046 (0.0013-0.011)
QALYs gained	0.028 (0.008-0.061)
Cost difference (□)	-1,810 (-3,376 to – 867)
Scenario II- No screening	
Life years gained	0.0045 (0.001-0.012)
QALYs gained	0.027 (0.008-0.061)
Cost difference (□)	-1,812 (-3,468 to -850)

HCV: Hepatitis C virus, QALY: Quality adjusted life year, □: Indian rupee

For peer review only - http://bmjopen.bmj.com/site/about/guidelines.xhtml

Table 3 A: Budget Impact Assessment: Estimates spread across the time horizon (year 1 to year 5) in million rupees (million US $)

		ELISA	RDT
**18 Years and older**	Year 1	1779.42 (25.24)	1586.32 (22.5)
	Year 2	52.81 (0.75)	47.1 (0.67)
	Year 3	53.05 (0.75)	47.54 (0.67)
	Year 4	53.44 (0.76)	48.27 (0.68)
	Year 5	53.93 (0.76)	49.16 (0.7)
**40-45 Years**	Year 1	208.7 (2.96)	190.46 (2.7)
	Year 2	45.83 (0.65)	41.83 (0.59)
	Year 3	46.07 (0.65)	42.07 (0.6)
	Year 4	46.46 (0.66)	42.46 (0.6)
	Year 5	46.94 (0.67)	42.94 (0.61)

ELISA: Enzyme Linked Immunosorbent Assay, RDT: Rapid Diagnostic Tests, US $: United States Dollar.

For peer review only

For peer review only - http://bmjopen.bmj.com/site/about/guidelines.xhtml

Table 3 B: Budget Impact Assessment: Resource wise disaggregated estimates of budget for the 1st year at base coverage (30%) in million rupees (million US $)

	40-45 Years		18 Years and older	
	**ELISA**	**RDT**	**ELISA**	**RDT**
**HR**	1.314 (0.019)	1.314 (0.019)	3.367 (0.048)	3.367 (0.048)
**IEC/BCC**	0.088 (0.001)	0.088 (0.001)	0.088 (0.001)	0.088 (0.001)
**Screening Test**	35.94 (0.51)	17.7 (0.251)	380.39 (5.396)	187.29 (2.657)
**Confirmatory Test**	22.12 (0.314)	22.12 (0.314)	180.16 (2.555)	180.16 (2.555)
**Drugs**	149.24 (2.117)	149.24 (2.117)	1215.42 (17.24)	1215.42 (17.24)
**Total**	208.702 (2.96)	190.462 (2.702)	1779.425 (25.24)	1586.325 (22.501)

HR: Human resources, IEC/BCC: Information-Education-Counselling-Behaviour-Change-Communication, ELISA: Enzyme Linked Immunosorbent Assay, RDT: Rapid Diagnostic Tests, US $: United States Dollar.

For peer review only - http://bmjopen.bmj.com/site/about/guidelines.xhtml

Table 3 C: Budget Impact Assessment: Estimates for different screening scenarios in million rupees (million US $)

Coverage of screening (%)	18 Years and older		Age cohort 40-45 years	
	**ELISA**	**RDT**	**ELISA**	**RDT**
10	593.14 (8.41)	528.78 (7.5)	69.57 (0.99)	63.49 (0.9)
20	1186.28 (16.83)	1057.54 (15)	139.13 (1.97)	126.97 (1.8)
30	1779.42 (25.24)	1586.32 (22.5)	208.7 (2.96)	190.46 (2.7)
50	2965.69 (42.07)	2643.87 (37.5)	347.84 (4.93)	317.44 (4.5)
70	4151.97 (58.89)	3701.41 (52.5)	486.98 (6.91)	444.42 (6.3)
100	5931.39 (84.13)	5287.73 (75)	695.68 (9.87)	634.88 (9.01)

ELISA: Enzyme Linked Immunosorbent Assay, RDT: Rapid Diagnostic Tests, US $: United States Dollar.

For peer review only

755 **Figures**

756 Figure 1: Treatment guidelines for management of Chronic Hepatitis C virus infection.[61]

757 Figure 2 A: Model structure: Decision tree depicting the screening pathway for different scenarios.

758 Figure 2 B: Model structure: Markov model depicting the natural history of Hepatitis C infection.

759 Figure 3: Cost-effectiveness planes for scenario I and II vs No screening.

760

761

762

763

For peer review only

For peer review only - http://bmjopen.bmj.com/site/about/guidelines.xhtml

Figure 1: Treatment guidelines for management of Chronic Hepatitis C virus infection.[61]

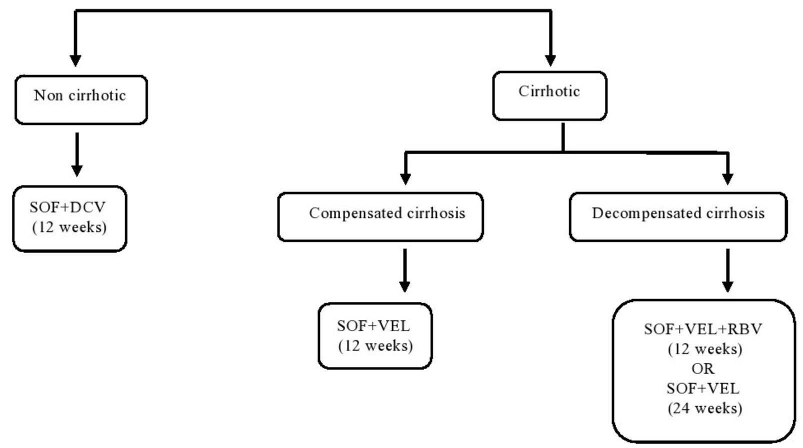

SOF-Sofosbuvir, LDV-Ledipasvir, RBV-Ribavirin, DCV-Daclatasvir, VEL-Velpatasvir

Figure 1: Treatment guidelines for management of Chronic Hepatitis C virus infection

321x294mm (96 x 96 DPI)

Figure 2 A: Model structure: Decision tree depicting the screening pathway for different scenarios.

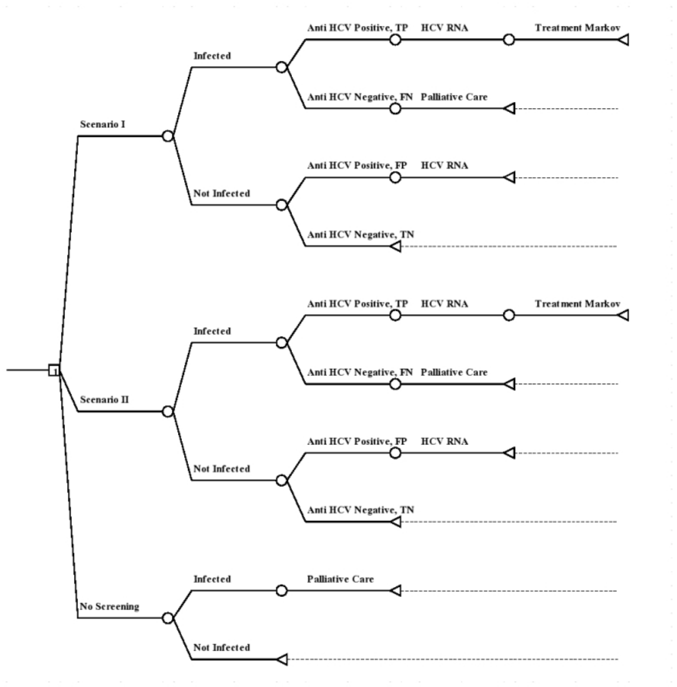

Scenario I: Screening with Enzyme Linked Immunosorbent Assay, Scenario II: Screening with Rapid Diagnostic Tests, HCV: Hepatitis C virus, RNA: Ribo-nucleic Acid, TP: True Positive, TN: True Negative, FN: False Negative, FP: False Positive

Figure 2 A: Model structure: Decision tree depicting the screening pathway for different scenarios

437x618mm (72 x 72 DPI)

Figure 2 B: Model structure: Markov model depicting the natural history of Hepatitis C infection.

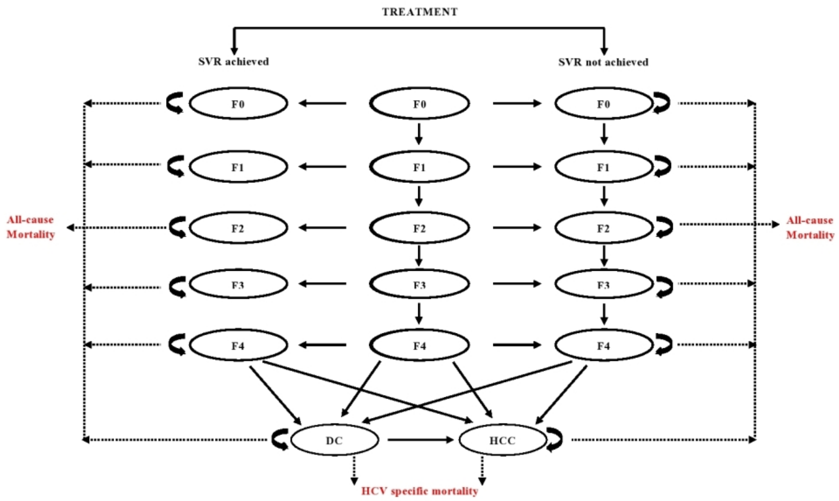

SVR: Sustained Virologic Response, F0: No fibrosis, F: Portal fibrosis without septa, F2: Portal fibrosis with few septa, F3: Numerous septa without cirrhosis, F4: Cirrhosis, DC: Decompensated cirrhosis, HCC: Hepatocellular carcinoma

Figure 2 B: Model structure: Markov model depicting the natural history of Hepatitis C infection

618x437mm (72 x 72 DPI)

Figure 3: Cost-effectiveness planes for scenario I and II vs No screening:

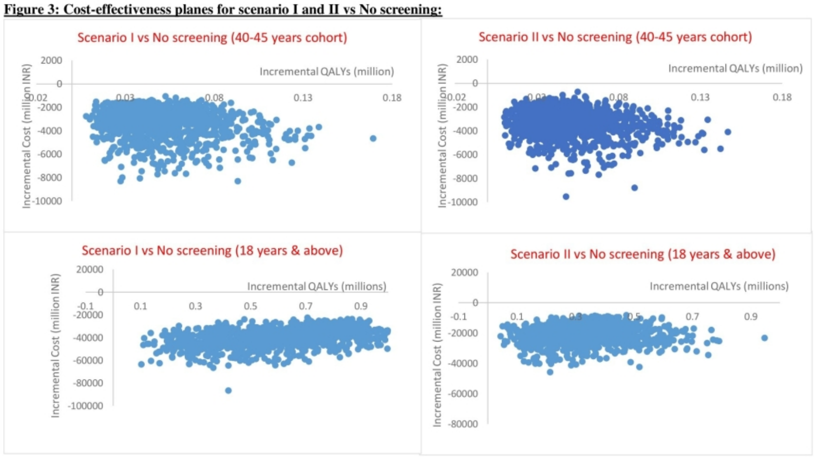

QALY: Quality Adjusted Life Years, INR: Indian rupee

Figure 3: Cost-effectiveness planes for scenario I and II vs No screening

59410x42011mm (1 x 1 DPI)

CHEERS checklist: Cost-effectiveness and budget impact analysis of screening and treating Hepatitis C infected population vs no screening and treatment in Punjab state of India

Section/item	Item No	Recommendation	Reported on page No/ line No
**Title and abstract**			
Title	1	Identify the study as an economic evaluation or use more specific terms such as "cost-effectiveness analysis", and describe the interventions compared.	Page 1, Line 1-2
Abstract	2	Provide a structured summary of objectives, perspective, setting, methods (including study design and inputs), results (including base case and uncertainty analyses), and conclusions.	Page 3, Line 83-104
**Introduction**			
Background and objectives	3	Provide an explicit statement of the broader context for the study.	Page 5, Line 143-151
		Present the study question and its relevance for health policy or practice decisions.	Page 6, Line 164-170
**Methods**			
Target population and subgroups	4	Describe characteristics of the base case population and subgroups analysed, including why they were chosen.	Page 5, Line 146-149; Page 9, Line 227-233
Setting and location	5	State relevant aspects of the system(s) in which the decision(s) need(s) to be made.	Page 5, Line 146-149, 152-155; Page 6, Line 159-163
Study perspective	6	Describe the perspective of the study and relate this to the costs being evaluated.	Page 9, Line 244-149; Page 10, Line 261-264
Comparators	7	Describe the interventions or strategies being compared and state why they were chosen.	Page 7, Line 190-203; Page 8, Line 204-217
Time horizon	8	State the time horizon(s) over which costs and consequences are being evaluated and say why appropriate.	Page 7, Line 183-184
Discount rate	9	Report the choice of discount rate(s) used for costs and outcomes and say why appropriate.	Page 7, Line 185-189
Choice of health outcomes	10	Describe what outcomes were used as the measure(s) of benefit in the evaluation and their relevance for the type of analysis performed.	Page 7, Line 184-185, 187-189
Measurement of effectiveness	11a	*Single study-based estimates:* Describe fully the design features of the single effectiveness study and why the single study was a sufficient source of clinical effectiveness data.	Page 10, Line 267-270; Page 11, Line 278-279; Page 17, Line 419-422
	11b	*Synthesis-based estimates*: Describe fully the methods used for identification of included studies and synthesis of clinical effectiveness data.	
Measurement and valuation of preference-based outcomes	12	If applicable, describe the population and methods used to elicit preferences for outcomes.	Page 10, Line 270-274
Estimating resources and costs	13a	*Single study-based economic evaluation:* Describe approaches used to estimate resource use associated with the alternative interventions. Describe primary or secondary research methods for valuing each resource item in terms of its unit cost. Describe any adjustments made to approximate to opportunity costs.	Page 9, Line 245-250; Page 10, Line 251-265
	13b	*Model-based economic evaluation:* Describe approaches and data sources used to estimate resource use associated with model health states. Describe primary or secondary research methods for valuing each resource item in terms of its unit cost. Describe any adjustments made to approximate to opportunity costs.	
Currency, price date, and conversion	14	Report the dates of the estimated resource quantities and unit costs. Describe methods for adjusting	Page 10, Line 260-261

For peer review only

Section/item	Item No	Recommendation	Reported on page No/ line No
		estimated unit costs to the year of reported costs if necessary. Describe methods for converting costs into a common currency base and the exchange rate.	
Choice of model	15	Describe and give reasons for the specific type of decision-analytical model used. Providing a figure to show model structure is strongly recommended.	Page 8, Line 219-220; Figure 2 (A & B)
Assumptions	16	Describe all structural or other assumptions underpinning the decision-analytical model.	Page 7, Line 194-196, 203; Page8, Line 205; Page 11, Line 284-287
Analytical methods	17	Describe all analytical methods supporting the evaluation. This could include methods for dealing with skewed, missing, or censored data; extrapolation methods; methods for pooling data; approaches to validate or make adjustments (such as half cycle corrections) to a model; and methods for handling population heterogeneity and uncertainty.	Page 11, Line 290-297; Page 12, Line 298-321; Page 13, Line 322-340
**Results**			
Study parameters	18	Report the values, ranges, references, and, if used, probability distributions for all parameters. Report reasons or sources for distributions used to represent uncertainty where appropriate. Providing a table to show the input values is strongly recommended.	Table 1
Incremental costs and outcomes	19	For each intervention, report mean values for the main categories of estimated costs and outcomes of interest, as well as mean differences between the comparator groups. If applicable, report incremental cost-effectiveness ratios.	Table 2
Characterising uncertainty	20a	*Single study-based economic evaluation:* Describe the effects of sampling uncertainty for the estimated incremental cost and incremental effectiveness parameters, together with the impact of methodological assumptions (such as discount rate, study perspective).	Page 14, Line 363-368; Figure 3
	20b	*Model-based economic evaluation:* Describe the effects on the results of uncertainty for all input parameters, and uncertainty related to the structure of the model and assumptions.	
Characterising heterogeneity	21	If applicable, report differences in costs, outcomes, or cost-effectiveness that can be explained by variations between subgroups of patients with different baseline characteristics or other observed variability in effects that are not reducible by more information.	Page 14, Line 363-368; Figure 3; Table 3 (A, B & C)
**Discussion**			
Study findings, limitations, generalisability, and current knowledge	22	Summarise key study findings and describe how they support the conclusions reached. Discuss limitations and the generalisability of the findings and how the findings fit with current knowledge.	Page 15, Line 381-385, 387-392; Page 16, Line 393-394, 408-417; Page 17, Line 418-441; Page 18, Line 442-443
**7Other**			
Source of funding	23	Describe how the study was funded and the role of the funder in the identification, design, conduct, and reporting of the analysis. Describe other non-monetary sources of support.	Page 1, Line 30-38
Conflicts of interest	24	Describe any potential for conflict of interest of study contributors in accordance with journal policy. In the absence of a journal policy, we recommend authors comply with International Committee of Medical Journal Editors recommendations.	Page 1, Line 40-41

For peer review only

BMJ Open

Cost-effectiveness and budget impact analysis of facility-based screening and treatment of Hepatitis C in Punjab state of India.

Journal:	*BMJ Open*
Manuscript ID	bmjopen-2020-042280.R1
Article Type:	Original research
Date Submitted by the Author:	29-Oct-2020
Complete List of Authors:	Chugh, Yashika; Post Graduate Institute of Medical Education and Research School of Public Health, Department of Community Medicine & School of Public Health<br>Premkumar, Madhumita; Post Graduate Institute of Medical Education and Research, Department of Hepatology<br>Grover, Gagandeep<br>Dhiman, Radha K.; Sanjay Gandhi Post Graduate Institute of Medical Sciences<br>Teerawattananon, Yot; HITAP<br>Prinja, Shankar; Post Graduate Institute of Medical Education and Research School of Public Health, Department of Community Medicine & School of Public Health
**Primary Subject Heading**:	Health economics
Secondary Subject Heading:	Infectious diseases, Health policy, Public health
Keywords:	HEALTH ECONOMICS, Public health < INFECTIOUS DISEASES, Health policy < HEALTH SERVICES ADMINISTRATION & MANAGEMENT

SCHOLARONE™
Manuscripts

I, the Submitting Author has the right to grant and does grant on behalf of all authors of the Work (as defined in the below author licence), an exclusive licence and/or a non-exclusive licence for contributions from authors who are: i) UK Crown employees; ii) where BMJ has agreed a CC-BY licence shall apply, and/or iii) in accordance with the terms applicable for US Federal Government officers or employees acting as part of their official duties; on a worldwide, perpetual, irrevocable, royalty-free basis to BMJ Publishing Group Ltd ("BMJ") its licensees and where the relevant Journal is co-owned by BMJ to the co-owners of the Journal, to publish the Work in this journal and any other BMJ products and to exploit all rights, as set out in our licence.

The Submitting Author accepts and understands that any supply made under these terms is made by BMJ to the Submitting Author unless you are acting as an employee on behalf of your employer or a postgraduate student of an affiliated institution which is paying any applicable article publishing charge ("APC") for Open Access articles. Where the Submitting Author wishes to make the Work available on an Open Access basis (and intends to pay the relevant APC), the terms of reuse of such Open Access shall be governed by a Creative Commons licence – details of these licences and which Creative Commons licence will apply to this Work are set out in our licence referred to above.

Other than as permitted in any relevant BMJ Author's Self Archiving Policies, I confirm this Work has not been accepted for publication elsewhere, is not being considered for publication elsewhere and does not duplicate material already published. I confirm all authors consent to publication of this Work and authorise the granting of this licence.

For peer review only

Title: Cost-effectiveness and budget impact analysis of facility-based screening and treatment of Hepatitis C in Punjab state of India.

Authors: Yashika Chugh[1], Madhumita Premkumar[2], Gagandeep Singh Grover[3], Radha Krishan Dhiman[4,5], Yot Teerawattananon[6], Shankar Prinja[1]

Institution:

1. Department of Community Medicine and School of Public Health, Post Graduate Institute of Medical Education and Research, Chandigarh, India
2. Department of Hepatology, Post Graduate Institute of Medical Education and Research, Chandigarh, India
3. State Program Officer, Hepatitis C Virus Infection, Directorate of Health and Family Welfare, Punjab, India
4. Director, Sanjay Gandhi Postgraduate Institute of Medical Sciences, Lucknow, Uttar Pradesh, India.
5. Chairman, Technical Resource Group - National Viral Hepatitis Control Program (NVHCP), Government of India, India
6. Health Intervention and Technology Assessment Program (HITAP), Ministry of Public Health, Nonthaburi, Thailand & Saw Swee Hock School of Public Health, National University of Singapore.

Short title:
Cost-Effectiveness of universal screening for hepatitis C.

Key words:
cost effectiveness analysis, hepatitis C virus, universal screening, ELISA, RDT, budget impact.

Manuscript details:
359 words abstract, 4708 words main text, 3 tables, 4 figures, 62 references.

Funding:
The Health Intervention and Technology Assessment Program (HITAP) provided technical supervision for this study under the auspices of the International Decision Support Initiative (iDSI). HITAP is funded by the Thailand Research Fund (TRF) under a grant for a Senior Research Scholar (RTA5980011). HITAP is supported by the International Decision Support Initiative (iDSI) to provide technical assistance on health intervention and technology assessment to governments in low- and middle-income countries. iDSI is funded by the Bill & Melinda Gates Foundation (OPP1134345), the UK's Department for International Development, and the Rockefeller Foundation. The findings, interpretations and conclusions expressed in this article do not necessarily reflect the views of the funding agencies.

Conflicts of interest:
No conflicts of interest declared

Ethical Approval:
The present study is a model-based analysis which did not involve any human subjects as well as primary data collection. Therefore, ethical approval was not required. Administrative approval was sought from Department of Health & Family Welfare, Punjab, India.

For peer review only - http://bmjopen.bmj.com/site/about/guidelines.xhtml

Corresponding author:
Dr. Shankar Prinja
Additional Professor of Health Economics
Department of Community Medicine and School of Public Health, Post Graduate Institute of Medical Education and Research
Sector-12, Chandigarh – 160012
Ph: +91 9872871978
Email: shankarprinja@gmail.com

For peer review only - http://bmjopen.bmj.com/site/about/guidelines.xhtml

Cost-effectiveness and budget impact analysis of facility-based screening and treatment of Hepatitis C in Punjab state of India.

Abstract

Objective: Despite treatment availability, chronic Hepatitis C Virus (HCV) public health burden is rising in India due to lack of timely diagnosis. Therefore, we aim to assess incremental cost per quality adjusted life year (QALY) for one-time universal screening followed by treatment of people infected with HCV as compared to a no-screening policy in Punjab, India.

Study design: Decision tree integrated with Markov model was developed to simulate disease progression. A societal perspective and a 3% annual discount rate were considered to assess incremental cost per QALY gained. In addition, budgetary impact was also assessed with a payer's perspective and time horizon of 5 years.

Study setting: Screening services were assumed to be delivered as a facility-based intervention wherein active screening for HCV cases would be performed at 22 district hospitals in the state of Punjab which will act as integrated testing as well as treatment sites for HCV**.**

Intervention: Two intervention scenarios were compared with no universal screening and treatment (routine care). Scenario I – screening with Enzyme Linked Immunosorbent Assay (ELISA) followed by confirmatory HCV-Ribonucleic Acid (RNA) quantification and treatment. Scenario II – screening with Rapid diagnostic test (RDT) kit followed by confirmatory HCV-RNA quantification and treatment.

Primary and secondary outcome measures: Lifetime costs; life years and QALY gained; and Incremental cost-effectiveness ratio (ICER) for each of the above mentioned intervention scenario as compared to the routine scenario.

Results: Screening with ELISA and RDT, respectively, results in a gain of 0.028 (0.008-0.06) and 0.027 (0.008-0.061) QALY per person with costs decreased by □-1,810 (-3,376 to – 867) and □-1,812 (-3,468 to -850) when compared with no-screening. One-time universal screening of all those ≥18 years at a base coverage of 30%, with ELISA and RDT, would cost 8.5 and 8.3 times more, respectively, when compared to screening the age-group of 40-45 year-old cohort.

Conclusion: One-time universal screening followed by HCV treatment is a dominant strategy as compared to no-screening. However, budget impact of screening activity seems unsustainable. Thus, in view of findings from both cost-effectiveness and budget impact, we recommend beginning with screening the age cohort with RDT around mean age of disease presentation, i.e, 40-45 years, instead of whole population.

For peer review only

Article Summary: Strengths and limitations of this study

Strengths

- Our analysis is the first state-level study in India where the country population contributes to around 7.5% of the global disease prevalence. Thus, our results would be applicable to other Indian states with high HCV burden.
- We used a biologically plausible model which is fitted with local data on disease epidemiology and effectiveness of DAAs, which is drawn from the program data of 48,808 persons with chronic HCV infection, who were successfully treated with a cure rate of 91.2% in Punjab, India.
- In addition, cost of treatment is drawn from the National Health System Cost Database and nationally representative household surveys for patient costs.

Limitations

- We did not consider the possibility of reinfection once treated in our model as we were estimating the impact of one-time screening and treatment among general population, not the high-risk groups. Therefore, we assumed that reinfection would be minimal and its inclusion would not change the direction of results.
- We also acknowledge that the sample sizes for estimating the utility values may not be large enough for different health states. However, this is likely to influence the standard errors; the effect of which is likely to be captured in our probabilistic sensitivity analysis (PSA).

For peer review only - http://bmjopen.bmj.com/site/about/guidelines.xhtml

Introduction:

Nearly 71 million people are living with Hepatitis C virus (HCV) infection round the globe.[1,2] HCV infection led to around 399,000 deaths mainly caused by cirrhosis and hepatocellular carcinoma (HCC).[2] With the advent of all-oral Directly Acting Antiviral agents (DAAs), it is possible to cure 90% of the people infected with HCV, however, the awareness of infection, access to diagnostics and treatment facilities is low.[2,3] Globally, only 1 in 5 people living with HCV infection had been diagnosed.[4] Further, amongst those infected with chronic HCV, only 1% of them have accessed the newer antivirals based treatment.[5]

The World Health Assembly (WHA) released the global targets to eliminate viral hepatitis by 2030.[6] The aim is to diagnose 90% of the Hepatitis B virus (HBV) and HCV infections and treat 80% of the eligible infections by 2030.[6,7] With around 40% of the HCV infected people residing in low and middle income countries (LMICs), India constitutes the 4th highest number of viremic HCV population, where only less than 1% of people are aware of their infection status.[4,8,9]

Though the national prevalence of chronic HCV infection in India ranges from 0.9-1.9%,[10,11] it is as high as 3.6% in the northern state of Punjab.[12,13] The high prevalence in Punjab can be attributed to the higher prevalence of unsafe medical procedures and blood transfusions. Another contributor to high HCV burden in Punjab is the Intravenous drug user (IDU) population, which is at a higher risk for reinfection as well.[12] However, it is difficult to identify such people due to associated stigma.

In 2016, Punjab government launched the "Mukh Mantri Punjab Hepatitis C Relief Fund (MMPHCRF)" for free of charge universal treatment of HCV patients with all oral DAAs.[14] Despite free treatment, due to lack of awareness of disease status, less 10% of the total estimated cases in Punjab have been put on treatment.[15] Thus, along with implementation of treatment

For peer review only - http://bmjopen.bmj.com/site/about/guidelines.xhtml

strategies there is a need to focus on screening, diagnosing those with HCV infection and population education strategies to break the chain of transmission.

With the success of the Punjab Model, where a cure rate of more than 90% was achieved among those enrolled, and in concurrence with the global effort to eliminate HCV infection, India launched the National Viral Hepatitis Control Programme (NVHCP) in July 2018 which aims at improving access to diagnosis and treatment for viral hepatitis.[16] This program covers the entire gamut of acute and chronic viral hepatitis including types A, B, C, D & E with a combined strategy of prevention, immunization for HBV, screening, detection and timely treatment.

In view of these recent development to tackle the HCV burden, the Punjab state government plans to move forward with screening in a phased manner. Screening could be done among high risk groups (HRG) or a universal screening strategy could be one options as identification of HRGs which include patients who are frequently recipients of blood products(e.g. thalassemia patients), persons who inject drugs (PWID), patients on haemodialysis, organ transplant recipients and people living with Human Immunodeficiency virus (PLHIV). However, targeting of sensitive groups, such as, PWID and PLHIV can be associated with stigma which may be accompanied by other political as well as practical challenges. Another viable strategy could be screening around the age of presentation of the infection which is around 40 years from the data base of the 48,088 patients treated under MMPHCRF.[15]

To inform this policy decision on the implementation of a screening and treatment program in the state of Punjab, we undertook this analysis to assess the incremental cost per quality adjusted life year (QALY) for one-time universal screening followed by treatment of HCV infected population as compared to no screening in Punjab. This study also aims to inform on which screening technology represents value for money and would yield a better operational feasibility given the fiscal space for funding the HCV screening program. Thus, a budget impact analysis was

For peer review only - http://bmjopen.bmj.com/site/about/guidelines.xhtml

performed in addition to estimate the financial resources required in terms of initiating a screening program in the state.

Methods:

Overview of Analysis

Screening services were assumed to be delivered as a facility-based intervention wherein active screening for HCV cases would be performed at 22 district hospitals in the state of Punjab, via existing resources, which will act as integrated testing as well as treatment sites for HCV. Since, screening is to be initiated in the state, this assumption was made in consultation with experts from "*National Taskforce to Combat Viral Hepatitis*". We modelled the lifetime costs and outcomes for screening and treating HCV infected patients in Punjab considering 3 different scenarios – no universal screening and treatment of those detected in 'routine care' setting, scenario I – screening with Enzyme Linked Immunosorbent Assay (ELISA) followed by confirmatory HCV-Ribonucleic Acid (RNA) quantification, and scenario II – screening with Rapid diagnostic test (RDT) kit followed by confirmatory HCV-RNA quantification. A lifelong time horizon was considered so as to account for all costs and consequences of the intervention for chronic HCV. The outcomes are valued in terms of the number of HCV deaths, life years and QALYs. All the future costs and consequences, i.e, early vs late detection of HCV, new HCV infections averted and HCV deaths averted, are discounted at a rate of 3%. This choice of discounting rate is based on standard international guidelines along with being consistent with other Indian economic evaluations.[17-20] The methodological principles are in concurrence with the Indian reference case for conducting economic evaluations published by the health technology agency in India.[20]

No screening and treatment/ do-nothing/ Routine-care scenario

For peer review only - http://bmjopen.bmj.com/site/about/guidelines.xhtml

The routine care scenario represents the current situation where-in there is no screening program. In this situation, we assume that patients present to the facility of their own volition when symptoms develop and access the required management support. Since the treatment coverage in India is low[1,21], we also assume that only 5% of the HCV infected cohort receives the testing and treatment on their own and the rest progress to advance stages of HCV as per the natural progression of the disease. The drug regimen followed to treat those infected is as per the national guidelines for diagnosis and management of HCV in India (Figure 1). The rest 95% patients who become aware of their status at later stages with presentation of cirrhosis, incur the cost of supportive management including the management of associated complications like decompensated disease or hepatocellular carcinoma (HCC).

Figure 1.

Screening with ELISA followed by confirmatory test and treatment - Scenario I

In this scenario, ELISA was used as the screening test followed by HCV RNA as the confirmatory test. The coverage of screening has been assumed to be 30% based on the uptake of screening in other programs[22-24] Those who are then determined eligible, are administered treatment as per the algorithm. A loss to follow up of 10% has been assumed at each step from testing to treatment. Currently, ELISA is being used at the facility level for testing antibodies to HCV. This scenario is compared with the do-nothing/routine-care scenario wherein there is no active screening program in practice.

Screening with RDT followed by confirmatory test and treatment– Scenario II

This scenario differs from the above in the test used for screening. RDT kits are considered instead of ELISA for testing the antibodies to HCV followed by HCV RNA as the confirmatory test and thus treatment. The rationale for comparing both these testing strategies is that use of ELISA based screening tests is associated with long turn-around time, high cost and requirements for

For peer review only - http://bmjopen.bmj.com/site/about/guidelines.xhtml

specialized apparatus and trained technicians whereas, screening with RDTs obviates the need for multiple follow-up appointments as well as shortens the waiting time. Also, RDTs follow a simpler technique as compared to ELISA based screening.[25,26]

In both scenarios, all eligible viremic HCV infected persons will receive free-of-charge DAAs as per the treatment algorithm under the NVHCP. The coverage and loss to follow up assumptions remain the same for either scenario. Similar to above, this strategy is also compared with the routine-care scenario.

Model structure

A decision tree (Figure 2) integrated with a mathematical Markov model (Figure 3), depicting natural history of HCV was prepared in Microsoft Excel to simulate the progression of disease. The costs and health outcomes of each intervention scenario (I and II) are compared with the routine care scenario.

Figure 2 .

A Markov model, with similar transition states, used in a previous analysis to assess the cost effectiveness of using velpatasvir (VEL) for treatment of HCV, was constructed to simulate disease progression among the HCV population.[27]

Figure 3.

A cross-sectional study in Punjab which included 5,543 individuals, reported a prevalence of 3.6% with a viremia rate of 70% thus estimating a burden of around 700,000 viraemic hepatitis C patients.[13] This study also reports age-based prevalence of chronic HCV infection in Punjab.[13] Based on the analysis of the MMPHCRF patient data, the mean age of presentation of HCV infection was estimated to be around 40 years.[15] Thus, in our analysis, we considered one-time universal screening of the Punjab population around the mean age of presentation of HCV.

For peer review only - http://bmjopen.bmj.com/site/about/guidelines.xhtml

Subsequent to treatment, the patients were divided into those who achieve sustained virologic response (SVR), a surrogate marker for virologic cure, and others who do not (treatment failures). The HCV infected persons in fibroisis stages F0-F3, who achieve SVR, are not likely to progress further and thus the progression is halted.[27] Patients with cirrhosis who achieve SVR continue to progress to subsequent stages of liver disease progression, albeit at a slower rate as compared to the natural progression rates and with a lower risk of HCC.[27] Those patients who do not achieve SVR continue to progress to subsequent stages as per the natural progression of the disease.[27] It was assumed that death due to HCV occurs from decompensated cirrhosis and HCC states while all-cause mortality happens in all stages. A cycle length of 1 year was considered appropriate along with a life-time study horizon to comprehensively capture all the costs and benefits of the intervention.

Costing

A societal perspective was considered to analyse total costs for screening and treatment. Apart from the cost of screening and confirmatory tests, treatment associated costs were also included, i.e, cost of out-patient (OPD) consultation for therapeutic purpose, in-patient care for hospitalisation which is required in advanced stages as well as cost of management of complications that occur at each stage of the disease. The cost of specific procedures required in assessment of fibrosis and at later stages such as endoscopy, transient elastography, ultrasound, ascitic tap were also included. Average costs for primary, secondary and tertiary OPD consultation and hospitalisation as reported in recently published Indian studies, as well as National Health System Cost Database were used (Table 1).[28-30] The tertiary sector costs for IPD included both the cost of medicine ward[31] as well as the intensive care unit (ICU) costs. The cost of ELISA and RDT kits were as per the procurement rates of the Punjab government obtained via consultations with program officers. Other costs associated with the test such as cost of equipment, human resource, infrastructures, drugs and consumables, and other non consumable itmes were available

For peer review only - http://bmjopen.bmj.com/site/about/guidelines.xhtml

from data collected for costing the laboratory procedures at the district hospital. The confirmatory test HCV RNA was outsourced by the government and thus the provider payment rates of Punjab government contracted laboratories were used.[15] The cost of DAAs including Ribavirin (RBV), ranging from □ 4000-17000, US $ 57-242 per patient, were also as per the procurement prices in Punjab.[14] All costs are also reported for the current year in USD at a currency exchange rate of 1 USD = 70.5 INR.[32]

We also included the out of pocket (OOP) expenditure of the patient for OPD and hospitalisation in public and private facilities. It was derived based on analysis of unit level data from National Sample Survey 71st round, conducted in during January-June 2014, which is a large household survey covering a sample of 65,932 households.[33]

Valuation of consequences

The impact of intervention was dependent on the sensitivity and specificity of the screening tests, the compliance of each step as well as SVR rates achieved with the treatment provided subsequently. The estimates for sensitivity and specificity of ELISA and RDT fed into the model have been reported by published systematic reviews and meta-analysis.[34,35] It was assumed that 10% (5-20%) of the population was lost during follow up at each step. The impact of treatment has been reported in terms of SVR which was modelled to assess progression to more severe health states and finally in terms of survival. The health-related quality of life was measured using Euro-QOL five dimensions questionnaire (EQ-5D-5L) which was administered to a total of 230 patients who were being treated for chronic HCV infection.[27] We assumed that the quality of life among non-cirrhotic patients who achieve SVR to be equivalent to the general population[36] whereas for the cirrhotic and HCC patients, we assumed that the quality of life score was similar as in the respective advanced stages.

For peer review only - http://bmjopen.bmj.com/site/about/guidelines.xhtml

The estimates for SVR rates were obtained from MMPHCRF data of 48,808 patients.[15,27] All the parameters pertaining to progression of HCV, with and without achieving SVR, were obtained through published literature.[37,38] Annual mortality estimates from DC and HCC were 21.6% and 41.1% respectively and were reported from published literature.[39-41] The age-wise all-cause mortality was obtained from the Sample Registration System report for Punjab state.[42]

Also, we made an assumption regarding the coverage of screening to be 30% in the base case.[23-25] Since, there is no screening program currently running in the state, therefore we tried to put in a modest estimate which is not very high as well as will help to reflect the impact of screening activity.

All the parameters related to costs and effectiveness have been reported in Table 1.

Sensitivity Analysis

We undertook a probabilistic sensitivity analysis (PSA) to analyse the effect of joint parameter uncertainty on the incremental cost-effectiveness ratio (ICER).[43,44] For several parameters, 95% confidence intervals were available such as age-wise prevalence estimates, progression parameters, sensitivity and specificity of screening tests. For other parameters including the stage-wise distribution at the time of diagnosis and SVR rates were varied by 5% for both upper and lower limits, RBV tolerance was varied by 10% for both upper and lower limits. All the drugs costs were varied by 20% on the lower side only as the current prices of DAA are the negotiated prices resulting from operating bulk procurement mechanisms in the state. Therefore, the prices might decrease further but the increase in the prices is unlikely. The cost of screening tests was varied 20% on the lower side and 50% on the upper side. For quality of life estimates, standard errors were computed form the primary analysis.

For undertaking the PSA, gamma distribution was used for all the cost parameters, a beta distribution was used in case of parameters where 95% confidence intervals were available, and a

For peer review only - http://bmjopen.bmj.com/site/about/guidelines.xhtml

uniform distribution was applied where the upper and lower limits were available. Monte Carlo method was used, and the results were simulated 1000 times. We computed the median estimates along with 2.5th and 97.5th percentile to estimate 95% confidence interval.

Based on the PSA, we estimated the probability of being cost-effective at varying willingness to pay thresholds (WTP) for scenario I and II compared with no screening. We used a 1- time per-capita Gross Domestic Product (GDP) value for India as the threshold to base our conclusion about cost-effectiveness. This was in view of the national guidelines for Health Technology Assessment (HTA)[20], current practice in Indian economic evaluations[19,45] and more national discussions.[46]

A scenario analysis was conducted for assessing the cost effectiveness of scenario I and II as compared to the routine care, wherein we modelled the impact of screening and treatment for all people aged 18 years and above. Thus, this scenario analysis primarily evaluated the cost effectiveness of screening which could be attributed to difference in age of initiating the screening.

We also conducted a univariate analysis to assess the impact of varying screening coverage parameter. Thus, we estimated ICERs at coverage rates of 10%, 20%, 50%, 70% and 100%.

Budget Impact Analysis

We undertook a budget impact analysis (BIA) to assess the requirement of resources for the uptake of screening and treatment program. Two scenarios were considered, screening everyone aged 18 years and above and screening the age cohort of 40-45 years which is the mean age of presentation of HCV infection. The parameters considered were: Punjab population estimates from census[47], prevalence estimates from published literature[12-14], cost of screening tests which included ELISA and RDT based on expert consultations with program officers, cost of confirmatory test HCV RNA[15], cost of drugs given for treatment as per the provider payment rates of Punjab government[15], cost incurred on human resources in terms of their salary as well as cost

For peer review only - http://bmjopen.bmj.com/site/about/guidelines.xhtml

of integrated trainings conducted to train personnel for carrying out screening activities and the cost spent on information-education-counselling (IEC) and behaviour change communication (BCC) activities. The cost of integrated trainings and IEC/BCC activities were derived from expert consultations with program officers and the review of state budget documents.

For the human resources, we estimated the man-hours currently being dedicated to HCV screening and therefore we budgeted in terms of additional man-hours required to screen a given proportion of population if screening facilities were to be delivered via existing set up. Cost of training and IEC/BCC have been treated as fixed costs as there is no basis for determining repeated IEC/BCC activities in absence of an active screening program. The cost of screening test, confirmatory test and drugs are the variable costs associated with screening and treatment.

A payer's perspective was considered for the analysis with no discounting and a time horizon of five years. Results were reported at different coverage rates of screening 10 to 100%. The methodology of the budget impact analysis was in consistency with International Society for Pharmacoeconomics and Outcomes Research (ISPOR) reporting framework.[48]

Patient and public involvement

No patient and public involved.

Results:

Costs

In the routine scenario, a lifetime cost of □ 2,775 (US $ 38.8) is incurred per person. In scenario I, a lifetime societal cost of □ 998 (US $ 14.1) is incurred per person whereas in scenario II, □ 947(US $ 13.2) per person are incurred. When scenario I is compared with no screening option, it saves □ 1,810 (US $ 25.3) per person. The cost savings are similar when scenario II is compared with no screening, i.e, □ 1,812 (US $ 25.3) per person.

For peer review only - http://bmjopen.bmj.com/site/about/guidelines.xhtml

For peer review only

Valuation of consequences

The life years lived by Punjab population including HCV infected cohort as well in no screening, scenario I and II were 15.75, 15.92 and 15.76 per person respectively. Similarly, the QALYs lived per person in no screening, scenario I and II were 15.57, 15.78 and 15.6 respectively. As compared to the routine scenario, there was a gain of 0.0046 (0.0013-0.011) life years and 0.028 (0.008-0.06) QALY per person in scenario I. The gains are similar when scenario II is compared with no screening, i.e, 0.0045 (0.0012-0.012) life years and 0.027 (0.008-0.061) QALY per person.

Costs and outcomes for each scenario have been given in Table 2.

Cost-effectiveness

When compared with no screening, both scenarios I and II dominate over no screening, i.e, they have better health outcomes along with being cost saving. However, when the incremental gains of scenario I and II (relative to no screening) are compared, there is no statistically significant difference in outcomes as well as costs.

In addition, when compared to scenario I, scenario II results in an incremental gain of 0.0003 life years and 0.001 QALYs per patient with a saving of Rs 13.73 per patient. The reason for minimal gains is that sensitivity and specificity of both ELISA and RDT are overlapping. However, the of cost of RDT is lesser than ELISA.

Screening is cost saving at all coverage levels from 10 to 100%. Scenario I and II (relative to no-screening), have a 100% probability of being cost saving. Also, screening with both, either ELISA or RDT, followed by treatment for people aged 18 years and above is cost saving. This implies that irrespective of the age of initiation of screening and subsequent treatment, screening is a dominant strategy as compared to no screening. Figure 4 displays cost-effectiveness planes for both the scenarios (ELISA and RDT) as compared to no screening.

For peer review only

403 Figure 4.

404 Budget Impact Analysis

405 Screening the population of 18 years and above, at base coverage of 30%, ELISA costs 1779 million, 406 i.e, 3.8% of the Punjab state health budget[49] whereas with RDT 1586 million rupees, i.e, 3.4% of 407 the state health budget. However, if screening the age cohort of 40-45 years, with ELISA and RDT, 408 account for 0.45% and 0.41% of the state health budget respectively.

409 In addition, given the current human resources, who perform other tests as well in addition to HCV, 410 screening the 18 years and older at a coverage of 30%, would require an addition 186,336 manhours 411 (1260 persons). Similarly, screening the age cohort of 40-45 years would require an additional 412 14,665 manhours (99 persons). However, if one dedicated person to HCV screening is deployed at 413 each of the districts, the human resources will be sufficient to screen the age cohort of 40-45 years 414 whereas, additional 1015 persons would be required to screen everyone 18 years and older.

415 The year wise disaggregated results from the 1st to 5th year (Table 3A), distribution of budget 416 estimates for the base year at 30% coverage according to type of resources (Table 3B) and budget 417 estimates for different coverage levels (Table 3C) are summarised under Table 3.

418 Discussion:

419 In resource limited settings like India, where out of pocket expenditure accounts for 62% of the total 420 health expenditure,[50] it is of utmost importance to utilise the resources in a sustainable manner. The 421 present analysis assessed the sustainability of a proposed screening and treatment program for HCV 422 in the setting of the existing NVHCP. Our findings demonstrate that screening, either with ELISA 423 or RDT, is dominant over no screening which implies having higher QALYs gained and lower costs. 424 Also, the difference between incremental gains for both scenario I and II (compared to no screening) 425 is insignificant which can be attributed to overlapping sensitivity and specificity of ELISA and RDT.

For peer review only - http://bmjopen.bmj.com/site/about/guidelines.xhtml

Treating HCV infection in early stages results in halting the progression to advanced stages which are associated with high management costs.[28] Our previous analysis demonstrated that, given a lifetime horizon, treatment with DAA decreases the cases of decompensated cirrhosis and HCC attributable to HCV by half as compared to no treatment.[27] Secondly, the effectiveness rates of the DAA based treatment are as high as 90%, even in public health programmes.[51-53] Thirdly, the prices of DAA and diagnostic tests have been significantly reduced through price negotiation with bulk procurement of the efficacious generic drugs and reagents. All these factors support the cost saving nature of the screening and treatment intervention for HCV in Punjab.

Though the intervention of screening appears to be dominant, it is associated with a huge budget impact. BIA are financial tools which help us to estimate the future spending required in the chosen time horizon.[54] In light of the results of BIA, one-time screening of 18 years and older population would cost in the range of 3.4% (RDT,scenario II) – 3.8% (ELISA, scenario I) of the state health budget, whereas, screening the population in the age-group of 40-45 years old will require spending 0.41% (RDT, scenario II) - 0.45% (ELISA, scenario I) of the state health budget. One way to assess whether the additional budget for health will be able to accommodate for higher allocation to any program is the analysis of the fiscal space. Fiscal space is dependent on[55]: the macroeconomic factors for which the impact on the GDP is assessed. Secondly, one can generate the estimates for fiscal space through budget reprioritization. Thirdly, we can measure efficiency and estimate monetary value of efficiency enhancing measures. Consequent to this, we compare the required budget for screening with average annual growth in the health budget of Punjab state, which has been reported to 10%.[56] According to the India State-Level Disease Burden Initiative, cirrhosis and other chronic liver diseases account for 1.44% of total morbidity burden in Punjab.[57] Given this scenario, spending more than 0.144% of the health budget for HCV program seems unrealistic. Thus, opting for screening the age-group of 40-45 years with RDT instead of all 18 years and older appears operationally feasible.

For peer review only - http://bmjopen.bmj.com/site/about/guidelines.xhtml

451 Our findings are consistent with other studies in the same domain.[58-60] A model-based analysis
452 evaluated the cost-effectiveness of one-time screening of inividuals between 40-70 years of age
453 compared to no screening in Korea. The findings of the study demonstrate that tone-time screening
454 for HCV was highly cost-effective in comparison to their routine scenario, i.e, no screening.[58]
455 Another model-based cost effectiveness analysis for screening and treatment of HCV in Egypt also
456 demonstrates one-time screening and treatment to be a dominant strategy as compared to no
457 screening.[59]

458 Further, a cost-effectiveness study based in Japan also evaluates the cost-effectiveness of screening
459 and treatment. They compare no screening vs screening and interferon based treatment vs screening
460 and DAA based treatment. Also, they stratified the analysis according to different age group. Similar
461 to our results, this study also concluded that screening the age group of 40-49 years followed by
462 DAA based treatment is the most cost-effective strategy.[60] Another study based in the United states
463 (US) compared one-time universal screening as well as birth cohort screening with risk factor
464 screening. Both types of interventions were deemed cost effective as compared to risk factor-based
465 screening whereas screening among the general population remained cost-effective under all
466 assumptions.[61]

467 Studies conducted pertaining to assess the cost-effectiveness of screening in LMIC are very few.
468 Thus, our analysis would contribute to generate evidence pertaining to this area. We used a
469 biologically plausible model which is fitted with local data on disease epidemiology and
470 effectiveness of DAAs which is drawn from the program data of 48,808 HCV patients on treatment
471 in Punjab. Secondly, cost of treatment is drawn from the National Health System Cost Database and
472 nationally representative household surveys for patient costs. Similarly, the cost of screening is also
473 derived from the real-world application in local context. Thirdly, the data on quality of life is also
474 based on our analysis of primary data collected locally. Thus, all the parameters used in the model
475 are estimates from the real-world setting for which the analysis is being conducted.

For peer review only - http://bmjopen.bmj.com/site/about/guidelines.xhtml

We do acknowledge certain limitations of the present study. We did not consider the possibility of reinfection once treated in our model as we were estimating the impact of one-time screening and treatment. Therefore, we assumed that including reinfection would not change the direction of results. Secondly, the sample sizes for estimating the utility values may not be large enough for different health states. However, this is likely to influence the standard errors; the effect of which is likely to be captured in our probabilistic sensitivity analysis (PSA).

We would also like to mention that we did not include testing for HBV and HIV among those tested for HCV. Fourthly, we did not include productivity losses in our cost analysis. However, as it has also been argued that it is somewhat reflected in the valuation of consequences when trade-off techniques are used to elicit quality of life.[62] Moreover, there are methodological as well as parameter uncertainties which are likely to be introduced when productivity losses are accounted for. Finally, the national HTA guidelines for India have not recommended inclusion of productivity losses.

Conclusion:

Our findings suggest that one-time universal screening followed by treatment of HCV infection is a dominant strategy as compared to no screening. Though, screening is deemed cost saving at all coverage rates but the budget impact of screening program is very high. Thus, based on the ground of both cost-effectiveness and budget impact we recommend screening the age cohort around the mean age of presentation of disease instead of the whole population with RDT instead of ELISA. Implementation of a screening program can improve the uptake of available low cost and highly efficacious drug treatment. Reforms should be introduced to improve both screening and treatment activities to cope up with the rising burden of HCV.

For peer review only - http://bmjopen.bmj.com/site/about/guidelines.xhtml

Author's Contributions:

- Conception: Shankar Prinja, Yashika Chugh, Radha Krishan Dhiman; Design of the work: Yashika Chugh, Shankar prinja, Yot Teerawattananon, Radha Krishan Dhiman; Acquisition: Yashika Chugh, Madhumita Premkumar, Shankar Prinja, Radha Krishan Dhiman, Gagandeep Singh Grover; Analysis: Yashika Chugh, Madhumita Premkumar, Shankar prinja, Yot Teerawattananon; Interpretation of data: Shankar prinja, Yot Teerawattananon, Yashika Chugh, Radha Krishan Dhiman.
- Drafting the work: Yashika Chugh, Madhumita Premkumar; Revising it critically for important intellectual content: Shankar Prinja, Yot Teerawattananon, Radha Krishan Dhiman, Gagandeep Singh Grover.
- Final approval of the version to be published: Yashika Chugh, Shankar Prinja, Yot Teerawattananon, Madhumita Premkumar, Radha Krishan Dhiman, Gagandeep Singh Grover.
- Agreement to be accountable for all aspects of the work in ensuring that questions related to the accuracy or integrity of any part of the work are appropriately investigated and resolved: Yashika Chugh, Shankar Prinja, Yot Teerawattananon, Madhumita Premkumar, Radha Krishan Dhiman, Gagandeep Singh Grover.

Competing interests:

Authors declare no competing interests.

Funding:

The Health Intervention and Technology Assessment Program (HITAP) provided technical supervision for this study under the auspices of the International Decision Support Initiative (iDSI). HITAP is funded by the Thailand Research Fund (TRF) under a grant for a Senior Research Scholar (RTA5980011). HITAP is supported by the International Decision Support Initiative (iDSI) to provide technical assistance on health intervention and technology assessment to governments in low- and middle-income countries. iDSI is funded by the Bill & Melinda Gates Foundation (OPP1134345), the UK's Department for International Development, and the Rockefeller Foundation. The findings, interpretations and conclusions expressed in this article do not necessarily reflect the views of the funding agencies.

Data sharing statement:

All the required data pertaining to the analysis has been provided either in the main text or tables and figures.

For peer review only

542 **References:**

543 1. World Health Organization. Global hepatitis report. Geneva: World Health Organization; 2017. 68 p.

545 2. Hepatitis C [Internet]. Who.int. 2020 [cited 11 February 2020]. Available from: https://www.who.int/en/news-room/fact-sheets/detail/hepatitis-c

547 3. Hepatitis C: simplified curative treatments can drive global scale-up [Internet]. World Health Organization. 2018 [cited 11 February 2020]. Available from: https://www.who.int/hepatitis/news-events/hep-c-scale-up/en/

550 4. World Health Organization. Progress report on access to Hepatitis C treatment: focus on overcoming barriers in low- and middle-income countries. Geneva: World Health Organization; 2018. 51p.

553 5. World Health Organization. Global health sector strategy on viral hepatitis, 2016–2021: Towards ending viral hepatitis. Geneva: World Health Organization; 2016. 53p.

555 6. World Health Organization. Consolidated strategic information guidelines for viral hepatitis: Planning and tracking progress towards elimination. Geneva: World Health Organization; 2019. 28p.

558 7. World Health Organization. Combating hepatitis B and C to reach elimination by 2030. Geneva: World Health Organization; 2016. 17p.

560 8. Government of India. National Action Plan Combating: Viral Hepatitis in India. New Delhi: Ministry of Health and Family Welfare; 2019. 50p.

562 9. Gower E, Estes C, Blach S, Razavi-Shearer K, Razavi H. Global epidemiology and genotype distribution of the hepatitis C virus infection. Journal of hepatology. 2014 Nov 1;61(1):S45-57.

For peer review only

10. Sievert W, Altraif I, Razavi HA, Abdo A, Ahmed EA, Alomair A, et al. A systematic review of hepatitis C virus epidemiology in Asia, Australia and Egypt. Liver Int. 2011;31(SUPPL. 2):61-80.

11. Puri P, Anand AC, Saraswat VA, Acharya SK, Dhiman RK, Aggarwal R, et al. Consensus statement of HCV task force of the Indian National Association for Study of the Liver (INASL). Part I: Status report of HCV infection in India. J Clin Exp Hepatol.2014;4(2):106-16.

12. Dhiman R, Satsangi S, Grover G, Puri P. Tackling the Hepatitis C Disease Burden in Punjab, India. J Clin Exp Hepatol. 2016;6(3):224-232.

13. Sood A, Suryaprasad A, Trickey A, Kanchi S, Midha1 V, M. Foster MA, et al. The burden of hepatitis C virus infection in Punjab, India: A population-based serosurvey. PLoS ONE 2018:13(7): e0200461.

14. Government of Punjab. Mukh Mantri Punjab Hepatitis-C Relief Fund (MMPHCRF)- One Year Journey. Punjab: Department of Health and Family Welfare, Punjab; 2017. 8 p.

15. Dhiman R, Grover G, Premkumar M, Taneja S, Duseja A, Arora S et al. Decentralized care with generic direct-acting antivirals in the management of chronic hepatitis C in a public health care setting. Journal of Hepatology. 2019;71(6):1076-1085.

16. Government of India. National guidelines for Diagnosis & Management of Viral Hepatitis. New Delhi: Ministry of Health and Family Welfare; 2018. 78p.

17. Tan-Torres Edejer T, Baltussen R, Adam T, Hutubessy R, Acharya A, Evans D, et al. Making choices in health: WHO guide to cost-effectiveness analysis. Geneva: World Health Organization; 2003.

18. Prinja S, Bahuguna P, Faujdar D, Jyani G, Srinivasan R, Ghoshal S et al. Cost-effectiveness of human papillomavirus vaccination for adolescent girls in Punjab state:

For peer review only

For peer review only - http://bmjopen.bmj.com/site/about/guidelines.xhtml

Implications for India's universal immunization program. Cancer. 2017;123(17):3253-3260.

19. Prinja S, Chauhan A, Angell B, Gupta I, Jan S. A Systematic Review of the State of Economic Evaluation for Health Care in India. Applied Health Economics and Health Policy. 2015;13(6):595-613.

20. Department of Health Research. Health Technology Assessment in India. New Delhi : Ministry of Health & Family Welfare, Government of India; 2018.

21. World Health Organisation. Viral hepatitis C treatment in India. Available at: http://www.searo.who.int/india/mediacentre/events/2016/viral_hepatitis_c_treatment_in_india.pdf?ua=1 [Accessed on 9 December 2018].

22. Gakidou E, Nordhagen S, Obermeyer Z. Coverage of cervical cancer screening in 57 countries: low average levels and large inequalities. PLoS medicine. 2008 Jun;5(6).

23. Sharma M, Ying R, Tarr G, Barnabas R. Systematic review and meta-analysis of community and facility-based HIV testing to address linkage to care gaps in sub-Saharan Africa. Nature. 2015 Dec;528(7580):S77-85.

24. Coffin PO, Scott JD, Golden MR, Sullivan SD. Cost-effectiveness and population outcomes of general population screening for hepatitis C. Clinical infectious diseases. 2012 May 1;54(9):1259-71.

25. Bassett IV, Walensky RP. Integrating HIV screening into routine health care in resource-limited settings. Clinical Infectious Diseases. 2010 May 15;50(Supplement_3):S77-84.

26. Tang W, Chen W, Amini A, Boeras D, Falconer J, Kelly H, Peeling R, Varsaneux O, Tucker JD, Easterbrook P. Diagnostic accuracy of tests to detect Hepatitis C antibody: a meta-analysis and review of the literature. BMC infectious diseases. 2017 Nov 1;17(1):695.

For peer review only

For peer review only - http://bmjopen.bmj.com/site/about/guidelines.xhtml

27. Chugh Y, Dhiman R, Premkumar M, Prinja S, Singh Grover G, Bahuguna P. Real-world cost-effectiveness of pan-genotypic Sofosbuvir-Velpatasvir combination versus genotype dependent directly acting anti-viral drugs for treatment of hepatitis C patients in the universal coverage scheme of Punjab state in India. PLOS ONE. 2019;14(8):e0221769.

28. Prinja S, Bahuguna P, Duseja A, Kaur M, Chawla YK. Cost of Intensive Care Treatment for Liver Disorders at Tertiary Care Level in India. PharmacoEconomics - Open. 2017:1-12.

29. Prinja S, Sharma D, Aggarwal A, Bahuguna P, Sharma A, Rana S. Out-of-pocket expenditure for hospitalization in Haryana State of India: Extent, determinants & financial risk protection. Indian Journal of Medical Research. 2017;146(6):759.

30. Prinja S, Gupta A, Verma R, Bahuguna P, Kumar D, Kaur M et al. Cost of Delivering Health Care Services in Public Sector Primary and Community Health Centres in North India. PLOS ONE. 2016;11(8):0160986.

31. Kaur G, Prinja S, Malhotra P, Lad D, Prakash G, Khadwal A et al. Cost of Treatment of Multiple Myeloma in a Public Sector Tertiary Care Hospital of North India. 2018.

32. XE: Convert USD/INR. United States Dollar to India Rupee [Internet]. Xe.com. 2020 [cited 16 February 2020]. Available from: https://www.xe.com/currencyconverter/convert/?Amount=1&From=USD&To=INR.

33. National Sample Survey Office.Key Indicators of Social Consumption in India: Health (NSSO 71st Round, January–June 2014). New Delhi: NSSO;2015. 71 p.

34. Khuroo M, Khuroo N, Khuroo M. Diagnostic Accuracy of Point-of-Care Tests for Hepatitis C Virus Infection: A Systematic Review and Meta-Analysis. PLOS ONE. 2015;10(3):e0121450.

For peer review only

For peer review only - http://bmjopen.bmj.com/site/about/guidelines.xhtml

35. Shivkumar S, Peeling R, Jafari Y, Joseph L, Pant Pai N. Accuracy of Rapid and Point-of-Care Screening Tests for Hepatitis C. Annals of Internal Medicine. 2012;157(8):558.

36. Chong CA, Gulamhussein A, Heathcote EJ, Lilly L, Sherman M, Naglie G, et al. Healthstate utilities and quality of life in hepatitis C patients. Am J Gastroenterol. 2003;98:6308.

37. Thein H, Yi Q, Dore G, Krahn M. Estimation of stage-specific fibrosis progression rates in chronic hepatitis C virus infection: A meta-analysis and meta-regression. Hepatology. 2008;48(2):418-431.

38. Van der Meer A, Veldt B, Feld J, Wedemeyer H, Dufour J, Lammert F et al. Association Between Sustained Virological Response and All-Cause Mortality Among Patients With Chronic Hepatitis C and Advanced Hepatic Fibrosis. JAMA. 2012;308(24):2584.

39. Planas R, Ballesté B, Antonio Álvarez M, Rivera M, Montoliu S, Anton Galeras J et al. Natural history of decompensated hepatitis C virus-related cirrhosis. A study of 200 patients. Journal of Hepatology. 2004;40(5):823-830.

40. D'Amico G, Garcia-Tsao G, Pagliaro L. Natural history and prognostic indicators of survival in cirrhosis: A systematic review of 118 studies. Journal of Hepatology. 2006;44(1):217-231.

41. Altekruse S, McGlynn K, Reichman M. Hepatocellular Carcinoma Incidence, Mortality, and Survival Trends in the United States From 1975 to 2005. Journal of Clinical Oncology. 2009;27(9):1485-1491.

42. Registrar General & Census Commissioner of India. SRS life tables 2011-2015. http://www.censusindia.gov.in/Vital_Statistics/SRS_Life_Table/Srs_life_Table_2011-15.html [Accessed 20 August 2018].

For peer review only - http://bmjopen.bmj.com/site/about/guidelines.xhtml

664 43. Briggs A, Weinstein M, Fenwick E, Karnon J, Sculpher M, Paltiel A. Model Parameter Estimation and Uncertainty: A Report of the ISPOR-SMDM Modeling Good Research Practices Task Force-6. Value in Health. 2012;15(6):835-842.

667 44. Claxton K. Exploring Uncertainty in Cost-Effectiveness Analysis. PharmacoEconomics. 2008;26(9):781-798.

669 45. Revill P, Ochalek J, Lomas J, Nakamura R, Woods B, Rollinger A et al. Cost-effectiveness thresholds: guiding health care spending for population health improvement. Centre for Health Economics, University of York; 2015. 24 p.

672 46. Prinja S, Sundararaman T, Muraleedharan VR. Cost-effectiveness Threshold and Health Opportunity Cost. Economic & Political Weekly. 2020 Jan 11;55(2):19.

674 47. Punjab Population Sex Ratio in Punjab Literacy rate data 2011-2020 [Internet]. Census2011.co.in. 2020 [cited 19 February 2020]. Available from: https://www.census2011.co.in/census/state/punjab.html

677 48. Sullivan SD, Mauskopf JA, Annemans L, et al. Principles of good practice for budget impact analysis: report of the ISPOR Task Force on Good Research Practices—Budget Impact Analysis. Value Health. 2007;10(5):336-347.

680 49. Ministry of Health and family welfare, Government of Punjab. Budget 2020-21. Punjab: Government of Punjab; 2020 p. 73.

682 50. Pandey A, Ploubidis GB, Clarke L, Dandona L. Trends in catastrophic health expenditure in India: 1993 to 2014. Bulletin of the World Health Organization. 2018 Jan 1;96(1):18.

685 51. Lee R, Kottilil S, Wilson E. Sofosbuvir/velpatasvir: a pangenotypic drug to simplify HCV therapy. Hepatol Int. 2017;11(2):161-70.

For peer review only

For peer review only - http://bmjopen.bmj.com/site/about/guidelines.xhtml

52. Feld JJ, Jacobson IM, Hézode C, Asselah T, Ruane PJ, Gruener N, et al. Sofosbuvir and Velpatasvir for HCV Genotype 1, 2, 4, 5, and 6 Infection. N Engl J Med. 2015;373(27):2599-607.

53. Nelson DR, Cooper JN, Lalezari JP, et al. All-oral 12-week treatment with daclatasvir plus sofosbuvir in patients with hepatitis C virus genotype 3 infection: ALLY-3 phase 3 study. Hepatology. 2015;61:1127-1135.

54. Sullivan SD, Mauskopf JA, Augustovski F, Caro JJ, Lee KM, Minchin M, et al. Budget impact analysis—principles of good practice: report of the ISPOR 2012 Budget Impact Analysis Good Practice II Task Force. Value Health. 2014;17:4–14.

55. Barroy H, Sparkes S, Dale E, World Health Organization. Assessing fiscal space for health expansion in low-and-middle income countries: a review of the evidence. World Health Organization; 2016.

56. Srinath P, Kotasthane P, Kher D, Chhajer A. A Qualitative and Quantitative Analysis of Public Health Expenditure in India: 2005-06 to 2014-151.

57. GBD India Compare | IHME Viz Hub [Internet]. Vizhub.healthdata.org. 2020 [cited 14 October 2020]. Available from: https://vizhub.healthdata.org/gbd-compare/india

58. Kim DY, Han KH, Jun B, Kim TH, Park S, Ward T, Webster S, McEwan P. Estimating the cost-effectiveness of one-time screening and treatment for hepatitis C in Korea. PLoS One. 2017 Jan 6;12(1):e0167770.

59. Kim DD, Hutton DW, Raouf AA, Salama M, Hablas A, Seifeldin IA, Soliman AS. Cost-effectiveness model for hepatitis C screening and treatment: implications for Egypt and other countries with high prevalence. Global public health. 2015 Mar 16;10(3):296-317.

For peer review only - http://bmjopen.bmj.com/site/about/guidelines.xhtml

710 60. Nagai K, Ide K, Kawasaki Y, Tanaka-Mizuno S, Seto K, Iwane S, Eguchi Y,
711 Kawakami K. Estimating the cost-effectiveness of screening for hepatitis C virus
712 infection in Japan. Hepatology Research. 2020 May;50(5):542-56.
713 61. Coffin PO, Scott JD, Golden MR, Sullivan SD. Cost-effectiveness and population
714 outcomes of general population screening for hepatitis C. Clinical infectious diseases.
715 2012 May 1;54(9):1259-71.
716 62. Pike J, Grosse S. Friction Cost Estimates of Productivity Costs in Cost-of-Illness
717 Studies in Comparison with Human Capital Estimates: A Review. Applied Health
718 Economics and Health Policy. 2018;16(6):765-778.

719

720

721

722

723

724

725

726

727 **Tables**

728 **Table 1: Demographic, epidemiologic, effectiveness and cost-related parameters.**

Parameters	Base value	Lower limit	Upper limit	Distribution	Source
Epidemiological parameters					
Viraemic prevalence of HCV in Punjab	0.026	0.0234	0.0286	Uniform	12-14
19-29 years	0.012	0.005	0.019	Uniform	12-14

For peer review only - http://bmjopen.bmj.com/site/about/guidelines.xhtml

30-39 years	0.031	0.018	0.043	Uniform	12-14
40-49 years	0.047	0.031	0.062	Uniform	12-14
50-59 years	0.045	0.027	0.062	Uniform	12-14
>/=60 years	0.027	0.014	0.039	Uniform	12-14
Stage-wise distribution of HCV at diagnosis					
F0	0.325	0.30875	0.34125	Uniform	16,26
F1	0.325	0.30875	0.34125	Uniform	16,26
F2	0.1	0.095	0.105	Uniform	16,26
F3	0.1	0.095	0.105	Uniform	16,26
F4	0.12	0.114	0.126	Uniform	16,26
DC	0.03	0.0285	0.0315	Uniform	16,26
Proportion of population that is RBV tolerant	0.9	0.81	0.99	Uniform	16,26
Clinical parameters					Expert opinion
F0-F3					
Number of OPD contacts required	3	2	4	Uniform	
Proportion of patients requiring hospitalisations	0.05	0.01	0.1	Uniform	
Number of hospitalisations per patient per year	2	1	4	Uniform	
F4					
Number of OPD contacts required	3	2	4	Uniform	

For peer review only

Proportion of patients requiring hospitalisations	0.05	0.01	0.1	Uniform	
Number of hospitalisations per patient per year	2	1	4	Uniform	
DC					
Number of OPD contacts required	12	6	18	Uniform	
Proportion of patients requiring hospitalisations	0.8	0.7	0.9	Uniform	
Number of hospitalisations per patient per year	6	4	8	Uniform	
HCC					
Number of OPD contacts required	12	6	18	Uniform	
Proportion of patients requiring hospitalisations	0.6	0.5	0.7	Uniform	
Number of hospitalisations per patient per year	2	1	4	Uniform	
Effectiveness parameters					
Quality of life weights					
F0 - F3	0.63	0.57	0.70	Beta	26
F4	0.56	0.51	0.61	Beta	26
DC	0.44	0.38	0.49	Beta	26
HCC	0.44	0.38	0.49	Beta	26
F0-F3 (post SVR)	1	0.83	1	Beta	26,36

For peer review only - http://bmjopen.bmj.com/site/about/guidelines.xhtml

Discount rate	0.03	0.02	0.08	Uniform	17-20
Transition probabilities					
F0 to F1	0.177	0.104	0.13	Beta	38,39
F1 to F2	0.085	0.075	0.096	Beta	38,39
F2 to F3	0.12	0.109	0.133	Beta	38,39
F3 to F4	0.116	0.104	0.129	Beta	38,39
F4 to DC	0.035	0.027	0.043	Beta	38,39
F4 to DC (post SVR)	0.002	0.0001	0.005	Beta	38,39
F4 to HCC	0.024	0.018	0.031	Beta	38,39
F4 to HCC (post SVR)	0.005	0.001	0.009	Beta	38,39
DC to HCC	0.068	0.03	0.083	Beta	38,39
DC to HCC (post SVR)	0.03	0.0225	0.0375	Beta	38,39
Probability of dying due to HCV					
F0 – F4	0	0	0	Uniform	26
DC	0.216	0.162	0.27	Uniform	40-42
HCC	0.411	0.31	0.51	Uniform	40-42
SVR rates (%)					
SOF+DCV (12 weeks)	74	66.5	73.5	Uniform	16,26
SOF+VEL (12 weeks)	84	79.8	88.2	Uniform	16,26
SOF+VEL (24 weeks)	81	76.95	85.05	Uniform	16,26
SOF+VEL+RBV (12 weeks)	84	79.8	88.2	Uniform	16,26

For peer review only - http://bmjopen.bmj.com/site/about/guidelines.xhtml

Sensitivity ELISA	0.952	0.918	0.972	Beta	34,35
Specificity ELISA	0.986	0.976	0.993	Beta	34,35
Sensitivity RDT	0.984	0.889	0.998	Beta	34,35
Specificity RDT	0.986	0.949	0.996	Beta	34,35
Coverage parameters					
Treatment coverage in do-nothing scenario (%)	5	0	10	Uniform	Author's assumption
Coverage of screening in scenario I & II (%)	30	20	50	Uniform	Author's assumption
Loss to follow up at each step (%)	10	5	20	Uniform	Author's assumption
Cost parameters					
Drug costs-12 weeks (□)					
SOF+DCV	4509	3607.2	4509	Gamma	15
SOF+VEL	13104	10483.2	13104	Gamma	15
SOF+VEL+RBV	16818	13454.4	16818	Gamma	15
Cost of diagnostic tests (□)					
ELISA	72.95	58.36	109.425	Gamma	15
RDT	39	31.2	58.5		15
HCV-RNA	880	704	1320	Gamma	15
Routine tests (CBC, LFT, Creatinine)	500	400	750	Gamma	15
Cirrhosis evaluation (Fibro-scan)	350	280	525	Gamma	15
Genotyping	895	716	1342.5	Gamma	15

For peer review only

For peer review only - http://bmjopen.bmj.com/site/about/guidelines.xhtml

Cost per OPD consultation (□)					
Primary	1686.3	1180.41	2192.19	Gamma	28-31
Secondary	1734	1213.8	2254.2	Gamma	28-31
Tertiary	2024	1416.8	2631.2	Gamma	28-31
Cost per patient hospitalisation (□)					
Primary	6347.1	4442.97	8251.23	Gamma	28-31
Secondary	7597	5317.9	9876.1	Gamma	28-31
Tertiary	18693	13085.1	24300.9	Gamma	28-31
Per unit cost of integrated training (□)					
District	50000	40000	75000	Gamma	Expert opinion
State	308000	246400	462000	Gamma	Expert opinion
Per district cost of IEC/BCC (□)	4000	3200	6000	Gamma	Expert opinion

HCV-Hepatitis C virus, DC-Decompensated cirrhosis, HCC-Hepatocellular carcinoma, SOF-Sofosbuvir, LDV-Ledipasvir, RBV-Ribavirin, DCV-Daclatasvir, VEL-Velpatasvir, GT-genotype, OPD- Out-patient Department, IPD- In-patient Department, ELISA: Enzyme Linked Immunosorbent Assay, RDT: Rapid Diagnostic Tests, IEC/BCC: Information-education-counselling and Behaviour change communication, □: Indian rupee.

Table 2: Cost and Effects of Screening and treating Chronic hepatitis C virus infected Patients

**No-Screening**	
Life years per patient	15.75 (11.79-23.85)
QALY per patient	15.57 (11.63-23.60)
Cost per patient (□)	2,775 (1,549-4,898)
**Scenario I**	

For peer review only

For peer review only - http://bmjopen.bmj.com/site/about/guidelines.xhtml

Life years per patient	15.92 (11.79-23.86)
QALY per patient	15.78 (11.65-23.67)
Cost per patient (□)	998 (456-1,932)
**Scenario II**	
Life years per patient	15.75 (11.79-23.86)
QALY per patient	15.60 (11.65-23.63)
Cost per patient (□)	947 (468-1,837)
**Incremental gains**	
Scenario I- No screening	
Life years gained	0.0046 (0.0013-0.011)
QALYs gained	0.028 (0.008-0.061)
Cost difference (□)	-1,810 (-3,376 to – 867)
Scenario II- No screening	
Life years gained	0.0045 (0.001-0.012)
QALYs gained	0.027 (0.008-0.061)
Cost difference (□)	-1,812 (-3,468 to -850)

HCV: Hepatitis C virus, QALY: Quality adjusted life year, □: Indian rupee

Table 3 A: Budget Impact Assessment: Incremental Budgetary estimates spread across the time horizon (year 1 to year 5) in million rupees (million US $)

		ELISA	RDT
**18 Years and older**	Year 1	1779.42 (25.24)	1586.32 (22.5)
	Year 2	52.81 (0.75)	47.1 (0.67)
	Year 3	53.05 (0.75)	47.54 (0.67)

For peer review only - http://bmjopen.bmj.com/site/about/guidelines.xhtml

	Year 4	53.44 (0.76)	48.27 (0.68)
	Year 5	53.93 (0.76)	49.16 (0.7)
**40-45 Years**	Year 1	208.7 (2.96)	190.46 (2.7)
	Year 2	45.83 (0.65)	41.83 (0.59)
	Year 3	46.07 (0.65)	42.07 (0.6)
	Year 4	46.46 (0.66)	42.46 (0.6)
	Year 5	46.94 (0.67)	42.94 (0.61)

ELISA: Enzyme Linked Immunosorbent Assay, RDT: Rapid Diagnostic Tests, US $: United States Dollar.

Table 3 B: Budget Impact Assessment: Resource wise disaggregated estimates of budget for the 1st year at base coverage (30%) in million rupees (million US $)

	**40-45 Years**		**18 Years and older**	
	**ELISA**	**RDT**	**ELISA**	**RDT**
**HR**	1.314 (0.019)	1.314 (0.019)	3.367 (0.048)	3.367 (0.048)

For peer review only
For peer review only - http://bmjopen.bmj.com/site/about/guidelines.xhtml

**IEC/BCC**	0.088 (0.001)	0.088 (0.001)	0.088 (0.001)	0.088 (0.001)
**Screening Test**	35.94 (0.51)	17.7 (0.251)	380.39 (5.396)	187.29 (2.657)
**Confirmatory Test**	22.12 (0.314)	22.12 (0.314)	180.16 (2.555)	180.16 (2.555)
**Drugs**	149.24 (2.117)	149.24 (2.117)	1215.42 (17.24)	1215.42 (17.24)
**Total**	208.702 (2.96)	190.462 (2.702)	1779.425 (25.24)	1586.325 (22.501)

HR: Human resources, IEC/BCC: Information-Education-Counselling-Behaviour-Change-Communication, ELISA: Enzyme Linked Immunosorbent Assay, RDT: Rapid Diagnostic Tests, US $: United States Dollar.

For peer review only

Table 3 C: Budget Impact Assessment: Estimates for different screening scenarios in million rupees (million US $)

Coverage of screening (%)	18 Years and older		Age cohort 40-45 years	
	**ELISA**	**RDT**	**ELISA**	**RDT**

10	593.14 (8.41)	528.78 (7.5)	69.57 (0.99)	63.49 (0.9)
20	1186.28 (16.83)	1057.54 (15)	139.13 (1.97)	126.97 (1.8)
30	1779.42 (25.24)	1586.32 (22.5)	208.7 (2.96)	190.46 (2.7)
50	2965.69 (42.07)	2643.87 (37.5)	347.84 (4.93)	317.44 (4.5)
70	4151.97 (58.89)	3701.41 (52.5)	486.98 (6.91)	444.42 (6.3)
100	5931.39 (84.13)	5287.73 (75)	695.68 (9.87)	634.88 (9.01)

ELISA: Enzyme Linked Immunosorbent Assay, RDT: Rapid Diagnostic Tests, US $: United States Dollar.

For peer review only

Figures

Figure 1: Treatment guidelines for management of Chronic Hepatitis C virus infection.[61]

Figure 2 : Decision tree depicting the screening pathway for different scenarios.

Figure 3: Markov model depicting the natural history of Hepatitis C infection.

For peer review only - http://bmjopen.bmj.com/site/about/guidelines.xhtml

784 Figure 4: Cost-effectiveness planes for scenario I and II vs No screening.

785

786

787

788

For peer review only

For peer review only - http://bmjopen.bmj.com/site/about/guidelines.xhtml

Figure 1: Treatment guidelines for management of Chronic Hepatitis C virus infection.[61]

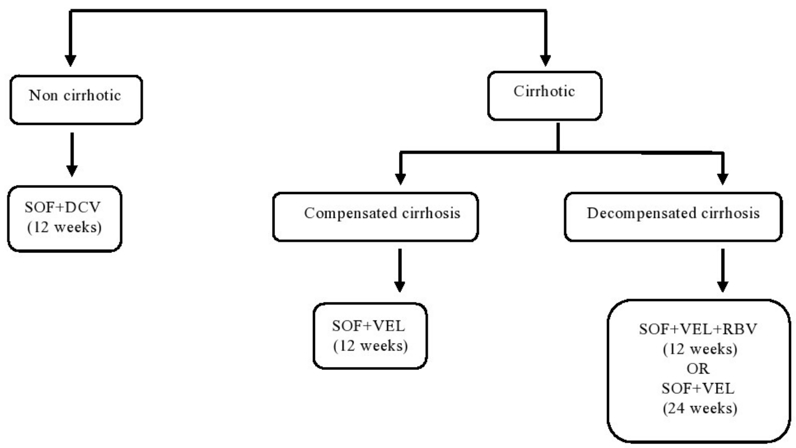

SOF-Sofosbuvir, LDV-Ledipasvir, RBV-Ribavirin, DCV-Daclatasvir, VEL-Velpatasvir

Treatment guidelines for management of Chronic Hepatitis C virus infection

321x294mm (96 x 96 DPI)

Figure 2: Decision tree depicting the screening pathway for different scenarios.

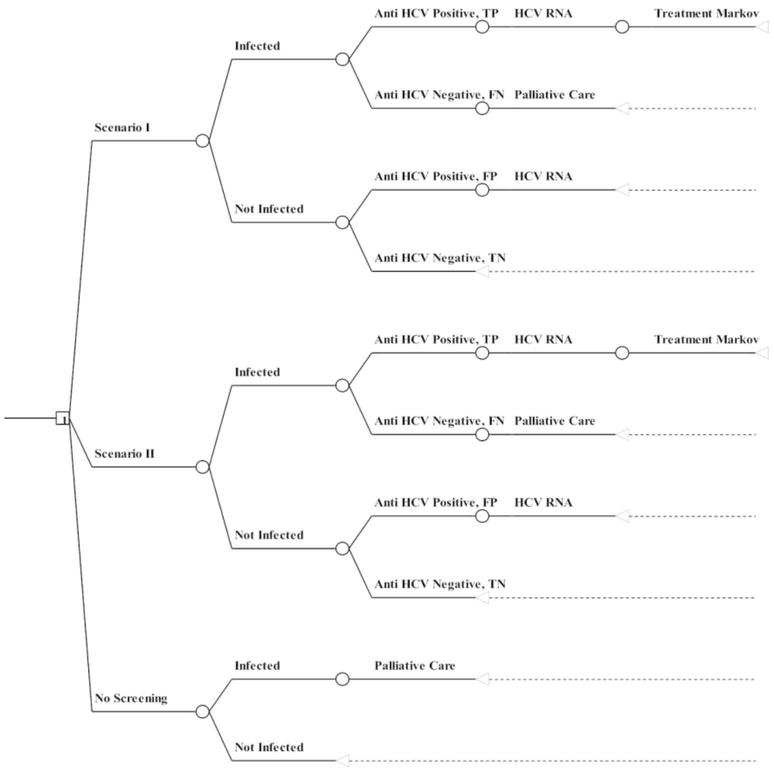

Scenario I: Screening with Enzyme Linked Immunosorbent Assay, Scenario II: Screening with Rapid Diagnostic Tests, HCV: Hepatitis C virus, RNA: Ribo-nucleic Acid, TP: True Positive, TN: True Negative, FN: False Negative, FP: False Positive

Decision tree depicting the screening pathway for different scenarios

583x825mm (72 x 72 DPI)

Figure 3: Markov model depicting the natural history of Hepatitis C infection.

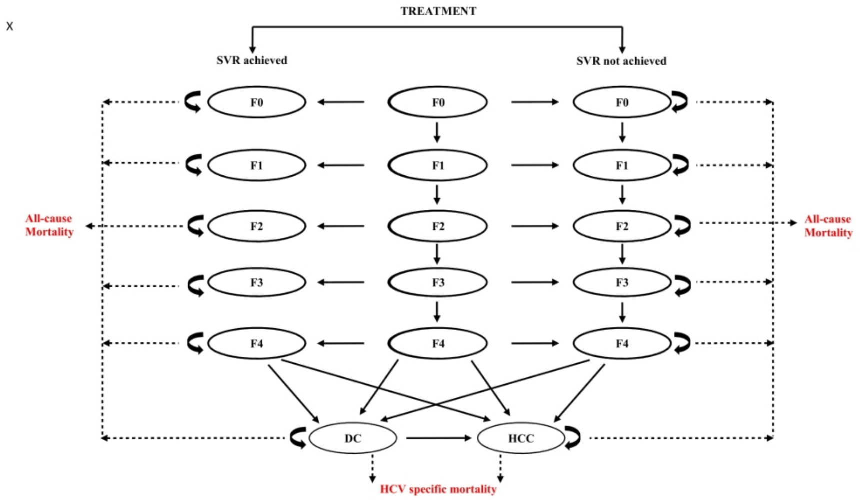

SVR: Sustained Virologic Response, F0: No fibrosis, F: Portal fibrosis without septa, F2: Portal fibrosis with few septa, F3: Numerous septa without cirrhosis, F4: Cirrhosis, DC: Decompensated cirrhosis, HCC: Hepatocellular carcinoma

Markov model depicting the natural history of Hepatitis C infection

825x583mm (72 x 72 DPI)

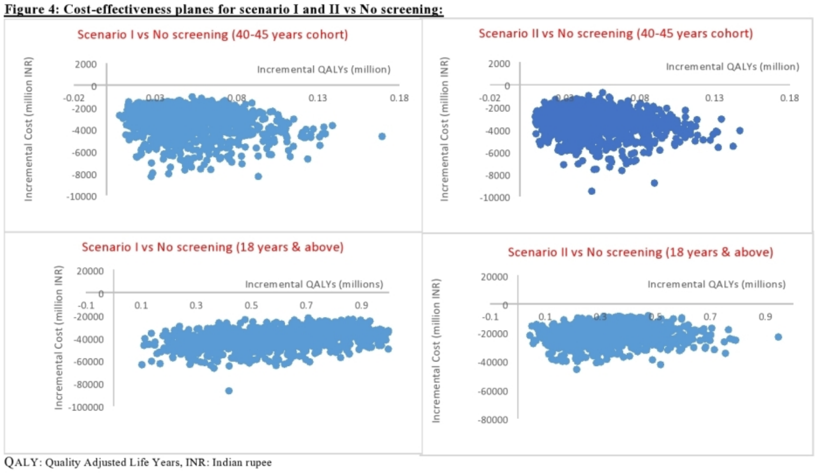

Cost-effectiveness planes for scenario I and II vs No screening

618x437mm (72 x 72 DPI)

CHEERS checklist: Cost-effectiveness and budget impact analysis of screening and treating Hepatitis C infected population vs no screening and treatment in Punjab state of India

Section/item	Item No	Recommendation	Reported on page No/ line No
**Title and abstract**			
Title	1	Identify the study as an economic evaluation or use more specific terms such as "cost-effectiveness analysis", and describe the interventions compared.	Page 1, Line 1-2
Abstract	2	Provide a structured summary of objectives, perspective, setting, methods (including study design and inputs), results (including base case and uncertainty analyses), and conclusions.	Page 3, Line 83-104
**Introduction**			
Background and objectives	3	Provide an explicit statement of the broader context for the study.	Page 5, Line 143-151
		Present the study question and its relevance for health policy or practice decisions.	Page 6, Line 164-170
**Methods**			
Target population and subgroups	4	Describe characteristics of the base case population and subgroups analysed, including why they were chosen.	Page 5, Line 146-149; Page 9, Line 227-233
Setting and location	5	State relevant aspects of the system(s) in which the decision(s) need(s) to be made.	Page 5, Line 146-149, 152-155; Page 6, Line 159-163
Study perspective	6	Describe the perspective of the study and relate this to the costs being evaluated.	Page 9, Line 244-149; Page 10, Line 261-264
Comparators	7	Describe the interventions or strategies being compared and state why they were chosen.	Page 7, Line 190-203; Page 8, Line 204-217
Time horizon	8	State the time horizon(s) over which costs and consequences are being evaluated and say why appropriate.	Page 7, Line 183-184
Discount rate	9	Report the choice of discount rate(s) used for costs and outcomes and say why appropriate.	Page 7, Line 185-189
Choice of health outcomes	10	Describe what outcomes were used as the measure(s) of benefit in the evaluation and their relevance for the type of analysis performed.	Page 7, Line 184-185, 187-189
Measurement of effectiveness	11a	*Single study-based estimates:* Describe fully the design features of the single effectiveness study and why the single study was a sufficient source of clinical effectiveness data.	Page 10, Line 267-270; Page 11, Line 278-279; Page 17, Line 419-422
	11b	*Synthesis-based estimates*: Describe fully the methods used for identification of included studies and synthesis of clinical effectiveness data.	
Measurement and valuation of preference-based outcomes	12	If applicable, describe the population and methods used to elicit preferences for outcomes.	Page 10, Line 270-274
Estimating resources and costs	13a	*Single study-based economic evaluation:* Describe approaches used to estimate resource use associated with the alternative interventions. Describe primary or secondary research methods for valuing each resource item in terms of its unit cost. Describe any adjustments made to approximate to opportunity costs.	Page 9, Line 245-250; Page 10, Line 251-265
	13b	*Model-based economic evaluation:* Describe approaches and data sources used to estimate resource use associated with model health states. Describe primary or secondary research methods for valuing each resource item in terms of its unit cost. Describe any adjustments made to approximate to opportunity costs.	
Currency, price date, and conversion	14	Report the dates of the estimated resource quantities and unit costs. Describe methods for adjusting	Page 10, Line 260-261

For peer review only

Section/item	Item No	Recommendation	Reported on page No/ line No
		estimated unit costs to the year of reported costs if necessary. Describe methods for converting costs into a common currency base and the exchange rate.	
Choice of model	15	Describe and give reasons for the specific type of decision-analytical model used. Providing a figure to show model structure is strongly recommended.	Page 8, Line 219-220; Figure 2 (A & B)
Assumptions	16	Describe all structural or other assumptions underpinning the decision-analytical model.	Page 7, Line 194-196, 203; Page8, Line 205; Page 11, Line 284-287
Analytical methods	17	Describe all analytical methods supporting the evaluation. This could include methods for dealing with skewed, missing, or censored data; extrapolation methods; methods for pooling data; approaches to validate or make adjustments (such as half cycle corrections) to a model; and methods for handling population heterogeneity and uncertainty.	Page 11, Line 290-297; Page 12, Line 298-321; Page 13, Line 322-340
**Results**			
Study parameters	18	Report the values, ranges, references, and, if used, probability distributions for all parameters. Report reasons or sources for distributions used to represent uncertainty where appropriate. Providing a table to show the input values is strongly recommended.	Table 1
Incremental costs and outcomes	19	For each intervention, report mean values for the main categories of estimated costs and outcomes of interest, as well as mean differences between the comparator groups. If applicable, report incremental cost-effectiveness ratios.	Table 2
Characterising uncertainty	20a	*Single study-based economic evaluation:* Describe the effects of sampling uncertainty for the estimated incremental cost and incremental effectiveness parameters, together with the impact of methodological assumptions (such as discount rate, study perspective).	Page 14, Line 363-368; Figure 3
	20b	*Model-based economic evaluation:* Describe the effects on the results of uncertainty for all input parameters, and uncertainty related to the structure of the model and assumptions.	
Characterising heterogeneity	21	If applicable, report differences in costs, outcomes, or cost-effectiveness that can be explained by variations between subgroups of patients with different baseline characteristics or other observed variability in effects that are not reducible by more information.	Page 14, Line 363-368; Figure 3; Table 3 (A, B & C)
**Discussion**			
Study findings, limitations, generalisability, and current knowledge	22	Summarise key study findings and describe how they support the conclusions reached. Discuss limitations and the generalisability of the findings and how the findings fit with current knowledge.	Page 15, Line 381-385, 387-392; Page 16, Line 393-394, 408-417; Page 17, Line 418-441; Page 18, Line 442-443
**7Other**			
Source of funding	23	Describe how the study was funded and the role of the funder in the identification, design, conduct, and reporting of the analysis. Describe other non-monetary sources of support.	Page 1, Line 30-38
Conflicts of interest	24	Describe any potential for conflict of interest of study contributors in accordance with journal policy. In the absence of a journal policy, we recommend authors comply with International Committee of Medical Journal Editors recommendations.	Page 1, Line 40-41

For peer review only

BMJ Open

Cost-effectiveness and budget impact analysis of facility-based screening and treatment of Hepatitis C in Punjab state of India.

Journal:	*BMJ Open*
Manuscript ID	bmjopen-2020-042280.R2
Article Type:	Original research
Date Submitted by the Author:	03-Jan-2021
Complete List of Authors:	Chugh, Yashika; Post Graduate Institute of Medical Education and Research School of Public Health, Department of Community Medicine & School of Public Health<br>Premkumar, Madhumita; Post Graduate Institute of Medical Education and Research, Department of Hepatology<br>Grover, Gagandeep; Department of Health and Family Welfare Punjab<br>Dhiman, Radha K.; Sanjay Gandhi Post Graduate Institute of Medical Sciences<br>Teerawattananon, Yot; Health Intervention and Technology Assessment Program<br>Prinja, Shankar; Post Graduate Institute of Medical Education and Research School of Public Health Department of Community Medicine
**Primary Subject Heading**:	Health economics
Secondary Subject Heading:	Infectious diseases, Health policy, Public health
Keywords:	HEALTH ECONOMICS, Public health < INFECTIOUS DISEASES, Health policy < HEALTH SERVICES ADMINISTRATION & MANAGEMENT

SCHOLARONE™
Manuscripts

I, the Submitting Author has the right to grant and does grant on behalf of all authors of the Work (as defined in the below author licence), an exclusive licence and/or a non-exclusive licence for contributions from authors who are: i) UK Crown employees; ii) where BMJ has agreed a CC-BY licence shall apply, and/or iii) in accordance with the terms applicable for US Federal Government officers or employees acting as part of their official duties; on a worldwide, perpetual, irrevocable, royalty-free basis to BMJ Publishing Group Ltd ("BMJ") its licensees and where the relevant Journal is co-owned by BMJ to the co-owners of the Journal, to publish the Work in this journal and any other BMJ products and to exploit all rights, as set out in our licence.

The Submitting Author accepts and understands that any supply made under these terms is made by BMJ to the Submitting Author unless you are acting as an employee on behalf of your employer or a postgraduate student of an affiliated institution which is paying any applicable article publishing charge ("APC") for Open Access articles. Where the Submitting Author wishes to make the Work available on an Open Access basis (and intends to pay the relevant APC), the terms of reuse of such Open Access shall be governed by a Creative Commons licence – details of these licences and which Creative Commons licence will apply to this Work are set out in our licence referred to above.

Other than as permitted in any relevant BMJ Author's Self Archiving Policies, I confirm this Work has not been accepted for publication elsewhere, is not being considered for publication elsewhere and does not duplicate material already published. I confirm all authors consent to publication of this Work and authorise the granting of this licence.

For peer review only

Title: Cost-effectiveness and budget impact analysis of facility-based screening and treatment of Hepatitis C in Punjab state of India.

Authors: Yashika Chugh[1], Madhumita Premkumar[2], Gagandeep Singh Grover[3], Radha Krishan Dhiman[4,5], Yot Teerawattananon[6], Shankar Prinja[1]

Institution:

1. Department of Community Medicine and School of Public Health, Post Graduate Institute of Medical Education and Research, Chandigarh, India
2. Department of Hepatology, Post Graduate Institute of Medical Education and Research, Chandigarh, India
3. State Program Officer, Hepatitis C Virus Infection, Directorate of Health and Family Welfare, Punjab, India
4. Director, Sanjay Gandhi Postgraduate Institute of Medical Sciences, Lucknow, Uttar Pradesh, India.
5. Chairman, Technical Resource Group - National Viral Hepatitis Control Program (NVHCP), Government of India, India
6. Health Intervention and Technology Assessment Program (HITAP), Ministry of Public Health, Nonthaburi, Thailand & Saw Swee Hock School of Public Health, National University of Singapore.

Short title:
Cost-Effectiveness of universal screening for hepatitis C.

Key words:
cost effectiveness analysis, hepatitis C virus, universal screening, ELISA, RDT, budget impact.

Manuscript details:
367 words abstract, 4756 words main text, 3 tables, 4 figures, 63 references.

Funding:
The Health Intervention and Technology Assessment Program (HITAP) provided technical supervision for this study under the auspices of the International Decision Support Initiative (iDSI). HITAP is funded by the Thailand Research Fund (TRF) under a grant for a Senior Research Scholar (RTA5980011). HITAP is supported by the International Decision Support Initiative (iDSI) to provide technical assistance on health intervention and technology assessment to governments in low- and middle-income countries. iDSI is funded by the Bill & Melinda Gates Foundation (OPP1134345), the UK's Department for International Development, and the Rockefeller Foundation. The findings, interpretations and conclusions expressed in this article do not necessarily reflect the views of the funding agencies.

Conflicts of interest:
No conflicts of interest declared

Ethical Approval:
The present study is a model-based analysis which did not involve any human subjects as well as primary data collection. Therefore, ethical approval was not required. Administrative approval was sought from Department of Health & Family Welfare, Punjab, India.

For peer review only - http://bmjopen.bmj.com/site/about/guidelines.xhtml

For peer review only

Corresponding author:
Dr. Shankar Prinja
Additional Professor of Health Economics
Department of Community Medicine and School of Public Health, Post Graduate Institute of Medical Education and Research
Sector-12, Chandigarh – 160012
Ph: +91 9872871978
Email: shankarprinja@gmail.com

For peer review only - http://bmjopen.bmj.com/site/about/guidelines.xhtml

Cost-effectiveness and budget impact analysis of facility-based screening and treatment of Hepatitis C in Punjab state of India.

Abstract

Objective: Despite treatment availability, chronic Hepatitis C Virus (HCV) public health burden is rising in India due to lack of timely diagnosis. Therefore, we aim to assess incremental cost per quality adjusted life year (QALY) for one-time universal screening followed by treatment of people infected with HCV as compared to a no-screening policy in Punjab, India.

Study design: Decision tree integrated with Markov model was developed to simulate disease progression. A societal perspective and a 3% annual discount rate were considered to assess incremental cost per QALY gained. In addition, budgetary impact was also assessed with a payer's perspective and time horizon of 5 years.

Study setting: Screening services were assumed to be delivered as a facility-based intervention wherein active screening for HCV cases would be performed at 22 district hospitals in the state of Punjab which will act as integrated testing as well as treatment sites for HCV**.**

Intervention: Two intervention scenarios were compared with no universal screening and treatment (routine care). Scenario I – screening with Enzyme Linked Immunosorbent Assay (ELISA) followed by confirmatory HCV-Ribonucleic Acid (RNA) quantification and treatment. Scenario II – screening with Rapid diagnostic test (RDT) kit followed by confirmatory HCV-RNA quantification and treatment.

Primary and secondary outcome measures: Lifetime costs; life years and QALY gained; and Incremental cost-effectiveness ratio (ICER) for each of the above mentioned intervention scenario as compared to the routine care.

Results: Screening with ELISA and RDT, respectively, results in a gain of 0.028 (0.008-0.06) and 0.027 (0.008-0.061) QALY per person with costs decreased by □-1,810 (-3,376 to – 867) and □-1,812 (-3,468 to -850) when compared with no-screening. One-time universal screening of all those ≥18 years at a base coverage of 30%, with ELISA and RDT, would cost 8.5 and 8.3 times more, respectively, when compared to screening the age-group of 40-45 year-old cohort.

Conclusion: One-time universal screening followed by HCV treatment is a dominant strategy as compared to no-screening. However, budget impact of screening of all ≥18 years old people seems unsustainable. Thus, in view of findings from both cost-effectiveness and budget impact, we recommend beginning with screening the age cohort with RDT around mean age of disease presentation, i.e, 40-45 years, instead of all ≥18 years old people.

For peer review only

Article Summary: Strengths and limitations of this study

Strengths

- Our analysis is the first state-level study in India where the country population contributes to around 7.5% of the global disease prevalence.
- We used a biologically plausible model which is fitted with local data on disease epidemiology and effectiveness of DAAs from Punjab, India.
- Cost of treatment is drawn from the National Health System Cost Database and nationally representative household surveys for patient costs.

Limitations

- We did not consider the possibility of reinfection once treated in our model.
- We acknowledge that the sample sizes for estimating the utility values may not be large enough for different health states.

For peer review only

For peer review only - http://bmjopen.bmj.com/site/about/guidelines.xhtml

Introduction:

Nearly 71 million people are living with Hepatitis C virus (HCV) infection round the globe.[1,2] HCV infection led to around 399,000 deaths mainly caused by cirrhosis and hepatocellular carcinoma (HCC).[2] With the advent of all-oral Directly Acting Antiviral agents (DAAs), it is possible to cure 90% of the people infected with HCV, however, the awareness of infection, access to diagnostics and treatment facilities is low.[2,3] Globally, only 1 in 5 people living with HCV infection had been diagnosed.[4] Further, amongst those infected with chronic HCV, only 1% of them have accessed the newer antivirals based treatment.[5]

The World Health Assembly (WHA) released the global targets to eliminate viral hepatitis by 2030.[6] The aim is to diagnose 90% of the Hepatitis B virus (HBV) and HCV infections and treat 80% of the eligible infections by 2030.[6,7] With around 40% of the HCV infected people residing in low and middle income countries (LMICs), India constitutes the 4th highest number of viremic HCV population, where only less than 1% of people are aware of their infection status.[4,8,9]

Though the national prevalence of chronic HCV infection in India ranges from 0.9-1.9%,[10,11] it is as high as 3.6% in the northern state of Punjab.[12,13] The high prevalence in Punjab can be attributed to the high percentage of unsafe medical procedures and blood transfusions.[12] Furthermore, Punjab ranks 2nd amongst the top 10 Indian states with highest number of people who inject drugs (PWID), which is a contributor to increased risk for reinfection as well.[12,14] However, it is difficult to identify such people due to associated stigma.

In 2016, Punjab government launched the "Mukh Mantri Punjab Hepatitis C Relief Fund (MMPHCRF)" for free treatment including DAAs as well as diagnostic as well as monitoring tests required during the treatment of HCV patients.[15] Despite free treatment, due to lack of awareness of disease status, less 10% of the total estimated cases in Punjab have been put on treatment.[16] Thus, along with implementation of treatment strategies there is a need to focus on screening,

For peer review only - http://bmjopen.bmj.com/site/about/guidelines.xhtml

diagnosing those with HCV infection and population education strategies to break the chain of transmission.

With the success of the Punjab Model, where a cure rate of more than 90% was achieved among those enrolled,[15] and in concurrence with the global effort to eliminate HCV infection, India launched the National Viral Hepatitis Control Programme (NVHCP) in July 2018 which aims at improving access to diagnosis and treatment for viral hepatitis.[17] This program covers the entire gamut of acute and chronic viral hepatitis including types A, B, C, D & E with a combined strategy of prevention, immunization for HBV, screening, detection and timely treatment.[17]

In view of these recent development to tackle the HCV burden, the Punjab state government plans to move forward with screening in a phased manner. Screening could be done among high risk groups (HRG) or a universal screening strategy could be one options as identification of HRGs which include patients who are frequently recipients of blood products(e.g. thalassemia patients), PWID, patients on haemodialysis, organ transplant recipients and people living with Human Immunodeficiency virus (PLHIV).[17] However, targeting of sensitive groups, such as, PWID and PLHIV can be associated with stigma which may be accompanied by other political as well as practical challenges.[17] Another viable strategy could be screening around the age of presentation of the infection which is around 40 years from the data base of the 48,088 patients treated under MMPHCRF.[16]

To inform this policy decision on the implementation of a screening and treatment program in the state of Punjab, we undertook this analysis to assess the incremental cost per quality adjusted life year (QALY) for one-time universal screening followed by treatment of HCV infected population as compared to no screening in Punjab. This study also aims to inform on which screening technology represents value for money and would yield a better operational feasibility given the fiscal space for funding the HCV screening program. Thus, a budget impact analysis was

For peer review only - http://bmjopen.bmj.com/site/about/guidelines.xhtml

performed in addition to estimate the financial resources required in terms of initiating a screening program in the state.

Methods:

Overview of Analysis

Screening services were assumed to be delivered as a facility-based intervention wherein active screening for HCV cases would be performed at 22 district hospitals in the state of Punjab, via existing resources, which will act as integrated testing as well as treatment sites for HCV. Since, screening is to be initiated in the state, this assumption was made in consultation with experts from "*National Taskforce to Combat Viral Hepatitis*". We modelled the lifetime costs and outcomes for screening and treating HCV infected patients in Punjab considering 3 different scenarios – no universal screening and treatment of those detected in 'routine care' setting, scenario I – screening with Enzyme Linked Immunosorbent Assay (ELISA) followed by confirmatory HCV-Ribonucleic Acid (RNA) quantification, and scenario II – screening with Rapid diagnostic test (RDT) kit followed by confirmatory HCV-RNA quantification. A lifelong time horizon was considered so as to account for all costs and consequences of the intervention for chronic HCV. The outcomes are valued in terms of the number of HCV deaths, life years and QALYs. All the future costs and consequences, i.e, early vs late detection of HCV, new HCV infections averted and HCV deaths averted, are discounted at a rate of 3%. This choice of discounting rate is based on standard international guidelines along with being consistent with other Indian economic evaluations.[18-21] The methodological principles are in concurrence with the Indian reference case for conducting economic evaluations published by the health technology agency in India.[21]

No screening and treatment/ do-nothing/ Routine-care scenario

For peer review only - http://bmjopen.bmj.com/site/about/guidelines.xhtml

The routine care scenario represents the current situation where-in there is no screening program. In this situation, we assume that patients present to the facility of their own volition when symptoms develop and access the required management support. Since the treatment coverage in India is low[1,22], we also assume that only 5% of the HCV infected cohort receives the testing and treatment on their own and the rest progress to advance stages of HCV as per the natural progression of the disease. The drug regimen followed to treat those infected is as per the national guidelines for diagnosis and management of HCV in India (Figure 1). The rest 95% patients who become aware of their status at later stages with presentation of cirrhosis, incur the cost of supportive management including the management of associated complications like decompensated disease or hepatocellular carcinoma (HCC).

Figure 1.

Screening with ELISA followed by confirmatory test and treatment - Scenario I

In this scenario, ELISA was used as the screening test followed by HCV RNA as the confirmatory test. The coverage of screening has been assumed to be 30% based on the uptake of screening in other programs[23-25] Those who are then determined eligible, are administered treatment as per the algorithm. A loss to follow up of 10% has been assumed at each step from testing to treatment. Currently, ELISA is being used at the facility level for testing antibodies to HCV. This scenario is compared with the do-nothing/routine-care scenario wherein there is no active screening program in practice.

Screening with RDT followed by confirmatory test and treatment– Scenario II

This scenario differs from the above in the test used for screening. RDT kits are considered instead of ELISA for testing the antibodies to HCV followed by HCV RNA as the confirmatory test and thus treatment. The rationale for comparing both these testing strategies is that use of ELISA based screening tests is associated with long turn-around time, high cost and requirements for

For peer review only - http://bmjopen.bmj.com/site/about/guidelines.xhtml

specialized apparatus and trained technicians whereas, screening with RDTs obviates the need for multiple follow-up appointments as well as shortens the waiting time. Also, RDTs follow a simpler technique as compared to ELISA based screening.[26,27]

In both scenarios, all eligible viremic HCV infected persons will receive free-of-charge DAAs as per the treatment algorithm under the NVHCP. The coverage and loss to follow up assumptions remain the same for either scenario. Similar to above, this strategy is also compared with the routine-care scenario.

Model structure

A decision tree (Figure 2) integrated with a mathematical Markov model (Figure 3), depicting natural history of HCV was prepared in Microsoft Excel to simulate the progression of disease. The costs and health outcomes of each intervention scenario (I and II) are compared with the routine care scenario.

Figure 2.

Figure 3.

A cross-sectional study in Punjab which included 5,543 individuals, reported a prevalence of 3.6% with a viremia rate of 70% thus estimating a burden of around 700,000 viraemic hepatitis C patients.[13] This study also reports age-based prevalence of chronic HCV infection in Punjab.[13] Based on the analysis of the MMPHCRF patient data, the mean age of presentation of HCV infection was estimated to be around 40 years.[16] Thus, in our analysis, we considered one-time universal screening of the Punjab population around the mean age of presentation of HCV. Subsequent to treatment, the patients were divided into those who achieve sustained virologic response (SVR), a surrogate marker for virologic cure, and others who do not (treatment failures). The HCV infected persons in fibroisis stages F0-F3, who achieve SVR, are not likely to progress further and thus the progression is halted.[28] Patients with cirrhosis who achieve SVR continue to

For peer review only - http://bmjopen.bmj.com/site/about/guidelines.xhtml

For peer review only

progress to subsequent stages of liver disease progression, albeit at a slower rate as compared to the natural progression rates and with a lower risk of HCC.[28] Those patients who do not achieve SVR continue to progress to subsequent stages as per the natural progression of the disease.[28] It was assumed that death due to HCV occurs from decompensated cirrhosis and HCC states while all-cause mortality happens in all stages. A cycle length of 1 year was considered appropriate along with a life-time study horizon to comprehensively capture all the costs and benefits of the intervention.

For peer review only

Costing

A societal perspective was considered to analyse total costs for screening and treatment. Apart from the cost of screening and confirmatory tests, treatment associated costs were also included, i.e, cost of out-patient (OPD) consultation for therapeutic purpose, in-patient care for hospitalisation which is required in advanced stages as well as cost of management of complications that occur at each stage of the disease. The cost of specific procedures required in assessment of fibrosis and at later stages such as endoscopy, transient elastography, ultrasound, ascitic tap were also included. Average costs for primary, secondary and tertiary OPD consultation and hospitalisation as reported in recently published Indian studies, as well as National Health System Cost Database were used (Table 1).[29-31] The tertiary sector costs for IPD included both the cost of medicine ward[32] as well as the intensive care unit (ICU) costs. The cost of ELISA and RDT kits were as per the procurement rates of the Punjab government obtained via consultations with program officers. Other costs associated with the test such as cost of equipment, human resource, infrastructures, drugs and consumables, and other non consumable itmes were available from data collected for costing the laboratory procedures at the district hospital. The confirmatory test HCV RNA was outsourced by the government and thus the provider payment rates of Punjab government contracted laboratories were used.[16] The cost of DAAs including Ribavirin (RBV), ranging from □ 4000-17000, US $ 57-242 per patient, were also as per the procurement prices in

For peer review only - http://bmjopen.bmj.com/site/about/guidelines.xhtml

Punjab.[15] All costs are also reported for the current year in USD at a currency exchange rate of 1 USD = 70.5 INR.[33]

We also included the out of pocket (OOP) expenditure of the patient for OPD and hospitalisation in public and private facilities. It was derived based on analysis of unit level data from National Sample Survey 71st round, conducted in during January-June 2014, which is a large household survey covering a sample of 65,932 households.[34]

Valuation of consequences

The impact of intervention was dependent on the sensitivity and specificity of the screening tests, the compliance of each step as well as SVR rates achieved with the treatment provided subsequently. The estimates for sensitivity and specificity of ELISA and RDT fed into the model have been reported by published systematic reviews and meta-analysis.[35,36] It was assumed that 10% (5-20%) of the population was lost during follow up at each step. The impact of treatment has been reported in terms of SVR which was modelled to assess progression to more severe health states and finally in terms of survival. The health-related quality of life was measured using Euro-QOL five dimensions questionnaire (EQ-5D-5L) which was administered to a total of 230 patients who were being treated for chronic HCV infection.[28] We assumed that the quality of life among non-cirrhotic patients who achieve SVR to be equivalent to the general population[37] whereas for the cirrhotic and HCC patients, we assumed that the quality of life score was similar as in the respective advanced stages.

The estimates for SVR rates were obtained from MMPHCRF data of 48,808 patients.[16,28] All the parameters pertaining to progression of HCV, with and without achieving SVR, were obtained through published literature.[38,39] Annual mortality estimates from DC and HCC were 21.6% and 41.1% respectively and were reported from published literature.[40-42] The age-wise all-cause mortality was obtained from the Sample Registration System report for Punjab state.[43]

For peer review only - http://bmjopen.bmj.com/site/about/guidelines.xhtml

309 Also, we made an assumption regarding the coverage of screening to be 30% in the base case.[24-26]
310 Since, there is no screening program currently running in the state, therefore we tried to put in a
311 modest estimate which is not very high as well as will help to reflect the impact of screening
312 activity.

313 All the parameters related to costs and effectiveness have been reported in Table 1.

314 **Sensitivity Analysis**

315 We undertook a probabilistic sensitivity analysis (PSA) to analyse the effect of joint parameter
316 uncertainty on the incremental cost-effectiveness ratio (ICER).[44,45] For several parameters, 95%
317 confidence intervals were available such as age-wise prevalence estimates, progression
318 parameters, sensitivity and specificity of screening tests. For other parameters including the stage-
319 wise distribution at the time of diagnosis and SVR rates were varied by 5% for both upper and
320 lower limits, RBV tolerance was varied by 10% for both upper and lower limits. All the drugs
321 costs were varied by 20% on the lower side only as the current prices of DAA are the negotiated
322 prices resulting from operating bulk procurement mechanisms in the state. Therefore, the prices
323 might decrease further but the increase in the prices is unlikely. The cost of screening tests was
324 varied 20% on the lower side and 50% on the upper side. For quality of life estimates, standard
325 errors were computed form the primary analysis.

326 For undertaking the PSA, gamma distribution was used for all the cost parameters, a beta
327 distribution was used in case of parameters where 95% confidence intervals were available, and a
328 uniform distribution was applied where the upper and lower limits were available. Monte Carlo
329 method was used, and the results were simulated 1000 times. We computed the median estimates
330 along with 2.5th and 97.5th percentile to estimate 95% confidence interval.

331 Based on the PSA, we estimated the probability of being cost-effective at varying willingness to
332 pay thresholds (WTP) for scenario I and II compared with no screening. We used a 1- time per-

For peer review only - http://bmjopen.bmj.com/site/about/guidelines.xhtml

capita Gross Domestic Product (GDP) value for India as the threshold to base our conclusion about cost-effectiveness. This was in view of the national guidelines for Health Technology Assessment (HTA)[21], current practice in Indian economic evaluations[20,46] and more national discussions.[47]

A scenario analysis was conducted for assessing the cost effectiveness of scenario I and II as compared to the routine care, wherein we modelled the impact of screening and treatment for all people aged 18 years and above. Thus, this scenario analysis primarily evaluated the cost effectiveness of screening which could be attributed to difference in age of initiating the screening.

We also conducted a univariate analysis to assess the impact of varying screening coverage parameter. Thus, we estimated ICERs at coverage rates of 10%, 20%, 50%, 70% and 100%.

Budget Impact Analysis

We undertook a budget impact analysis (BIA) to assess the requirement of resources for the uptake of screening and treatment program. Two scenarios were considered, screening everyone aged 18 years and above and screening the age cohort of 40-45 years which is the mean age of presentation of HCV infection. The parameters considered were: Punjab population estimates from census[48], prevalence estimates from published literature[12,13,15], cost of screening tests which included ELISA and RDT based on expert consultations with program officers, cost of confirmatory test HCV RNA[16], cost of drugs given for treatment as per the provider payment rates of Punjab government[16], cost incurred on human resources in terms of their salary as well as cost of integrated trainings conducted to train personnel for carrying out screening activities and the cost spent on information-education-counselling (IEC) and behaviour change communication (BCC) activities. The cost of integrated trainings and IEC/BCC activities were derived from expert consultations with program officers and the review of state budget documents.

For peer review only - http://bmjopen.bmj.com/site/about/guidelines.xhtml

For the human resources, we estimated the man-hours currently being dedicated to HCV screening and therefore we budgeted in terms of additional man-hours required to screen a given proportion of population if screening facilities were to be delivered via existing set up. Cost of training and IEC/BCC have been treated as fixed costs as there is no basis for determining repeated IEC/BCC activities in absence of an active screening program. The cost of screening test, confirmatory test and drugs are the variable costs associated with screening and treatment.

A payer's perspective was considered for the analysis with no discounting and a time horizon of five years. Results were reported at different coverage rates of screening 10 to 100%. The methodology of the budget impact analysis was in consistency with International Society for Pharmacoeconomics and Outcomes Research (ISPOR) reporting framework.[49]

Patient and public involvement

No patient and public involved.

Results:

Costs

In the routine scenario, a lifetime cost of □ 2,775 (US $ 38.8) is incurred per person. In scenario I, a lifetime societal cost of □ 998 (US $ 14.1) is incurred per person whereas in scenario II, □ 947(US $ 13.2) per person are incurred. When scenario I is compared with no screening option, it saves □ 1,810 (US $ 25.3) per person. The cost savings are similar when scenario II is compared with no screening, i.e, □ 1,812 (US $ 25.3) per person.

Valuation of consequences

The life years lived by Punjab population including HCV infected cohort as well in no screening, scenario I and II were 15.75, 15.92 and 15.76 per person respectively. Similarly, the QALYs lived per person in no screening, scenario I and II were 15.57, 15.78 and 15.6 respectively. As compared

For peer review only

For peer review only - http://bmjopen.bmj.com/site/about/guidelines.xhtml

to the routine scenario, there was a gain of 0.0046 (0.0013-0.011) life years and 0.028 (0.008-0.06) QALY per person in scenario I. The gains are similar when scenario II is compared with no screening, i.e, 0.0045 (0.0012-0.012) life years and 0.027 (0.008-0.061) QALY per person.

Costs and outcomes for each scenario have been given in Table 2.

Cost-effectiveness

When compared with no screening, both scenarios I and II dominate over no screening, i.e, they have better health outcomes along with being cost saving. However, when the incremental gains of scenario I and II (relative to no screening) are compared, there was an overlap between the confidence intervals of the incremental gains for both costs and outcomes which implies that the difference is statistically insignificant.

In addition, when compared to scenario I, scenario II results in an incremental gain of 0.0003 life years and 0.001 QALYs per patient with a saving of Rs 13.73 per patient. The reason for minimal gains is that sensitivity and specificity of both ELISA and RDT are overlapping. However, the cost of RDT is lesser than ELISA.

Screening is cost saving at all coverage levels from 10 to 100%. Scenario I and II (relative to no-screening), have a 100% probability of being cost saving. Also, screening with both, either ELISA or RDT, followed by treatment for people aged 18 years and above is cost saving. This implies that irrespective of the age of initiation of screening and subsequent treatment, screening is a dominant strategy as compared to no screening. Figure 4 displays cost-effectiveness planes for both the scenarios (ELISA and RDT) as compared to no screening.

Figure 4.

Budget Impact Analysis

For peer review only

For peer review only - http://bmjopen.bmj.com/site/about/guidelines.xhtml

Screening the population of 18 years and above, at base coverage of 30%, ELISA costs 1779 million, i.e, 3.8% of the Punjab state health budget[50] whereas with RDT 1586 million rupees, i.e, 3.4% of the state health budget. However, if screening the age cohort of 40-45 years, with ELISA and RDT, account for 0.45% and 0.41% of the state health budget respectively.

In addition, given the current human resources, who perform other tests as well in addition to HCV, screening the 18 years and older at a coverage of 30%, would require an addition 186,336 manhours (1260 persons). Similarly, screening the age cohort of 40-45 years would require an additional 14,665 manhours (99 persons). However, if one dedicated person to HCV screening is deployed at each of the districts, the human resources will be sufficient to screen the age cohort of 40-45 years whereas, additional 1015 persons would be required to screen everyone 18 years and older.

The year wise disaggregated results from the 1st to 5th year (Table 3A), distribution of budget estimates for the base year at 30% coverage according to type of resources (Table 3B) and budget estimates for different coverage levels (Table 3C) are summarised under Table 3.

Discussion:

In resource limited settings like India, where out of pocket expenditure accounts for 62% of the total health expenditure,[51] it is of utmost importance to utilise the resources in a sustainable manner. The present analysis assessed the sustainability of a proposed screening and treatment program for HCV in the setting of the existing NVHCP. Our findings demonstrate that screening, either with ELISA or RDT, is dominant over no screening which implies having higher QALYs gained and lower costs. Also, the difference between incremental gains for both scenario I and II (compared to no screening) is insignificant which can be attributed to overlapping sensitivity and specificity of ELISA and RDT.

Treating HCV infection in early stages results in halting the progression to advanced stages which are associated with high management costs.[29] Our previous analysis demonstrated that, given a lifetime horizon, treatment with DAA decreases the cases of decompensated cirrhosis and HCC

For peer review only - http://bmjopen.bmj.com/site/about/guidelines.xhtml

attributable to HCV by half as compared to no treatment.[28] Secondly, the effectiveness rates of the DAA based treatment are as high as 90%, even in public health programmes.[52-54] Thirdly, the prices of DAA and diagnostic tests have been significantly reduced through price negotiation with bulk procurement of the efficacious generic drugs and reagents. All these factors support the cost saving nature of the screening and treatment intervention for HCV in Punjab.

Though the intervention of screening appears to be dominant, it is associated with a huge budget impact. BIA are financial tools which help us to estimate the future spending required in the chosen time horizon.[55] In light of the results of BIA, one-time screening of 18 years and older population would cost in the range of 3.4% (RDT,scenario II) – 3.8% (ELISA, scenario I) of the state health budget, whereas, screening the population in the age-group of 40-45 years old will require spending 0.41% (RDT, scenario II) - 0.45% (ELISA, scenario I) of the state health budget. One way to assess whether the additional budget for health will be able to accommodate for higher allocation to any program is the analysis of the fiscal space. Fiscal space is dependent on[56]: the macroeconomic factors for which the impact on the GDP is assessed. Secondly, one can generate the estimates for fiscal space through budget reprioritization. Thirdly, we can measure efficiency and estimate monetary value of efficiency enhancing measures. Consequent to this, we compare the required budget for screening with average annual growth in the health budget of Punjab state, which has been reported to 10%.[57] According to the India State-Level Disease Burden Initiative, cirrhosis and other chronic liver diseases account for 1.44% of total morbidity burden in Punjab.[58] Given this scenario, spending more than 0.144% of the health budget for HCV program seems unrealistic. Thus, opting for screening the age-group of 40-45 years with RDT instead of all 18 years and older appears operationally feasible.

Our findings are consistent with other studies in the same domain.[59-61] A model-based analysis evaluated the cost-effectiveness of one-time screening of inividuals between 40-70 years of age compared to no screening in Korea. The findings of the study demonstrate that one-time screening

For peer review only - http://bmjopen.bmj.com/site/about/guidelines.xhtml

451 for HCV was highly cost-effective in comparison to their routine scenario, i.e, no screening.[59]
452 Another model-based cost effectiveness analysis for screening and treatment of HCV in Egypt also
453 demonstrates one-time screening and treatment to be a dominant strategy as compared to no
454 screening.[60]

455 Further, a cost-effectiveness study based in Japan also evaluates the cost-effectiveness of screening
456 and treatment. They compare no screening vs screening and interferon based treatment vs screening
457 and DAA based treatment. Also, they stratified the analysis according to different age group. Similar
458 to our results, this study also concluded that screening the age group of 40-49 years followed by
459 DAA based treatment is the most cost-effective strategy.[61] Another study based in the United states
460 (US) compared one-time universal screening as well as birth cohort screening with risk factor
461 screening. Both types of interventions were deemed cost effective as compared to risk factor-based
462 screening whereas screening among the general population remained cost-effective under all
463 assumptions.[62]

464 Studies conducted pertaining to assess the cost-effectiveness of screening in LMIC are very few.
465 Thus, our analysis would contribute to generate evidence pertaining to this area. We used a
466 biologically plausible model which is fitted with local data on disease epidemiology and
467 effectiveness of DAAs which is drawn from the program data of 48,808 HCV patients on treatment
468 in Punjab. Secondly, cost of treatment is drawn from the National Health System Cost Database and
469 nationally representative household surveys for patient costs. Similarly, the cost of screening is also
470 derived from the real-world application in local context. Thirdly, the data on quality of life is also
471 based on our analysis of primary data collected locally. Thus, all the parameters used in the model
472 are estimates from the real-world setting for which the analysis is being conducted.

473 We do acknowledge certain limitations of the present study. We did not consider the possibility of
474 reinfection once treated in our model as we were estimating the impact of one-time screening and

For peer review only - http://bmjopen.bmj.com/site/about/guidelines.xhtml

treatment. Therefore, we assumed that including reinfection would not change the direction of results. Secondly, the sample sizes for estimating the utility values may not be large enough for different health states. However, this is likely to influence the standard errors; the effect of which is likely to be captured in our probabilistic sensitivity analysis (PSA).

We would also like to mention that we did not include testing for HBV and HIV among those tested who tested positive for HCV. Fourthly, we assumed the same rate of lost to follow up and adherence to treatment among patients diagnosed through universal screening and those who are seeking for treatment. However, these estimates are unlikely to alter the direction of results as the proportion of people seeking diagnostic and treatment services for HCV on their own is very low.[4,8,9]

Finally, we did not include productivity losses in our cost analysis. However, as it has also been argued that it is somewhat reflected in the valuation of consequences when trade-off techniques are used to elicit quality of life.[63] Moreover, there are methodological as well as parameter uncertainties which are likely to be introduced when productivity losses are accounted for. Finally, the national HTA guidelines for India have not recommended inclusion of productivity losses.

Conclusion:

Our findings suggest that one-time universal screening followed by treatment of HCV infection is a dominant strategy as compared to no screening. Though, screening is deemed cost saving at all coverage rates but the budget impact of screening program is very high. Thus, based on the ground of both cost-effectiveness and budget impact we recommend screening the age cohort around the mean age of presentation of disease instead of the whole population with RDT instead of ELISA. Implementation of a screening program can improve the uptake of available low cost and highly efficacious drug treatment. Reforms should be introduced to improve both screening and treatment activities to cope up with the rising burden of HCV.

For peer review only

For peer review only - http://bmjopen.bmj.com/site/about/guidelines.xhtml

Author's Contributions:

- Conception: Shankar Prinja, Yashika Chugh, Radha Krishan Dhiman; Design of the work: Yashika Chugh, Shankar prinja, Yot Teerawattananon, Radha Krishan Dhiman; Acquisition: Yashika Chugh, Madhumita Premkumar, Shankar Prinja, Radha Krishan Dhiman, Gagandeep Singh Grover; Analysis: Yashika Chugh, Madhumita Premkumar, Shankar prinja, Yot Teerawattananon; Interpretation of data: Shankar prinja, Yot Teerawattananon, Yashika Chugh, Radha Krishan Dhiman.
- Drafting the work: Yashika Chugh, Madhumita Premkumar; Revising it critically for important intellectual content: Shankar Prinja, Yot Teerawattananon, Radha Krishan Dhiman, Gagandeep Singh Grover.
- Final approval of the version to be published: Yashika Chugh, Shankar Prinja, Yot Teerawattananon, Madhumita Premkumar, Radha Krishan Dhiman, Gagandeep Singh Grover.
- Agreement to be accountable for all aspects of the work in ensuring that questions related to the accuracy or integrity of any part of the work are appropriately investigated and resolved: Yashika Chugh, Shankar Prinja, Yot Teerawattananon, Madhumita Premkumar, Radha Krishan Dhiman, Gagandeep Singh Grover.

Competing interests:

Authors declare no competing interests.

Funding:

The Health Intervention and Technology Assessment Program (HITAP) provided technical supervision for this study under the auspices of the International Decision Support Initiative (iDSI). HITAP is funded by the Thailand Research Fund (TRF) under a grant for a Senior Research Scholar (RTA5980011). HITAP is supported by the International Decision Support Initiative (iDSI) to provide technical assistance on health intervention and technology assessment to governments in low- and middle-income countries. iDSI is funded by the Bill & Melinda Gates Foundation (OPP1134345), the UK's Department for International Development, and the Rockefeller Foundation. The findings, interpretations and conclusions expressed in this article do not necessarily reflect the views of the funding agencies.

Data sharing statement:

All the required data pertaining to the analysis has been provided either in the main text or tables and figures.

For peer review only - http://bmjopen.bmj.com/site/about/guidelines.xhtml

References:

1. World Health Organization. Global hepatitis report. Geneva: World Health Organization; 2017. 68 p.
2. Hepatitis C [Internet]. Who.int. 2020 [cited 11 February 2020]. Available from: https://www.who.int/en/news-room/fact-sheets/detail/hepatitis-c
3. Hepatitis C: simplified curative treatments can drive global scale-up [Internet]. World Health Organization. 2018 [cited 11 February 2020]. Available from: https://www.who.int/hepatitis/news-events/hep-c-scale-up/en/
4. World Health Organization. Progress report on access to Hepatitis C treatment: focus on overcoming barriers in low- and middle-income countries. Geneva: World Health Organization; 2018. 51p.
5. World Health Organization. Global health sector strategy on viral hepatitis, 2016–2021: Towards ending viral hepatitis. Geneva: World Health Organization; 2016. 53p.
6. World Health Organization. Consolidated strategic information guidelines for viral hepatitis: Planning and tracking progress towards elimination. Geneva: World Health Organization; 2019. 28p.
7. World Health Organization. Combating hepatitis B and C to reach elimination by 2030. Geneva: World Health Organization; 2016. 17p.
8. Government of India. National Action Plan Combating: Viral Hepatitis in India. New Delhi: Ministry of Health and Family Welfare; 2019. 50p.
9. Gower E, Estes C, Blach S, Razavi-Shearer K, Razavi H. Global epidemiology and genotype distribution of the hepatitis C virus infection. Journal of hepatology. 2014 Nov 1;61(1):S45-57.

For peer review only - http://bmjopen.bmj.com/site/about/guidelines.xhtml

10. Sievert W, Altraif I, Razavi HA, Abdo A, Ahmed EA, Alomair A, et al. A systematic review of hepatitis C virus epidemiology in Asia, Australia and Egypt. Liver Int. 2011;31(SUPPL. 2):61-80.

11. Puri P, Anand AC, Saraswat VA, Acharya SK, Dhiman RK, Aggarwal R, et al. Consensus statement of HCV task force of the Indian National Association for Study of the Liver (INASL). Part I: Status report of HCV infection in India. J Clin Exp Hepatol.2014;4(2):106-16.

12. Dhiman R, Satsangi S, Grover G, Puri P. Tackling the Hepatitis C Disease Burden in Punjab, India. J Clin Exp Hepatol. 2016;6(3):224-232.

13. Sood A, Suryaprasad A, Trickey A, Kanchi S, Midha1 V, M. Foster MA, et al. The burden of hepatitis C virus infection in Punjab, India: A population-based serosurvey. PLoS ONE 2018:13(7): e0200461.

14. Ambekar A, Agrawal A, Rao R, Mishra AK, Khandelwal SK, Chadda RK. Magnitude of substance use in India. New Delhi: Ministry of Social Justice and Empowerment, Government of India. 2019.

15. Government of Punjab. Mukh Mantri Punjab Hepatitis-C Relief Fund (MMPHCRF)-One Year Journey. Punjab: Department of Health and Family Welfare, Punjab; 2017. 8 p.

16. Dhiman R, Grover G, Premkumar M, Taneja S, Duseja A, Arora S et al. Decentralized care with generic direct-acting antivirals in the management of chronic hepatitis C in a public health care setting. Journal of Hepatology. 2019;71(6):1076-1085.

17. Government of India. National guidelines for Diagnosis & Management of Viral Hepatitis. New Delhi: Ministry of Health and Family Welfare; 2018. 78p.

For peer review only - http://bmjopen.bmj.com/site/about/guidelines.xhtml

18. Tan-Torres Edejer T, Baltussen R, Adam T, Hutubessy R, Acharya A, Evans D, et al. Making choices in health: WHO guide to cost-effectiveness analysis. Geneva: World Health Organization; 2003.

19. Prinja S, Bahuguna P, Faujdar D, Jyani G, Srinivasan R, Ghoshal S et al. Cost-effectiveness of human papillomavirus vaccination for adolescent girls in Punjab state: Implications for India's universal immunization program. Cancer. 2017;123(17):3253-3260.

20. Prinja S, Chauhan A, Angell B, Gupta I, Jan S. A Systematic Review of the State of Economic Evaluation for Health Care in India. Applied Health Economics and Health Policy. 2015;13(6):595-613.

21. Department of Health Research. Health Technology Assessment in India. New Delhi : Ministry of Health & Family Welfare, Government of India; 2018.

22. World Health Organisation. Viral hepatitis C treatment in India. Available at: http://www.searo.who.int/india/mediacentre/events/2016/viral_hepatitis_c_treatment_in_india.pdf?ua=1 [Accessed on 9 December 2018].

23. Gakidou E, Nordhagen S, Obermeyer Z. Coverage of cervical cancer screening in 57 countries: low average levels and large inequalities. PLoS medicine. 2008 Jun;5(6).

24. Sharma M, Ying R, Tarr G, Barnabas R. Systematic review and meta-analysis of community and facility-based HIV testing to address linkage to care gaps in sub-Saharan Africa. Nature. 2015 Dec;528(7580):S77-85.

25. Coffin PO, Scott JD, Golden MR, Sullivan SD. Cost-effectiveness and population outcomes of general population screening for hepatitis C. Clinical infectious diseases. 2012 May 1;54(9):1259-71.

For peer review only

For peer review only - http://bmjopen.bmj.com/site/about/guidelines.xhtml

26. Bassett IV, Walensky RP. Integrating HIV screening into routine health care in resource-limited settings. Clinical Infectious Diseases. 2010 May 15;50(Supplement_3):S77-84.

27. Tang W, Chen W, Amini A, Boeras D, Falconer J, Kelly H, Peeling R, Varsaneux O, Tucker JD, Easterbrook P. Diagnostic accuracy of tests to detect Hepatitis C antibody: a meta-analysis and review of the literature. BMC infectious diseases. 2017 Nov 1;17(1):695.

28. Chugh Y, Dhiman R, Premkumar M, Prinja S, Singh Grover G, Bahuguna P. Real-world cost-effectiveness of pan-genotypic Sofosbuvir-Velpatasvir combination versus genotype dependent directly acting anti-viral drugs for treatment of hepatitis C patients in the universal coverage scheme of Punjab state in India. PLOS ONE. 2019;14(8):e0221769.

29. Prinja S, Bahuguna P, Duseja A, Kaur M, Chawla YK. Cost of Intensive Care Treatment for Liver Disorders at Tertiary Care Level in India. PharmacoEconomics - Open. 2017:1-12.

30. Prinja S, Sharma D, Aggarwal A, Bahuguna P, Sharma A, Rana S. Out-of-pocket expenditure for hospitalization in Haryana State of India: Extent, determinants & financial risk protection. Indian Journal of Medical Research. 2017;146(6):759.

31. Prinja S, Gupta A, Verma R, Bahuguna P, Kumar D, Kaur M et al. Cost of Delivering Health Care Services in Public Sector Primary and Community Health Centres in North India. PLOS ONE. 2016;11(8):0160986.

32. Kaur G, Prinja S, Malhotra P, Lad D, Prakash G, Khadwal A et al. Cost of Treatment of Multiple Myeloma in a Public Sector Tertiary Care Hospital of North India. 2018.

For peer review only

For peer review only - http://bmjopen.bmj.com/site/about/guidelines.xhtml

33. XE: Convert USD/INR. United States Dollar to India Rupee [Internet]. Xe.com. 2020 [cited 16 February 2020]. Available from: https://www.xe.com/currencyconverter/convert/?Amount=1&From=USD&To=INR.

34. National Sample Survey Office.Key Indicators of Social Consumption in India: Health (NSSO 71st Round, January–June 2014). New Delhi: NSSO;2015. 71 p.

35. Khuroo M, Khuroo N, Khuroo M. Diagnostic Accuracy of Point-of-Care Tests for Hepatitis C Virus Infection: A Systematic Review and Meta-Analysis. PLOS ONE. 2015;10(3):e0121450.

36. Shivkumar S, Peeling R, Jafari Y, Joseph L, Pant Pai N. Accuracy of Rapid and Point-of-Care Screening Tests for Hepatitis C. Annals of Internal Medicine. 2012;157(8):558.

37. Chong CA, Gulamhussein A, Heathcote EJ, Lilly L, Sherman M, Naglie G, et al. Healthstate utilities and quality of life in hepatitis C patients. Am J Gastroenterol. 2003;98:6308.

38. Thein H, Yi Q, Dore G, Krahn M. Estimation of stage-specific fibrosis progression rates in chronic hepatitis C virus infection: A meta-analysis and meta-regression. Hepatology. 2008;48(2):418-431.

39. Van der Meer A, Veldt B, Feld J, Wedemeyer H, Dufour J, Lammert F et al. Association Between Sustained Virological Response and All-Cause Mortality Among Patients With Chronic Hepatitis C and Advanced Hepatic Fibrosis. JAMA. 2012;308(24):2584.

40. Planas R, Ballesté B, Antonio Álvarez M, Rivera M, Montoliu S, Anton Galeras J et al. Natural history of decompensated hepatitis C virus-related cirrhosis. A study of 200 patients. Journal of Hepatology. 2004;40(5):823-830.

For peer review only

41. D'Amico G, Garcia-Tsao G, Pagliaro L. Natural history and prognostic indicators of survival in cirrhosis: A systematic review of 118 studies. Journal of Hepatology. 2006;44(1):217-231.

42. Altekruse S, McGlynn K, Reichman M. Hepatocellular Carcinoma Incidence, Mortality, and Survival Trends in the United States From 1975 to 2005. Journal of Clinical Oncology. 2009;27(9):1485-1491.

43. Registrar General & Census Commissioner of India. SRS life tables 2011-2015. http://www.censusindia.gov.in/Vital_Statistics/SRS_Life_Table/Srs_life_Table_2011-15.html [Accessed 20 August 2018].

44. Briggs A, Weinstein M, Fenwick E, Karnon J, Sculpher M, Paltiel A. Model Parameter Estimation and Uncertainty: A Report of the ISPOR-SMDM Modeling Good Research Practices Task Force-6. Value in Health. 2012;15(6):835-842.

45. Claxton K. Exploring Uncertainty in Cost-Effectiveness Analysis. PharmacoEconomics. 2008;26(9):781-798.

46. Revill P, Ochalek J, Lomas J, Nakamura R, Woods B, Rollinger A et al. Cost-effectiveness thresholds: guiding health care spending for population health improvement. Centre for Health Economics, University of York; 2015. 24 p.

47. Prinja S, Sundararaman T, Muraleedharan VR. Cost-effectiveness Threshold and Health Opportunity Cost. Economic & Political Weekly. 2020 Jan 11;55(2):19.

48. Punjab Population Sex Ratio in Punjab Literacy rate data 2011-2020 [Internet]. Census2011.co.in. 2020 [cited 19 February 2020]. Available from: https://www.census2011.co.in/census/state/punjab.html

49. Sullivan SD, Mauskopf JA, Annemans L, et al. Principles of good practice for budget impact analysis: report of the ISPOR Task Force on Good Research Practices—Budget Impact Analysis. Value Health. 2007;10(5):336-347.

For peer review only

For peer review only - http://bmjopen.bmj.com/site/about/guidelines.xhtml

50. Ministry of Health and family welfare, Government of Punjab. Budget 2020-21. Punjab: Government of Punjab; 2020 p. 73.

51. Pandey A, Ploubidis GB, Clarke L, Dandona L. Trends in catastrophic health expenditure in India: 1993 to 2014. Bulletin of the World Health Organization. 2018 Jan 1;96(1):18.

52. Lee R, Kottilil S, Wilson E. Sofosbuvir/velpatasvir: a pangenotypic drug to simplify HCV therapy. Hepatol Int. 2017;11(2):161-70.

53. Feld JJ, Jacobson IM, Hézode C, Asselah T, Ruane PJ, Gruener N, et al. Sofosbuvir and Velpatasvir for HCV Genotype 1, 2, 4, 5, and 6 Infection. N Engl J Med. 2015;373(27):2599-607.

54. Nelson DR, Cooper JN, Lalezari JP, et al. All-oral 12-week treatment with daclatasvir plus sofosbuvir in patients with hepatitis C virus genotype 3 infection: ALLY-3 phase 3 study. Hepatology. 2015;61:1127-1135.

55. Sullivan SD, Mauskopf JA, Augustovski F, Caro JJ, Lee KM, Minchin M, et al. Budget impact analysis—principles of good practice: report of the ISPOR 2012 Budget Impact Analysis Good Practice II Task Force. Value Health. 2014;17:4–14.

56. Barroy H, Sparkes S, Dale E, World Health Organization. Assessing fiscal space for health expansion in low-and-middle income countries: a review of the evidence. World Health Organization; 2016.

57. Srinath P, Kotasthane P, Kher D, Chhajer A. A Qualitative and Quantitative Analysis of Public Health Expenditure in India: 2005-06 to 2014-151.

58. GBD India Compare | IHME Viz Hub [Internet]. Vizhub.healthdata.org. 2020 [cited 14 October 2020]. Available from: https://vizhub.healthdata.org/gbd-compare/india

For peer review only - http://bmjopen.bmj.com/site/about/guidelines.xhtml

59. Kim DY, Han KH, Jun B, Kim TH, Park S, Ward T, Webster S, McEwan P. Estimating the cost-effectiveness of one-time screening and treatment for hepatitis C in Korea. PLoS One. 2017 Jan 6;12(1):e0167770.

60. Kim DD, Hutton DW, Raouf AA, Salama M, Hablas A, Seifeldin IA, Soliman AS. Cost-effectiveness model for hepatitis C screening and treatment: implications for Egypt and other countries with high prevalence. Global public health. 2015 Mar 16;10(3):296-317.

61. Nagai K, Ide K, Kawasaki Y, Tanaka-Mizuno S, Seto K, Iwane S, Eguchi Y, Kawakami K. Estimating the cost-effectiveness of screening for hepatitis C virus infection in Japan. Hepatology Research. 2020 May;50(5):542-56.

62. Coffin PO, Scott JD, Golden MR, Sullivan SD. Cost-effectiveness and population outcomes of general population screening for hepatitis C. Clinical infectious diseases. 2012 May 1;54(9):1259-71.

63. Pike J, Grosse S. Friction Cost Estimates of Productivity Costs in Cost-of-Illness Studies in Comparison with Human Capital Estimates: A Review. Applied Health Economics and Health Policy. 2018;16(6):765-778.

For peer review only - http://bmjopen.bmj.com/site/about/guidelines.xhtml

Tables

Table 1: Demographic, epidemiologic, effectiveness and cost-related parameters.

Parameters	Base value	Lower limit	Upper limit	Distribution	Source
Epidemiological parameters					
Viraemic prevalence of HCV in Punjab	0.026	0.0234	0.0286	Uniform	12,13,15
19-29 years	0.012	0.005	0.019	Uniform	12,13,15
30-39 years	0.031	0.018	0.043	Uniform	12,13,15
40-49 years	0.047	0.031	0.062	Uniform	12,13,15
50-59 years	0.045	0.027	0.062	Uniform	12,13,15
>/=60 years	0.027	0.014	0.039	Uniform	12,13,15
Stage-wise distribution of HCV at diagnosis					
F0	0.325	0.30875	0.34125	Uniform	16,28
F1	0.325	0.30875	0.34125	Uniform	16,28
F2	0.1	0.095	0.105	Uniform	16,28
F3	0.1	0.095	0.105	Uniform	16,28
F4	0.12	0.114	0.126	Uniform	16,28
DC	0.03	0.0285	0.0315	Uniform	16,28
Proportion of population that is RBV tolerant	0.9	0.81	0.99	Uniform	16,28
Clinical parameters					Expert opinion
F0-F3					
Number of OPD contacts required	3	2	4	Uniform	

For peer review only

For peer review only - http://bmjopen.bmj.com/site/about/guidelines.xhtml

Proportion of patients requiring hospitalisations	0.05	0.01	0.1	Uniform
Number of hospitalisations per patient per year	2	1	4	Uniform
F4				
Number of OPD contacts required	3	2	4	Uniform
Proportion of patients requiring hospitalisations	0.05	0.01	0.1	Uniform
Number of hospitalisations per patient per year	2	1	4	Uniform
DC				
Number of OPD contacts required	12	6	18	Uniform
Proportion of patients requiring hospitalisations	0.8	0.7	0.9	Uniform
Number of hospitalisations per patient per year	6	4	8	Uniform
HCC				
Number of OPD contacts required	12	6	18	Uniform
Proportion of patients requiring hospitalisations	0.6	0.5	0.7	Uniform
Number of hospitalisations per patient per year	2	1	4	Uniform

For peer review only

For peer review only - http://bmjopen.bmj.com/site/about/guidelines.xhtml

Effectiveness parameters					
Quality of life weights					
F0 - F3	0.63	0.57	0.70	Beta	28
F4	0.56	0.51	0.61	Beta	28
DC	0.44	0.38	0.49	Beta	28
HCC	0.44	0.38	0.49	Beta	28
F0-F3 (post SVR)	1	0.83	1	Beta	28,37
Discount rate	0.03	0.02	0.08	Uniform	18-21
Transition probabilities					
F0 to F1	0.177	0.104	0.13	Beta	39,40
F1 to F2	0.085	0.075	0.096	Beta	39,40
F2 to F3	0.12	0.109	0.133	Beta	39,40
F3 to F4	0.116	0.104	0.129	Beta	39,40
F4 to DC	0.035	0.027	0.043	Beta	39,40
F4 to DC (post SVR)	0.002	0.0001	0.005	Beta	39,40
F4 to HCC	0.024	0.018	0.031	Beta	39,40
F4 to HCC (post SVR)	0.005	0.001	0.009	Beta	39,40
DC to HCC	0.068	0.03	0.083	Beta	39,40
DC to HCC (post SVR)	0.03	0.0225	0.0375	Beta	39,40
Probability of dying due to HCV					
F0 – F4	0	0	0	Uniform	28
DC	0.216	0.162	0.27	Uniform	41-43
HCC	0.411	0.31	0.51	Uniform	41-43

For peer review only - http://bmjopen.bmj.com/site/about/guidelines.xhtml

SVR rates (%)					
SOF+DCV (12 weeks)	74	66.5	73.5	Uniform	16,28
SOF+VEL (12 weeks)	84	79.8	88.2	Uniform	16,28
SOF+VEL (24 weeks)	81	76.95	85.05	Uniform	16,28
SOF+VEL+RBV (12 weeks)	84	79.8	88.2	Uniform	16,28
Sensitivity ELISA	0.952	0.918	0.972	Beta	35,36
Specificity ELISA	0.986	0.976	0.993	Beta	35,36
Sensitivity RDT	0.984	0.889	0.998	Beta	35,36
Specificity RDT	0.986	0.949	0.996	Beta	35,36
Coverage parameters					
Treatment coverage in do-nothing scenario (%)	5	0	10	Uniform	Author's assumption
Coverage of screening in scenario I & II (%)	30	20	50	Uniform	Author's assumption
Loss to follow up at each step (%)	10	5	20	Uniform	Author's assumption
Cost parameters					
Drug costs-12 weeks (□)					
SOF+DCV	4509	3607.2	4509	Gamma	16
SOF+VEL	13104	10483.2	13104	Gamma	16
SOF+VEL+RBV	16818	13454.4	16818	Gamma	16
Cost of diagnostic tests (□)					

For peer review only

ELISA	72.95	58.36	109.425	Gamma	16
RDT	39	31.2	58.5		16
HCV-RNA	880	704	1320	Gamma	16
Routine tests (CBC, LFT, Creatinine)	500	400	750	Gamma	16
Cirrhosis evaluation (Fibro-scan)	350	280	525	Gamma	16
Genotyping	895	716	1342.5	Gamma	16
Cost per OPD consultation (□)					
Primary	1686.3	1180.41	2192.19	Gamma	29-32
Secondary	1734	1213.8	2254.2	Gamma	29-32
Tertiary	2024	1416.8	2631.2	Gamma	29-32
Cost per patient hospitalisation (□)					
Primary	6347.1	4442.97	8251.23	Gamma	29-32
Secondary	7597	5317.9	9876.1	Gamma	29-32
Tertiary	18693	13085.1	24300.9	Gamma	29-32
Per unit cost of integrated training (□)					
District	50000	40000	75000	Gamma	Expert opinion
State	308000	246400	462000	Gamma	Expert opinion
Per district cost of IEC/BCC (□)	4000	3200	6000	Gamma	Expert opinion

HCV-Hepatitis C virus, DC-Decompensated cirrhosis, HCC-Hepatocellular carcinoma, SOF-Sofosbuvir, LDV-Ledipasvir, RBV-Ribavirin, DCV-Daclatasvir, VEL-Velpatasvir, GT-genotype, OPD- Out-patient Department, IPD- In-patient Department, ELISA: Enzyme Linked Immunosorbent Assay, RDT: Rapid Diagnostic Tests, IEC/BCC: Information-education-counselling and Behaviour change communication, □: Indian rupee.

For peer review only

Table 2: Cost and Effects of Screening and treating Chronic hepatitis C virus infected Patients

**No-Screening**	
Life years per patient	15.75 (11.79-23.85)
QALY per patient	15.57 (11.63-23.60)
Cost per patient (□)	2,775 (1,549-4,898)
**Scenario I**	
Life years per patient	15.92 (11.79-23.86)
QALY per patient	15.78 (11.65-23.67)
Cost per patient (□)	998 (456-1,932)
**Scenario II**	
Life years per patient	15.75 (11.79-23.86)
QALY per patient	15.60 (11.65-23.63)
Cost per patient (□)	947 (468-1,837)
**Incremental gains**	
Scenario I- No screening	
Life years gained	0.0046 (0.0013-0.011)
QALYs gained	0.028 (0.008-0.061)
Cost difference (□)	-1,810 (-3,376 to – 867)
Scenario II- No screening	
Life years gained	0.0045 (0.001-0.012)
QALYs gained	0.027 (0.008-0.061)
Cost difference (□)	-1,812 (-3,468 to -850)

HCV: Hepatitis C virus, QALY: Quality adjusted life year, □: Indian rupee

For peer review only

For peer review only - http://bmjopen.bmj.com/site/about/guidelines.xhtml

Table 3 A: Budget Impact Assessment: Incremental Budgetary estimates spread across the time horizon (year 1 to year 5) in million rupees (million US $)

		ELISA	RDT
**18 Years and older**	Year 1	1779.42 (25.24)	1586.32 (22.5)
	Year 2	52.81 (0.75)	47.1 (0.67)
	Year 3	53.05 (0.75)	47.54 (0.67)
	Year 4	53.44 (0.76)	48.27 (0.68)
	Year 5	53.93 (0.76)	49.16 (0.7)
**40-45 Years**	Year 1	208.7 (2.96)	190.46 (2.7)
	Year 2	45.83 (0.65)	41.83 (0.59)
	Year 3	46.07 (0.65)	42.07 (0.6)
	Year 4	46.46 (0.66)	42.46 (0.6)
	Year 5	46.94 (0.67)	42.94 (0.61)

ELISA: Enzyme Linked Immunosorbent Assay, RDT: Rapid Diagnostic Tests, US $: United States Dollar.

For peer review only

Table 3 B: Budget Impact Assessment: Resource wise disaggregated estimates of budget for the 1st year at base coverage (30%) in million rupees (million US $)

	40-45 Years		18 Years and older	
	**ELISA**	**RDT**	**ELISA**	**RDT**
**HR**	1.314 (0.019)	1.314 (0.019)	3.367 (0.048)	3.367 (0.048)
**IEC/BCC**	0.088 (0.001)	0.088 (0.001)	0.088 (0.001)	0.088 (0.001)
**Screening Test**	35.94 (0.51)	17.7 (0.251)	380.39 (5.396)	187.29 (2.657)
**Confirmatory Test**	22.12 (0.314)	22.12 (0.314)	180.16 (2.555)	180.16 (2.555)
**Drugs**	149.24 (2.117)	149.24 (2.117)	1215.42 (17.24)	1215.42 (17.24)
**Total**	208.702 (2.96)	190.462 (2.702)	1779.425 (25.24)	1586.325 (22.501)

HR: Human resources, IEC/BCC: Information-Education-Counselling-Behaviour-Change-Communication, ELISA: Enzyme Linked Immunosorbent Assay, RDT: Rapid Diagnostic Tests, US $: United States Dollar.

For peer review only

For peer review only - http://bmjopen.bmj.com/site/about/guidelines.xhtml

771 **Table 3 C: Budget Impact Assessment: Estimates for different screening scenarios in million**
772 **rupees (million US $)**

	**18 Years and older**		**Age cohort 40-45 years**	
**Coverage of screening (%)**	**ELISA**	**RDT**	**ELISA**	**RDT**
10	593.14 (8.41)	528.78 (7.5)	69.57 (0.99)	63.49 (0.9)
20	1186.28 (16.83)	1057.54 (15)	139.13 (1.97)	126.97 (1.8)
30	1779.42 (25.24)	1586.32 (22.5)	208.7 (2.96)	190.46 (2.7)
50	2965.69 (42.07)	2643.87 (37.5)	347.84 (4.93)	317.44 (4.5)
70	4151.97 (58.89)	3701.41 (52.5)	486.98 (6.91)	444.42 (6.3)
100	5931.39 (84.13)	5287.73 (75)	695.68 (9.87)	634.88 (9.01)

773 ELISA: Enzyme Linked Immunosorbent Assay, RDT: Rapid Diagnostic Tests, US $: United States Dollar.

774

775

776

777

778

779

780

781

782

783

For peer review only

For peer review only - http://bmjopen.bmj.com/site/about/guidelines.xhtml

Figures

Figure 1: Treatment guidelines for management of Chronic Hepatitis C virus infection.[61]

Figure 2 : Decision tree depicting the screening pathway for different scenarios.

Figure 3: Markov model depicting the natural history of Hepatitis C infection.

Figure 4: Cost-effectiveness planes for scenario I and II vs No screening.

For peer review only

For peer review only - http://bmjopen.bmj.com/site/about/guidelines.xhtml

Figure 1: Treatment guidelines for management of Chronic Hepatitis C virus infection.[61]

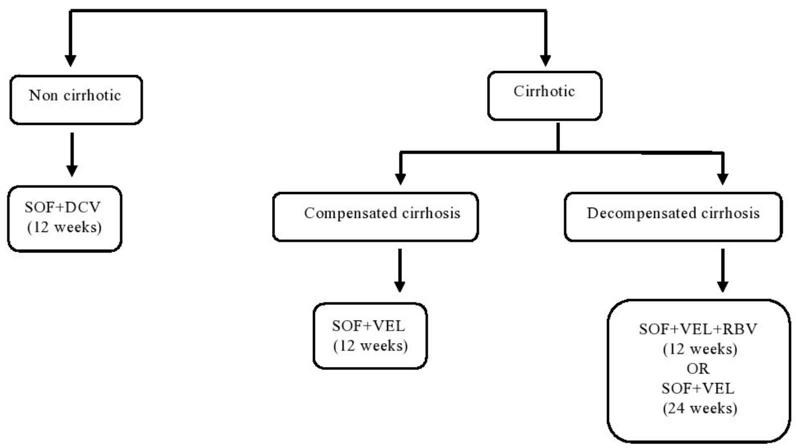

SOF-Sofosbuvir, LDV-Ledipasvir, RBV-Ribavirin, DCV-Daclatasvir, VEL-Velpatasvir

Treatment guidelines for management of Chronic Hepatitis C virus infection

321x294mm (96 x 96 DPI)

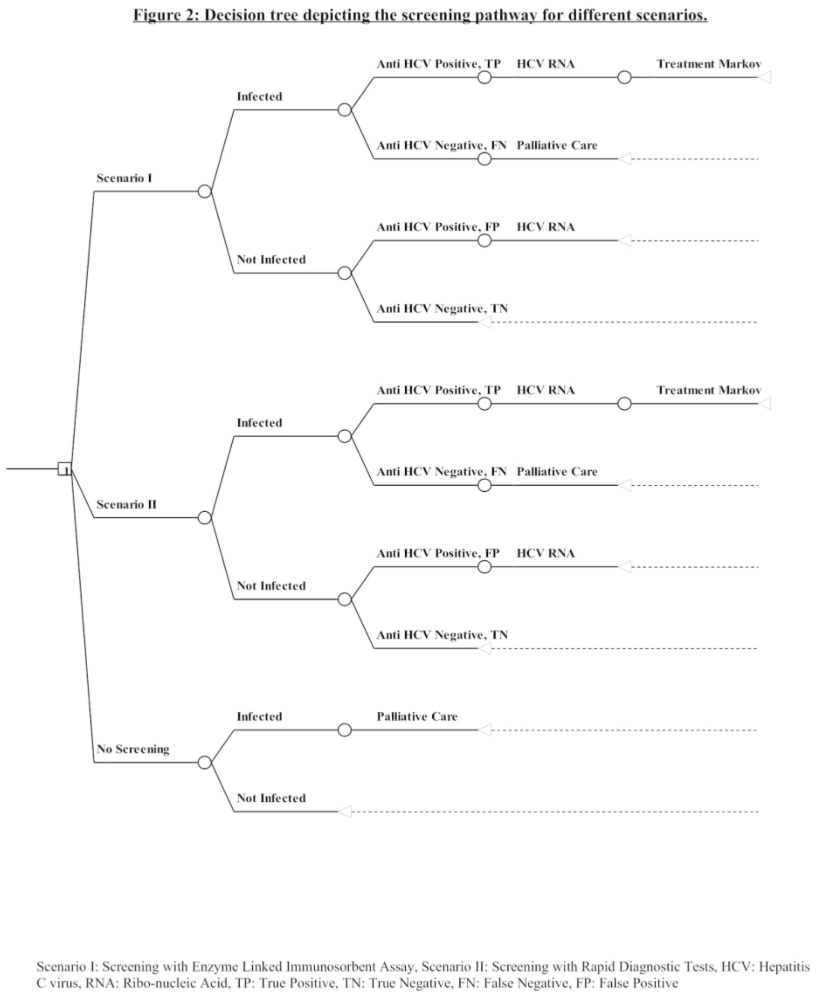

Decision tree depicting the screening pathway for different scenarios

583x825mm (72 x 72 DPI)

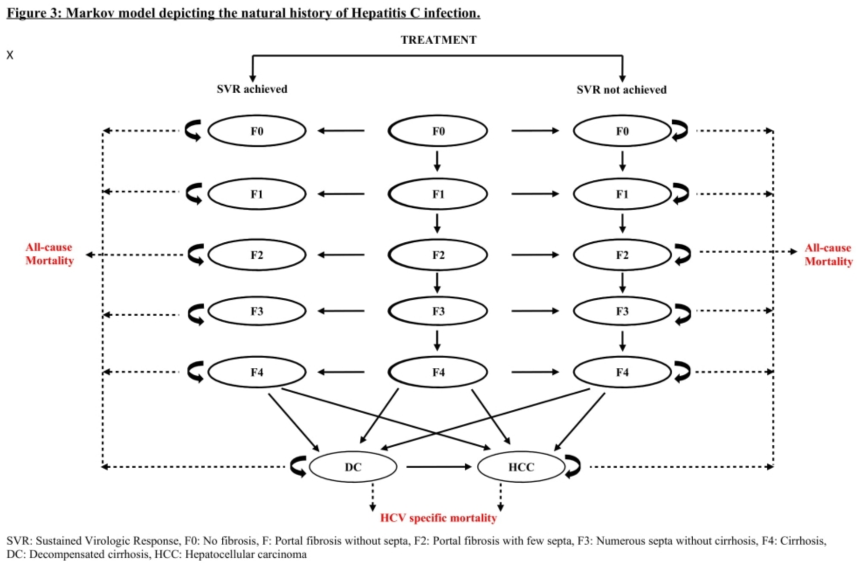

Markov model depicting the natural history of Hepatitis C infection

825x583mm (72 x 72 DPI)

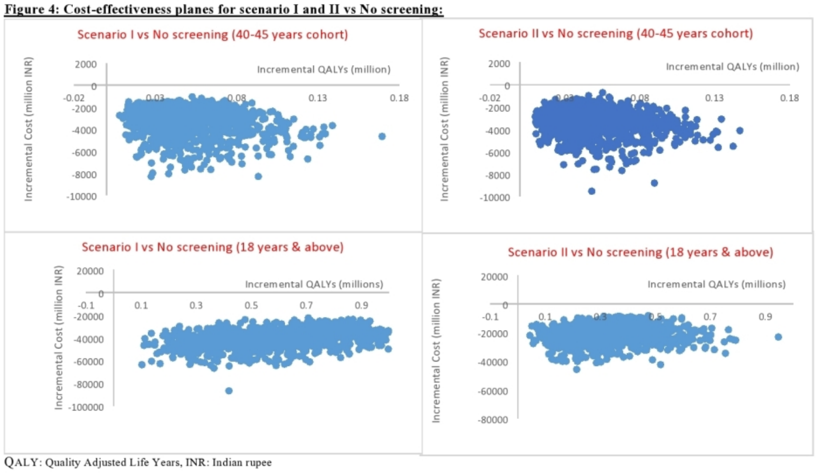

Cost-effectiveness planes for scenario I and II vs No screening

618x437mm (72 x 72 DPI)

CHEERS checklist: Cost-effectiveness and budget impact analysis of screening and treating Hepatitis C infected population vs no screening and treatment in Punjab state of India

Section/item	Item No	Recommendation	Reported on page No/ line No
**Title and abstract**			
Title	1	Identify the study as an economic evaluation or use more specific terms such as "cost-effectiveness analysis", and describe the interventions compared.	Page 1, Line 1-2
Abstract	2	Provide a structured summary of objectives, perspective, setting, methods (including study design and inputs), results (including base case and uncertainty analyses), and conclusions.	Page 3, Line 83-104
**Introduction**			
Background and objectives	3	Provide an explicit statement of the broader context for the study.	Page 5, Line 143-151
		Present the study question and its relevance for health policy or practice decisions.	Page 6, Line 164-170
**Methods**			
Target population and subgroups	4	Describe characteristics of the base case population and subgroups analysed, including why they were chosen.	Page 5, Line 146-149; Page 9, Line 227-233
Setting and location	5	State relevant aspects of the system(s) in which the decision(s) need(s) to be made.	Page 5, Line 146-149, 152-155; Page 6, Line 159-163
Study perspective	6	Describe the perspective of the study and relate this to the costs being evaluated.	Page 9, Line 244-149; Page 10, Line 261-264
Comparators	7	Describe the interventions or strategies being compared and state why they were chosen.	Page 7, Line 190-203; Page 8, Line 204-217
Time horizon	8	State the time horizon(s) over which costs and consequences are being evaluated and say why appropriate.	Page 7, Line 183-184
Discount rate	9	Report the choice of discount rate(s) used for costs and outcomes and say why appropriate.	Page 7, Line 185-189
Choice of health outcomes	10	Describe what outcomes were used as the measure(s) of benefit in the evaluation and their relevance for the type of analysis performed.	Page 7, Line 184-185, 187-189
Measurement of effectiveness	11a	*Single study-based estimates:* Describe fully the design features of the single effectiveness study and why the single study was a sufficient source of clinical effectiveness data.	Page 10, Line 267-270; Page 11, Line 278-279; Page 17, Line 419-422
	11b	*Synthesis-based estimates*: Describe fully the methods used for identification of included studies and synthesis of clinical effectiveness data.	
Measurement and valuation of preference-based outcomes	12	If applicable, describe the population and methods used to elicit preferences for outcomes.	Page 10, Line 270-274
Estimating resources and costs	13a	*Single study-based economic evaluation:* Describe approaches used to estimate resource use associated with the alternative interventions. Describe primary or secondary research methods for valuing each resource item in terms of its unit cost. Describe any adjustments made to approximate to opportunity costs.	Page 9, Line 245-250; Page 10, Line 251-265
	13b	*Model-based economic evaluation:* Describe approaches and data sources used to estimate resource use associated with model health states. Describe primary or secondary research methods for valuing each resource item in terms of its unit cost. Describe any adjustments made to approximate to opportunity costs.	
Currency, price date, and conversion	14	Report the dates of the estimated resource quantities and unit costs. Describe methods for adjusting	Page 10, Line 260-261

Section/item	Item No	Recommendation	Reported on page No/ line No
		estimated unit costs to the year of reported costs if necessary. Describe methods for converting costs into a common currency base and the exchange rate.	
Choice of model	15	Describe and give reasons for the specific type of decision-analytical model used. Providing a figure to show model structure is strongly recommended.	Page 8, Line 219-220; Figure 2 (A & B)
Assumptions	16	Describe all structural or other assumptions underpinning the decision-analytical model.	Page 7, Line 194-196, 203; Page8, Line 205; Page 11, Line 284-287
Analytical methods	17	Describe all analytical methods supporting the evaluation. This could include methods for dealing with skewed, missing, or censored data; extrapolation methods; methods for pooling data; approaches to validate or make adjustments (such as half cycle corrections) to a model; and methods for handling population heterogeneity and uncertainty.	Page 11, Line 290-297; Page 12, Line 298-321; Page 13, Line 322-340
**Results**			
Study parameters	18	Report the values, ranges, references, and, if used, probability distributions for all parameters. Report reasons or sources for distributions used to represent uncertainty where appropriate. Providing a table to show the input values is strongly recommended.	Table 1
Incremental costs and outcomes	19	For each intervention, report mean values for the main categories of estimated costs and outcomes of interest, as well as mean differences between the comparator groups. If applicable, report incremental cost-effectiveness ratios.	Table 2
Characterising uncertainty	20a	*Single study-based economic evaluation:* Describe the effects of sampling uncertainty for the estimated incremental cost and incremental effectiveness parameters, together with the impact of methodological assumptions (such as discount rate, study perspective).	Page 14, Line 363-368; Figure 3
	20b	*Model-based economic evaluation:* Describe the effects on the results of uncertainty for all input parameters, and uncertainty related to the structure of the model and assumptions.	
Characterising heterogeneity	21	If applicable, report differences in costs, outcomes, or cost-effectiveness that can be explained by variations between subgroups of patients with different baseline characteristics or other observed variability in effects that are not reducible by more information.	Page 14, Line 363-368; Figure 3; Table 3 (A, B & C)
**Discussion**			
Study findings, limitations, generalisability, and current knowledge	22	Summarise key study findings and describe how they support the conclusions reached. Discuss limitations and the generalisability of the findings and how the findings fit with current knowledge.	Page 15, Line 381-385, 387-392; Page 16, Line 393-394, 408-417; Page 17, Line 418-441; Page 18, Line 442-443
**7Other**			
Source of funding	23	Describe how the study was funded and the role of the funder in the identification, design, conduct, and reporting of the analysis. Describe other non-monetary sources of support.	Page 1, Line 30-38
Conflicts of interest	24	Describe any potential for conflict of interest of study contributors in accordance with journal policy. In the absence of a journal policy, we recommend authors comply with International Committee of Medical Journal Editors recommendations.	Page 1, Line 40-41

For peer review only
